# Modulation of Photoluminescence and Solar Thermal Energy Storage in Norbornadiene–Quadricyclane Dimers

**DOI:** 10.1002/anie.202516629

**Published:** 2025-11-04

**Authors:** Rebecca J. Salthouse, Jacob L. Elholm, Irene Cortellazzi, Helen Hölzel, Pedro Ferreira, Lorette Fernandez, Marc K. Etherington, Kasper Moth‐Poulsen

**Affiliations:** ^1^ Department of Chemical Engineering Universitat Politècnica de Catalunya EEBE, Eduard Maristany 10–14 Barcelona 08019 Spain; ^2^ Department of Environmental and Earth Sciences Università degli Studi di Milano‐Bicocca Piazza dell'Ateneo Nuovo, 1 Milano 20126 Italy; ^3^ Justus‐Liebig‐Universität Giessen Institut Organische Chemie Heinrich‐Buff‐Ring 17 35392 Giessen Germany; ^4^ School of Engineering, Physics and Mathematics Northumbria University Ellison Place Newcastle upon Tyne NE1 8ST UK; ^5^ The Institute of Materials Science of Barcelona ICMAB‐CSIC Bellaterra Barcelona 08193 Spain; ^6^ Department of Chemistry and Chemical Engineering Chalmers University of Technology Gothenburg SE‐41296 Sweden; ^7^ Catalan Institution for Research & Advanced Studies ICREA Pg. Lluis Companys 23 Barcelona 08010 Spain

**Keywords:** Fluorescence, Norbornadiene–quadricyclane, Photoswitches, Solar energy storage, Thermal energy release

## Abstract

The norbornadiene/quadricyclane (NBD/QC) photoswitch pair is a promising system for molecular solar thermal (MOST) energy storage. Multichromophoric systems with two or more photoswitches can offer red‐shifted absorption, higher energy densities, and additional functionality. Here, a series of *ortho*‐ and *para*‐substituted NBD dimers bearing methoxy, hexoxy (for solubility), and cyano groups were synthesised and evaluated for their MOST properties. Compared to monomers, the dimers display red‐shifted absorption and improved solar spectrum match, with onsets between 448–488 nm, owing to their donor–acceptor design and extended conjugation. A key finding is the tunable relationship between molecular structure, photoluminescence and photoisomerisation: *para*‐dimers exhibit efficient fluorescence, whilst *ortho*‐dimers are superior photoswitches with quantum yields of isomerisation, Φ_i_, up to 63%. Solvent choice further modulates behaviour; Φ_i_ is higher in acetonitrile, whereas fluorescence is more efficient in toluene. This interplay allows tailoring for specific functions. The best‐performing photoswitches were studied in a liquid‐chip device, achieving a record solar conversion efficiency of 2.9%. Catalytic back‐conversion using cobalt phthalocyanine on carbon and macroscopic heat release experiments at 0.1 M yielded a 5.78 °C temperature increase. This first experimental macroscopic heat release of a dimeric system provides important insights into design challenges and opportunities for advancing multichromophoric systems towards MOST applications.

## Graphical abstract

Multichromophoric donor–acceptor norbornadiene switches exhibit red‐shifted absorption, tunable photoisomerisation and fluorescence, and record solar energy storage performance. *O* *rtho*‐dimers act as efficient photoswitches, whilst *para*‐dimers are highly emissive. Solvent choice further tunes quantum yields, and a device efficiency of 2.9% with measurable heat release highlights their potential for MOST.

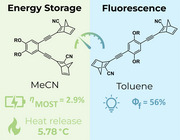

## Introduction

Renewable energy solutions are paramount in tackling the rising global energy crisis. MOlecular Solar Thermal (MOST) systems are emerging as promising candidates to both capture and store solar energy.^[^ ^1^, ^2^, ^3^, ^4^, ^5^, ^6^ ^]^ They comprise molecular photoswitches that absorb light, store it in the form of a metastable photoisomer, and release the energy as heat on‐demand in a closed cycle. Examples of such photoswitches that have been studied in this context are azobenzenes,^[^ ^7^, ^8^, ^9^, ^10^, ^11^, ^12^, ^13^ ^]^ dihydroazulene/vinylheptafulvenes (DHA/VHF),^[^ ^14^, ^15^ ^]^ (fulvalene)tetracarbonyl‐diruthenium derivatives,^[^ ^16^ ^]^ norbornadiene/quadricyclane (NBD/QC)^[^ ^17^, ^18^, ^19^, ^20^, ^21^ ^]^ or bicyclooctadiene/tetracyclooctane (BOD/TCO) couples,^[^ ^22^, ^23^ ^]^ and anthracene.^[^ ^24^, ^25^, ^26^ ^]^

The NBD/QC couple in particular has received a lot of attention due to its favourable energy storage density of up to 1 MJ kg^−1^, owing to the highly strained QC photoisomer.^[^ ^27^ ^]^ Various efforts have been made to optimise the storage properties of NBDs: to red‐shift the absorption from the UV to better overlap with the solar spectrum, to increase the thermal half‐life (*t*_1/2_) and the quantum yield of photoisomerisation (Φ_i_), thereby increasing the energy storage capabilities.^[^ ^1^ ^]^ Recent results have shown a record solar energy capture efficiency of 2.3%, as well as offering a cooling effect when combined with existing photovoltaic (PV) technology to boost PV cell efficiency and increase the efficiency of the hybrid device relative to the individual parts, establishing the combination of NBD with PV as a competitive candidate for renewable energy storage solutions.^[^ ^28^ ^]^

In order to red‐shift the absorption of NBD derivatives, donor–acceptor systems have been utilised,^[^ ^18^ ^]^ as well as acceptor–acceptor systems more recently,^[^ ^29^ ^]^ though red‐shifted absorption is often accompanied by a decrease in Φ_i_ and thermal half‐life. Multichromophoric photoswitches, where there are two or more photochromic units within the same molecule, can also be exploited to achieve red‐shifted absorption due to the extended π‐conjugation offered by these systems.^[^ ^30^, ^31^, ^32^, ^33^, ^34^, ^35^, ^36^ ^]^ Moreover, the fact that some of the molecular weight is ‘shared’ across two or more photoswitches can lead to increased energy storage densities, as well as the multi‐switch nature providing alternative applications such as data storage.^[^ ^37^, ^38^ ^]^

**Figure 1:**
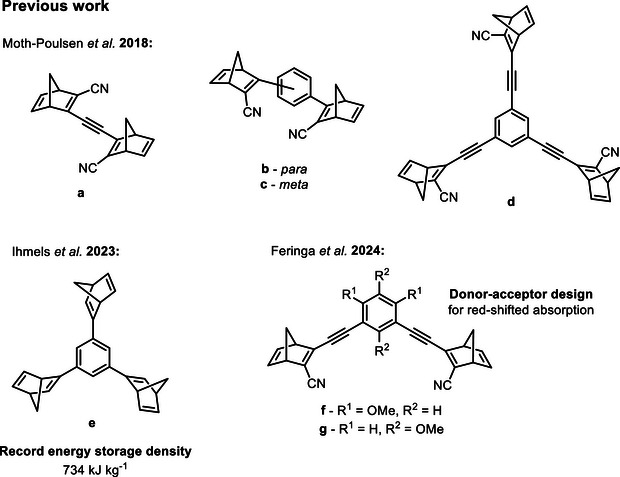
Representative examples of previously reported NBD dimers and trimers.

Previously reported dimer and trimer NBD systems are shown in Figure 1. Moth–Poulsen and co‐workers^[^ ^39^ ^]^ reported bis‐ and tris‐NBD systems in 2018 featuring cyano‐acceptor groups, with calculated energy storage densities up to 927 kJ kg^−1^ (for compound **a**) and measured densities up to 559 kJ kg^−1^ (compound **c**), greatly surpassing the outlined target for an efficient MOST system of 300 kJ kg^−1^.^[^ ^17^ ^]^ Ihmels et al. later studied a similar series of compounds but with only one substituent on the NBD double bond, i.e., without the cyano‐groups, and with the NBDs directly connected to the core aromatic units, i.e., without acetylene linkers.^[^ ^40^ ^]^ These designs led to a record high energy density of 734 kJ kg^‑1^ for the trimer **d** due to the lower molecular weight of the system. Nevertheless, the absorption onset is limited to the range 332−386 nm compared to 362−411 nm for those with cyano‐substituents. The group is currently investigating photosensitisation as a method to utilise more of the solar spectrum.^[^ ^41^ ^]^ A donor–acceptor design was employed by Feringa and co‐workers for *para*‐ and *meta*‐substituted NBD dimers featuring methoxy‐ and cyano‐substituents (compounds **f** and **g**, Figure 1).^[^ ^42^ ^]^ The absorption spectra were particularly red‐shifted for the *para*‐derivatives, with absorption maxima up to 404 nm and reported onsets reaching 536 nm, without compromising the thermal half‐life.

**Figure 2:**
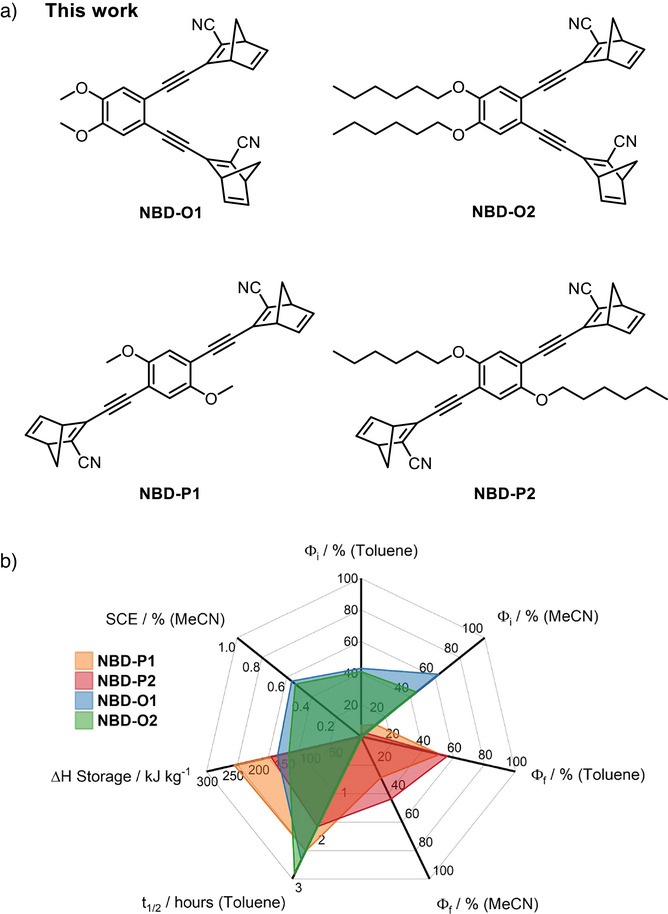
a) *Ortho*‐ and *para*‐substituted NBD dimers synthesised and studied in this work. Out of these, **NBD‐P1** has been previously reported.^[^ ^42^ ^]^ The dimers feature a donor‐acceptor design with alkoxy chains for increased solubility. b) Radar plot demonstrating the key parameters across the four photoswitches: photoisomerisation quantum yield (Φ_i_), fluorescence quantum yield (Φ_f_), half‐lifetime (t_1/2_), energy storage (ΔH_storage_) and solar conversion efficiency (SCE).

Here, we study for the first time donor–acceptor NBD dimers connected via a phenyl linker bis‐substituted in the *ortho*‐positions and compare them to their *para*‐substituted analogues. The dimers feature methoxy and cyano‐substituents (Figure 2a) for red‐shifted absorption and improved solar spectrum match. We recognise that solubility is also an important factor when considering the applicability of these materials for solution‐state MOST devices. Despite the fact that adding alkoxy chains to aid solubility will increase the molecular weight of the dimers and in turn lead to lower energy storage densities, a balance must be found for application in heat release devices in order to measure the highest heat release possible in solution. In this regard, dimers with hexoxy groups have been synthesised and characterised. With these photoswitches in hand, a complete characterisation was performed across several key metrics which are summarised in Figure 2b.

We study photoswitching dynamics in both toluene and acetonitrile,^[^ ^43^ ^]^ uncovering the interplay between energy storage and photoluminescence as a competitive excited‐state process to photoisomerisation, which is particularly efficient for the *para*‐linked NBD systems. We show how the photoconversion can be tuned by chemical design and tailored use of solvents; acetonitrile promotes photoisomerisation and hence the energy storage capability, whilst fluorescence is shown to be more efficient in toluene (Figure 2). Moreover, we demonstrate the performance of the as‐synthesised NBD dimers under real‐life operation by applying the most‐soluble and best‐performing dimers in a liquid‐flow chip to measure the solar conversion efficiencies^[^ ^44^ ^]^ experimentally for the first time for a multichromophoric photoswitch. Finally, we present the catalytic back‐conversion as a method to trigger the energy release and for the first time investigate an NBD dimer for catalytic on‐demand heat release. This study explores the structure–property relationships of dimer systems and examines the challenges posed by multichromophoric architectures for practical application in MOST devices, drawing fundamental links between molecular structure and MOST performance and highlighting key considerations and directions for future improvements in next‐generation system design.

## Results and Discussion

### Synthesis and Photophysical Properties

**Figure 3:**
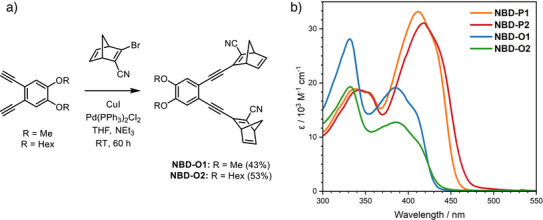
a) Synthesis of the *ortho*‐dimers from 3‐bromo‐2‐cyanonorbornadiene. b) Absorption spectra for all NBD dimers in toluene.

The synthesis of all the NBD dimers is reported in the Supporting Information and proceeds through a Sonogashira coupling between 3‐bromo‐2‐cyanonorbornadiene and the respective acetylene precursors, as shown in Figure 3 for the *ortho*‐dimers. It is worth noting that previously, 3‐chloro‐2‐cyanonorbornadiene was used instead of 3‐bromo‐2‐cyanonorbornadiene, though the synthesis of this intermediate is more time‐consuming as it first requires the synthesis of 2‐bromo‐3‐chloronorbornadiene.^[^ ^18^ ^]^ All the new materials were fully characterised by ^1^H and ^13^C NMR spectroscopy and high‐resolution mass spectrometry, with characterisation data and spectra provided in the Supporting Information.

The UV–vis absorption spectra of all the NBD dimers in toluene are shown in Figure 3, and the absorption onsets (λ_onset_), absorption maxima, and molar extinction coefficients can be found in Table 1. The spectra in acetonitrile were also recorded and can be found in Section 8 of the Supporting Information. The absorption of all switches extends to the visible region of the spectrum, as desired for MOST applications to harness all portions of the solar spectrum. The absorption onsets range from 448 to 488 nm and are red‐shifted in the order **NBD‐O1 < NBD‐O2 < NBD‐P1 < NBD‐P2**, i.e., increased red‐shift for the *para*‐substituted derivatives due to their linear conjugation pathway. Here, we define λ_onset_ as the absorption wavelength where log(ε) = 2. The absorption maximum for the *ortho‐*NBDs is the higher energy band, whilst for the *para*‐derivatives it is the lowest energy band. The hexoxy chains lead to a slight red‐shift in the absorption compared to the methoxy‐groups, and it is apparent that the *para*‐derivatives not only display red‐shifted absorption spectra but also the molar extinction coefficients of the lower energy band are approx. double those of the respective *ortho*‐substituted NBDs, leading to a better overlap with the solar spectrum.

**Table 1:** Absorbance wavelengths with extinction coefficients, onset of absorption, thermal –half‐life of QC‐QC (t_1/2_), quantum yield of photoisomerisation (Φ_i_) of the NBD‐NBD to NBD‐QC process, and fluorescence quantum yield (Φ_f_) both in toluene and acetonitrile.

Dimer	λ_abs_ / nm (ε / M^−1^ cm^−1^) *λ_max_	λ_onset_ / nm	t_1/2_ at 25 °C / hours	Φ_i_ toluene	Φ_i_ MeCN	Φ_f_ toluene	Φ_f_ MeCN
**NBD‐P1**	337 (18 200), 354 (17 800), 411^*)^ (32 900), 430 (27 500)	473	2.41	7%	12%	51%	29%
**NBD‐P2**	343 (18 700), 356 (17 700), 417^*)^ (30 700), 433 (27 400)	488	1.90	1.5%	3.5%	56%	44%
**NBD‐O1**	331 (27 900), 384^*)^ (19 100), 408 (14 800)	448	2.61	29% (405 nm) 43% (365 nm)	48% (405 nm) 63% (365 nm)	0	1%
**NBD‐O2**	333 (19 000), 386^*)^ (12 600), 411 (9280)	461	2.90	29% (405 nm) 41% (365 nm)	35% (405 nm) 45% (365 nm)	1%	1%

*) Absorbance maximum of the lowest‐energy band (*λ*_max_). The absorbance onset (*λ*_onset_) is defined as log(ε) = 2. All parameters are reported in toluene unless otherwise stated.

### Photoswitching

**Figure 4:**
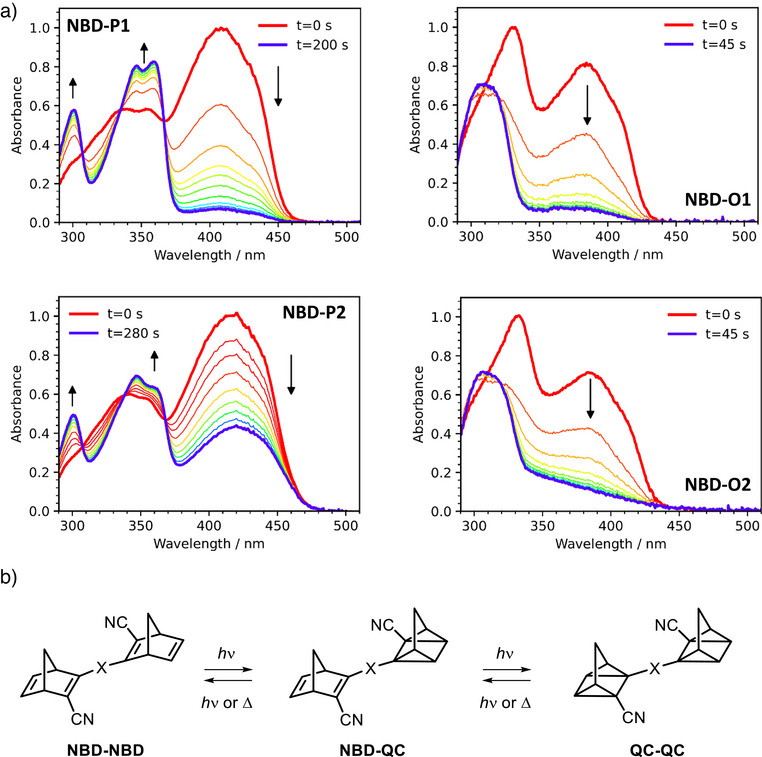
a) UV–vis spectral change of NBD dimers upon irradiation with 365 nm (**NBD‐O1** and **NBD‐O2**) or 405 nm (**NBD‐P1** and **NBD‐P2**) LED in toluene at 25 °C. b) Scheme to show the structure of each possible isomer.

The switching properties of the NBD dimers were studied by UV–vis spectroscopy. Three possible isomers exist for each dimer, namely the NBD‐NBD, NBD‐QC and QC‐QC forms (Figure 4b). Upon irradiation in an in‐house automated setup^[^ ^45^ ^]^ with either a 365 nm LED (for **NBD‐O1** and **NBD‐O2**) or a 405 nm LED (for all dimers) in toluene, the NBD dimers were successfully converted to their corresponding enriched QC–QC photoisomers (Figure 4). The spectra of **NBD‐P1** and **NBD‐P2** display three apparent isosbestic points, consistent with clean, stepwise photochemical conversion among the three species (NBD‐NBD, NBD‐QC and QC‐QC) without detectable side reactions. The photochemical conversion of **NBD‐O1** and **NBD‐O2** under 405 nm irradiation is shown in Figures S61 and S73. This irradiation wavelength was chosen to highlight the spectral evolution, as irradiation at 365 nm (Figure 4) leads to rapid conversion to the QC–QC isomers. The fast photoconversion under 365 nm irradiation, in particular for **NBD‐O2**, is also evident from the ^1^H‐NMR spectra (Figure S22) that shows only a minor population of the intermediate NBD–QC species. In contrast, upon irradiation at 405 nm, three distinct bands appear in the spectra of **NBD‐O1** and **NBD‐O2** in toluene, accompanied by a gradual decrease in the band at 333 nm and the appearance of two apparent isosbestic points. It appears that full conversion to the QC–QC forms is reached for all dimers except for **NBD‐P2,** where the lowest energy band remains upon prolonged irradiation, suggesting that a photo‐stationary state (PSS) is reached.

Longer wavelength LEDs, namely 430 and 455 nm, were also used for irradiation of **NBD‐P1** and **NBD‐P2** into the tail of the absorption that corresponds to the NBD‐NBD isomer, in an attempt to selectively convert the dimers to their NBD–QC isomers. Selective successive conversion cannot be observed, as previously reported for other similar systems,^[^ ^39^ ^]^ likely due to the electronically coupled nature of the *para*‐ and *ortho*‐linked dimers here. This is also confirmed by ^1^H‐NMR spectroscopy, where irradiation at 455 nm leads to the formation of both NBD–QC and QC–QC. Nevertheless, irradiation at 430 and 455 nm allows for the observation of bands that could be assigned to the QC–NBD isomer. For instance, upon irradiation at 405 nm, the two bands centred at 350 nm for **NBD‐P1** increase in intensity (Figure 4), whereas upon 455 nm irradiation, a decrease in intensity of this band is first observed, accompanied by a slight red‐shift of the band at 360 nm (Figure S29). The longest wavelength absorption band also slightly shifts to shorter wavelengths, as well as decreases in intensity. Similar, but less pronounced spectral changes were also observed for **NBD‐P2** upon conversion with a 455 nm LED (Figure S47). The switching of all NBD dimers was also studied in acetonitrile, showing a similar behaviour as for that in toluene, and the spectra can be found in Section 5 of the Supporting Information.

The switching behaviour of the dimers was also confirmed by ^1^H‐NMR spectroscopy in CDCl_3_. **NBD‐P1** was not studied here as it is reported elsewhere.^[^ ^42^ ^]^ Feringa et al. reported that in situ irradiation at 415 nm in benzene‐*d*_6_ led to complete conversion to the QC–QC form, whilst subsequent irradiation with 365 nm light led to back‐switching, resulting in a PSS composed of all three isomers. Here, we show that **NBD‐P1** and **NBD‐P2** can be photochemically back‐converted upon 365, 340, and 308 nm irradiation to reach a PSS in each case (Figures S14, S31 and S35, S41 and S42, S48–S51 and S57 and S58). **NBD‐O1** and **NBD‐O2** could also be photochemically back‐converted to a PSS under 308 nm irradiation (Figure S64 and S65 and S76 and S77).

For all the studied dimers, upon irradiation with 455 nm (**NBD‐P2**) or 405 nm (**NBD‐O1** and **NBD‐O2**), the percentage of the NBD‐QC form first increases as it is formed from NBD‐NBD, before decreasing as it is consumed and converted to QC–QC (Figure S17, S21, and S24). For **NBD‐P2**, it appears that full conversion is not achieved (Figure S14) as the lowest energy absorption band is still present after irradiation at 405 nm in the UV–vis studies (Figure 4), as mentioned above. Similarly, the PSS was confirmed by ^1^H‐NMR upon irradiation at 455 nm in CDCl_3_ to consist of 75% QC–QC and 25% QC–NBD. Photochemical back‐conversion of **NBD‐P2** to a PSS consisting of 49% NBD‐NBD, 27% NBD‐QC, and 24% QC‐QC was achieved after 15 min of 365 nm irradiation (Figure S17). **NBD‐O1** also reaches a PSS consisting of all three isomers upon conversion in CDCl_3_ with 365 and 405 nm LEDs (Figures S18–S20), though quantitative conversion to the QC–QC form was achieved in CD_2_Cl_2_ after 20 min of irradiation at 405 nm. Irradiation at 365 nm in toluene‐d_8_ (Figure S20b) led to a PSS consisting of 90% QC‐QC and 10% NBD‐QC after 20 min. A complete and rapid conversion of **NBD‐O2** was realised after 5 min of irradiation at 405 nm in both CDCl_3_ (Figure S22) and CD_2_Cl_2_. This highlights the importance of the choice of solvent for a fast and quantitative photoconversion.

### Fluorescence

The emission spectra of all dimers in toluene, acetonitrile, methylcyclohexane (MCH), and 2‐methyltetrahydrofuran (MeTHF) are given in Section 8 of the Supporting Information. The *para* dimers are strongly emissive whilst the *ortho* dimers are weakly emissive (see Quantum Yields section for further discussion). The solvents used were chosen to study the effect of polarity on the optical properties of the dimers. All dimers show a small amount of solvatochromism, with the emission spectra red‐shifting in the order MCH < Toluene ≈ MeTHF < MeCN (Table S3). The emission of **NBD‐P2** is more red‐shifted compared to **NBD‐P1** due to the additional electron density provided by the hexyl chains. The maxima of the lowest energy absorption bands of **NBD‐P1** and **NBD‐P2** are slightly blue‐shifted in MeCN compared to toluene (by 10 and 8 nm for **NBD‐P1** and **NBD‐P2** respectively), whilst the emission spectra are red‐shifted (by 11 nm and 8 nm respectively); there is a more pronounced solvatochromic effect for **NBD‐P1** compared to **NBD‐P2**. The emission profiles are also broader in the more polar MeCN, but the solvatochromic shift is very small, suggesting that the molecules have minimal charge transfer (CT) character. This lack of significant CT character is consistent with density functional theory (DFT) calculations, discussed below. The excitation spectra were also recorded, and these match well with the absorbance spectra of the NBD‐NBD form, indicating that it is indeed the NBD‐NBD form that is emitting in each case. Emission lifetimes are also provided in Table S3 for the most emissive dimers **NBD‐P1** and **NBD‐P2**.

### Quantum Yields

Photoisomerisation quantum yields (Φ_i_) were determined in both toluene and acetonitrile for all the reported molecules in an in‐house automated set‐up^[^ ^45^ ^]^ in the low‐concentration regime (≈ 10^−5^ M, Table 1) at various wavelengths of irradiation. The measured Φ_i_ values span a broad range, from as low as 1.5% to as high as 43% in toluene, with the highest efficiencies observed for the *ortho*‐substituted derivatives (**NBD‐O1** and **NBD‐O2**). Notably, these *ortho*‐derivatives could be efficiently switched using either a 365 or 405 nm LED, with higher Φ_i_ values obtained upon irradiation at the higher‐energy 365 nm wavelength (Table 1). Additional quantum yields upon irradiation at 430 and 455 nm (*para* derivatives only) are provided in the Supporting Information (Table S1).

Quantum yields were calculated at carefully selected analysis wavelengths positioned on the shoulder of the lowest‐energy absorption band to minimise interference from overlapping spectra of the NBD‐NBD, NBD‐QC and QC‐QC species. This challenge is exemplified for **NBD‐P1** (Figures S25 and S26) and **NBD‐P2** (Figures S43 and S44) where analysis at two different wavelengths (for 405 nm irradiation) yielded Φ_i_ values of 7% and 3.5% for **NBD‐P1**, and 1.5% and 0.9% for **NBD‐P2** at 430 and 410 nm, respectively. For these examples, the 430 nm analysis wavelength was selected to ensure that the reported quantum yield values are more representative of the first photoconversion step, as it consistently provided higher Φ_i_ values and reduced the impact of spectral overlap. The calculated absorption spectra for **NBD‐P1** and **NBD‐O1** (Figures S137 and S138) further support this choice, showing that there is no significant spectral overlap of the NBD‐QC isomer on the tail of the NBD‐NBD absorption band. All Φ_i_ values reported here therefore refer to the photoisomerisation event from the NBD–NBD to the NBD‐QC state. Whilst previous work by Feringa et al. suggested doubling the Φ_i_ value to reflect the presence of two photoswitches per molecule, such an approach assumes that both switching steps proceed with identical efficiency. Instead, the values reported here represent the quantum yield of the initial NBD–NBD to NBD–QC conversion, providing a more reliable measure of the first photochemical event, in particular as in these cases, each photoconversion cannot be carried out selectively and successively due to the coupled nature of the switches.

The significantly higher photoisomerisation efficiencies observed for the *ortho*‐derivatives **NBD‐O1** and **NBD‐O2** stand in contrast to the much lower values for the *para*‐substituted systems. This discrepancy stems from the pronounced fluorescence of **NBD‐P1** and **NBD‐P2**, where radiative decay outcompetes non‐radiative pathways such as photoisomerisation or relaxation. To further understand the interplay between fluorescence and photoisomerisation efficiency, the fluorescence quantum yields (Φ_f_) of all dimers were determined in both toluene and acetonitrile (Table 1), whilst additional Φ_f_ values in methylcyclohexane (MCH) and 2‐methyltetrahydrofuran (MeTHF) are provided in the Table S4. The *ortho*‐substituted dimers **NBD‐O1** and **NBD‐O2** exhibit negligible emission with Φ_f_ values below 4%, the highest being for **NBD‐O1** in MeTHF. In contrast, the *para*‐substituted dimers **NBD‐P1** and **NBD‐P2** show markedly higher Φ_f_ values in toluene, but their efficiencies are reduced in acetonitrile. This solvent‐dependent decrease in fluorescence prompted us to investigate whether photoisomerisation could be enhanced under these conditions. Indeed, the Φ_i_ values for all dimers are significantly higher in acetonitrile than in toluene, ranging from 3.5 to 63% indicating that radiative decay is suppressed whilst the non‐radiative photoisomerisation process is favoured. The observed solvent effects are likely the result of a combination of factors, including solvent polarity, specific solvation, and possibly viscosity, which together influence the relative rates of radiative and non‐radiative decay pathways; a systematic investigation would be required to fully disentangle these contributions. Nevertheless, this demonstrates that the quantum yields of photoisomerisation can be tuned by solvent choice,^[^ ^43^ ^]^ with acetonitrile emerging as the preferred medium for these particular systems when higher photoswitching efficiency is desired for energy storage applications.

### DFT Calculations

Density functional theory (DFT) methods were employed to predict the optical properties of all the reported compounds using the wB97X‐D3BJ functional and the aug‐cc‐pVDZ basis set (details given in Section 11 of the Supporting Information). The transition with the highest oscillator strength for the *para*‐derivatives is the S_0_ → S_1_ transition (HOMO → LUMO), consistent with the absorption spectra, where the lowest energy band exhibits higher absorptivity. Meanwhile, for the *ortho*‐derivatives, it is the S_0_ → S_2_ (HOMO → LUMO + 1) transition that has the highest probability. Visualisation of the orbitals involved in these transitions (provided in Section 11 of the Supporting Information) might provide some insight into why fluorescence is more efficient in the *para*‐derivatives whilst photoisomerisation is more favourable for the *ortho*‐substituted dimers. For the former, the LUMO is delocalised across the whole system with significant density on the central phenyl linker, whilst the LUMO + 1 is more localised on the NBD photoswitchable units, which correlates with higher photoisomerisation efficiency. Additionally, the closer spatial proximity of the two NBD units in the *ortho*‐configuration leads to more localised electron density on one side of the molecule, suggesting the possibility of through‐space interactions. This spatial arrangement may promote cooperative switching, where isomerisation of one NBD unit could facilitate isomerisation of the second. This would be consistent with the significantly higher Φ_i_ values for **NBD‐O1** and **NBD‑O2**. These findings could also help to rationalise the increase in the photoisomerisation quantum yield upon irradiation with 365 nm compared to 405 nm for these *ortho*‐dimers, as 365 nm better overlaps with the S_0_ → S_2_ absorption band. This is not the case for the *para*‐dimers as irradiation at 365 nm leads to photochemical back‐conversion to a PSS.

### Kinetics

**Figure 5:**
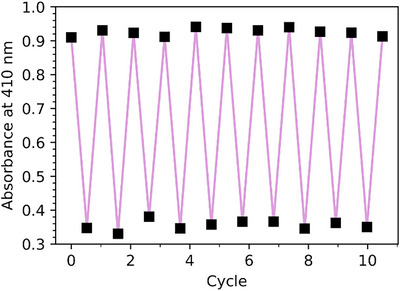
Fatigue study of **NBD‐P2** in toluene with 405 nm irradiation and subsequent thermal back conversion at 55 °C, following the absorbance at 410 nm, where the NBD‐NBD isomer absorbs. No degradation was observed after 10 cycles.

The kinetics of the QC–QC to NBD–NBD thermal back‐reactions were determined at a minimum of four different temperatures, and the half‐lives at 25 °C (*t*_1/2_) were determined from Arrhenius analyses (Table 1, Figures S84–S91). The corresponding activation enthalpy, entropy and activation energy values were determined by Eyring analyses and are provided in Table S2. All dimers display relatively similar half‐lives, ranging from 1.90 to 2.90 h, with the *para*‐derivatives exhibiting slightly shorter half‐lives. For these systems, the individual half‐lives of the NBD–QC and QC–QC forms could not be determined as selective conversion between these states is not possible, unlike in a previous study by Moth–Poulsen et al.^[^ ^39^ ^]^ The relatively short thermal half‐lives here, compared to related NBD dimers without electron‐donating substituents, where half‐lives ranged from 4.33 h up to 48.5 days for the *meta*‐substituted compound **c** (Figure 1),^[^ ^39^ ^]^ are likely a result of the red‐shifted absorption profiles permitted by the donor–acceptor molecular design. The stability of **NBD‐P2** was assessed over 10 cycles of photoirradiation at 405 nm (25 °C) followed by thermal relaxation at 55 °C in toluene (Figure 5). **NBD‐P2** was chosen for this cyclability study as it has the shortest thermal half‐life of the series. The combination of relatively short half‐lives and robust cycling stability highlights the potential of these dimers for short‐timescale heat‐transfer applications, where rapid charging and discharging enable efficient, instantaneous energy release.

### Energy Storage Densities

To determine the energy storage densities (Δ*H*_storage_) of the high‐energy QC–QC isomers, the NBD dimers were irradiated at 405 nm in either CDCl_3_ (**NBD‐P1** and **NBD‐P2**) or CD_2_Cl_2_ (**NBD‐O1** and **NBD‐O2**). The conversion percentages were confirmed by ^1^H‐NMR spectroscopy. For samples that were not fully converted at the time of the differential scanning calorimetry (DSC) measurement, a correction factor was applied, assuming that QC–QC releases twice as much energy as the QC–NBD intermediate state (see Supporting Information Section 7). Solutions containing the converted QC–QC isomers were evaporated under a stream of nitrogen directly into DSC pans for thermal analysis. The DSC traces, along with the ^1^H‐NMR spectra before and after the DSC experiments, are provided in the Supporting Information (see Figures S93–108), and the resulting energy densities are given in Table 2. The measured energy density for **NBD‐P1** is in good agreement with the previously reported value (228.0 kJ kg^‑1^) within experimental error.^[^ ^42^ ^]^

**Table 2:** Measured and calculated heat flow of the QC–QC isomers.

Dimer	Measured Δ*H*_storage_ / kJ mol^−1^	Calculated Δ*H*_storage_ / kJ mol^−1^	Measured Δ*H*_storage_ / kJ kg^−1^	Calculated Δ*H*_storage_ / kJ kg^−1^
**NBD‐P1**	102.5	174.3	246.2	418.5
**NBD‐P2**	97.4	179.3	174.9	322.0
**NBD‐O1**	67.5	165.4	162.1	397.1
**NBD‐O2**	77.8	169.2	139.8	303.9

^‡^The measured values are an average of three DSC runs (see Supporting Information Section 7).

As expected, the methoxy‐substituted dimers exhibit higher energy storage densities than their hexoxy counterparts, primarily due to their lower molecular weights. Interestingly, the energy densities of the *para*‐derivatives are higher than those of the respective *ortho*‐compounds. The calculated Δ*H* values are much higher than the experimentally determined values across the series. This discrepancy is likely due, at least in part, to the relatively short half‐lives of these dimers, which may limit the maximum measurable heat flow during the DSC experiment, despite the application of the correction factor. Nevertheless, the overall trend in the measured data broadly aligns with the theoretical predictions.

### Solar Conversion Efficiencies

**Figure 6:**
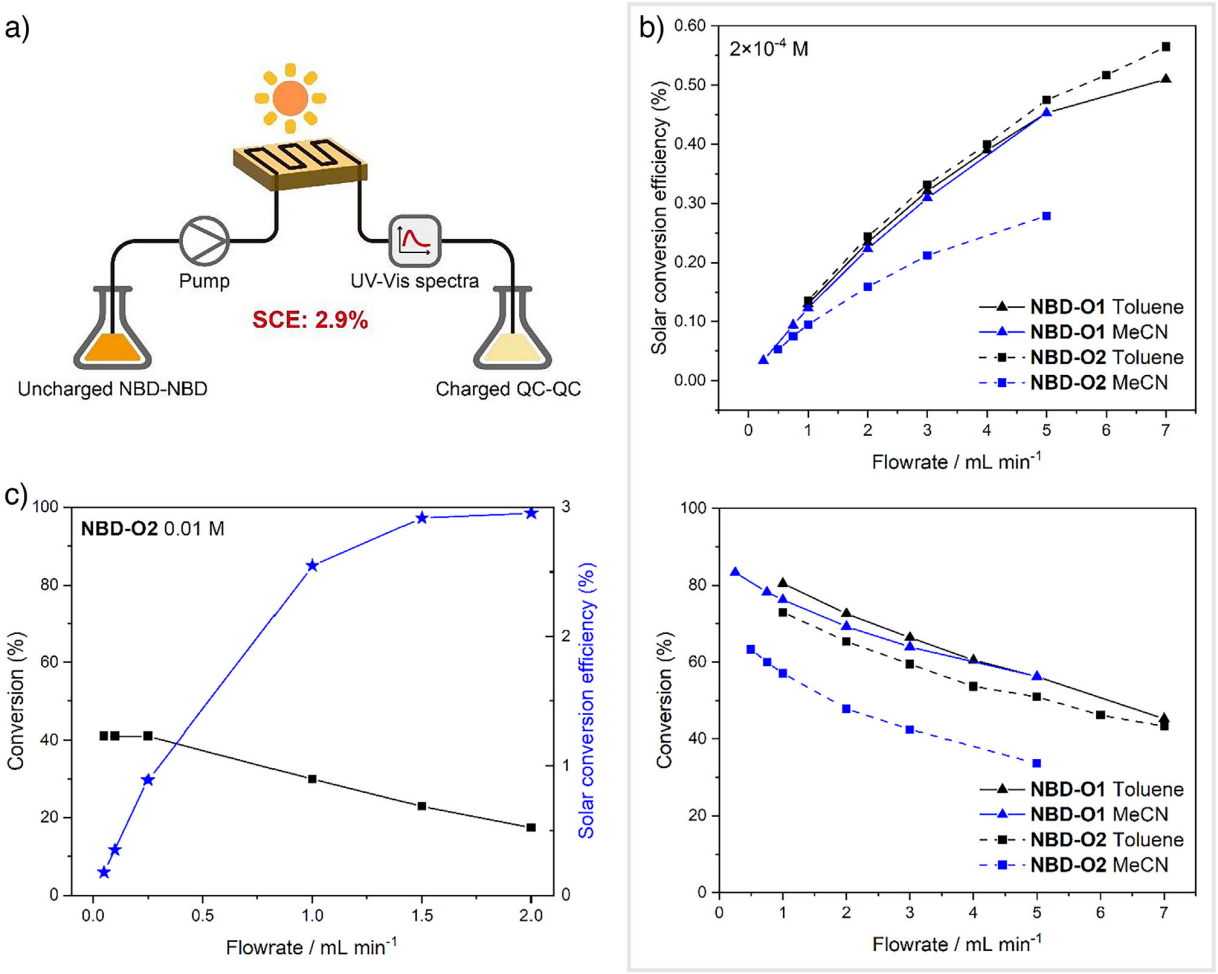
a) Liquid flow‐cell set up for the solar conversion experiments. Experimental solar conversion efficiencies and conversion percentages of the NBD‐NBD to QC‐QC isomers with flowrate for b) **NBD‐O1** and **NBD‐O2** in toluene and acetonitrile at a concentration of 2 × 10^−4^ M and c) **NBD‐O2** in acetonitrile at 0.01 M. Note: Solar device conversion was performed using an infinity PV ISOSun solar simulator (Class A). Previous work^[^ ^28^ ^]^ used a SAN‐EI Electric, XES‐100S1 (class AAA) solar simulator and at a higher concentration of 0.1 M.

To complement the photoconversion measurements and demonstrate that efficient photoswitching also occurs under realistic, broadband visible irradiation conditions relevant to practical applications, the samples were also converted under a solar simulator. To measure the solar conversion efficiency (SCE, *η*_MOST_) of the best performing photoswitches, **NBD‐O1** and **NBD‐O2**, solutions of the dimers in toluene and acetonitrile were passed through a microfluidic chip placed under a solar simulator (Figure 6a, additional details provided in the Supporting Information). At low concentration (≈ 2 × 10^−4^ M), peak SCEs of 0.51% and 0.57% were determined for **NBD‐O1** and **NBD‐O2**, respectively, at flowrates of 7 mL min^−1^ in toluene (Figure 6b). The *para*‐derivatives were also measured, though due to their fluorescence and low photoisomerisation quantum yields, the efficiencies were very low (<0.01%). Aslam et al.^[^ ^29^ ^]^ reported a theoretical upper limit of SCE of 0.16% for a benzothiadiazole‐linked NBD dimer, though, to the best of our knowledge, these are the first experimental solar conversion efficiencies reported for a multichromophoric system.

The SCE upper limits (*η*_limit_) were also predicted and are provided in the Supporting Information. The highest predicted SCE was seen for **NBD‐O2**, the more soluble derivative, in a solar simulator at higher concentration (0.03 M) in acetonitrile, reaching 5.17%. This prompted the synthesis of a larger amount of **NBD‐O2** to carry out further higher concentration experiments. The experimental SCE of **NBD‐O2** in acetonitrile at 0.01 M reaches 2.9% (Figure 6c), higher than the previous record 2.3% efficiency for a monomer NBD.^[^ ^28^ ^]^

Interestingly, for the lower concentration experiments, the measured SCE values for all compounds, with the exception of **NBD‐O2** in acetonitrile, are very similar in both acetonitrile and toluene. This is also seen with the theoretical predictions, as *η*_limit _= 1.42 for **NBD‐O1** in both acetonitrile and toluene at lower concentration. This is contrary to what might be expected given the significantly higher Φ_i_ values observed in acetonitrile relative to toluene. We propose that this discrepancy arises from competing photochemical back‐conversion that is observed for these systems, which arises from the substantial spectral overlap between the NBD‐NBD, NBD‐QC and QC‐QC species. In this context, toluene may help to partially suppress this effect by absorbing more strongly in the UV region, thereby filtering out higher‐energy photons that drive this unwanted back‐reaction. This effect is demonstrated both experimentally and theoretically for **NBD‐O2** where SCEs are higher in toluene than acetonitrile at lower concentration. A related consequence of this competing back‐conversion is the discrepancy between the experimentally measured SCEs and the higher calculated upper limits (Figure S123 and S124), a discrepancy that has also been observed for azobenzene systems.^[^ ^46^ ^]^ This competitive photochemical back‐conversion also reduces the practically achievable photoconversion in the devices; a maximum conversion of 83% was achieved for **NBD‐O1** in acetonitrile at the lowest flow rate for the lower concentration experiment. This effect is exacerbated at higher concentration with a maximum conversion of 41% measured for **NBD‐O2** at the PSS.

To further enhance device performance, future designs could incorporate UV bandpass filters to supress excitation of the reverse reaction.^[^ ^47^ ^]^ Alternatively, a dual‐layer system that combines these photoswitches with another MOST material such as azobenzene, that has been previously shown to act as both a UV filter and an additional energy storage medium,^[^ ^48^ ^]^ could simultaneously broaden the absorption range and improve overall efficiency.

### Catalytic Back Conversion and Macroscopic Heat Release

To demonstrate a practical heat release, an efficient catalyst is required to catalytically trigger the backreaction from QC–QC to NBD‐NBD.^[^ ^49^, ^50^, ^51^ ^]^ Cobalt phthalocyanine absorbed on carbon (CoPc@C) has been successfully shown to trigger the heat release of NBD monomers.^[^ ^52^ ^]^ **NBD‐O2** was chosen as this molecule has a relatively high Φ_i_ in toluene, and it is the most soluble derivative. Although the *para*‐derivatives showed higher energy storage densities, these were not chosen due to their high fluorescence quantum yields and low values of Φ_i_. Moreover, **NBD‐O2** has the highest *t*_1/2_ of all the studied dimers, positioning it as the most practically viable choice for the experiment. **NBD‐O2** was irradiated with 405 nm in CDCl_3_ in an NMR tube (7 × 10^−3^ M) until fully converted. CoPc@C was added and the tube shaken to trigger the back‐reaction. After 10 min, all of the sample was fully converted back to the NBD–NBD parent form (Figure S125), demonstrating efficient catalysis. The catalytic back reaction was also monitored by UV‐vis spectroscopy (Figure S126), where a rate constant for the catalytic back reaction at 25 °C, *k*_cat,_ was determined to be 1.68 × 10^−3^ s^−1^, two orders of magnitude greater than the thermal back reaction at the same temperature (*k*_t_ = 6.64 × 10^‑5^ s^−1^) demonstrating efficient catalysis for the QC–QC to NBD–NBD process.

To perform the macroscopic heat release experiment to assess the potential of **NBD‐O2** for MOST applications, the sample was first photoconverted in a flow UV reactor. Full conversion of a 0.1 M solution of **NBD‐O2** in toluene was achieved under 365 nm irradiation with a residence time of 15 min (experimental details in Section 10 of the Supporting Information). At higher concentrations, complete conversion could not be reached: for a 0.5 M solution, the PSS comprised 45% NBD‐NBD, 38% NBD‐QC and 17% QC‐QC. This limitation is attributed to photochemical back‐conversion that is exacerbated at higher concentrations. Despite the incomplete conversion, the 0.5 M solution of **NBD‐O2** was used to demonstrate a macroscopic heat release. Using a vacuum setup and CoPc@C as the catalyst (Figure S131), a heat release of 5.78 °C was recorded (Figure S132). Accounting for the incomplete conversion and post‐experiment state, the effective concentration of reactive photoisomer species was estimated to be 0.1 M. The heat release measured is consistent with previous reports for an NBD monomer at the same concentration.^[^ ^52^ ^]^ Based on the theoretical Δ*H*_storage_, a ΔT of 12.6 °C would be expected for a 0.1 M fully converted **NBD‐O2** solution in toluene. Whilst the observed heat release does not yet reach the theoretical maximum, this first demonstration highlights both the challenges and the promise of using dimer systems for MOST applications.

## Conclusion

A series of *ortho*‐ and *para*‐substituted NBD dimers featuring methoxy‐, hexoxy‐, and cyano‐substitutents were synthesised and evaluated for their MOST energy storage properties. The dimer systems show red‐shifted absorption profiles with improved overlap with the solar spectrum, owing to their donor‐acceptor design, with absorption onsets extending into the visible region of the spectrum (448–488 nm). The dimers could all be converted to their corresponding metastable isomers NBD–QC and QC–QC upon irradiation. Photoswitching behaviour was investigated in both toluene and acetonitrile, revealing that the photoisomerisation efficiency is higher in acetonitrile, with photoisomerisation quantum yield (Φ_i_) values reaching up to 63%. The *para*‐derivatives demonstrated significantly low Φ_i_ values due to competitive fluorescence, with fluorescence quantum yields of up to 56%, with more efficient fluorescence in toluene than in acetonitrile. This highlights the interplay between photoisomerisation and photoluminescence that can be tuned through appropriate solvent selection and molecular design, depending upon the desired application, opening up possibilities for tailoring these systems towards specific functions through rational molecular design and environmental control.

Thermal half‐lives of the photoisomers ranged from 1.9 to 2.9 h, offering short‐term energy storage windows for rapid cycling. The performance of the dimers was further demonstrated in a liquid‐flow chip device to measure solar conversion efficiencies (SCEs), with peak experimental SCEs of 0.51% and 0.57% for **NBD‐O1** and **NBD‐O2**, respectively, in toluene at 2 × 10^−4^ M, and a record SCE of 2.9% for the more soluble **NBD‐O2** at elevated concentration (0.01 M) in acetonitrile with a corresponding theoretical upper limit of 5.17%. The disparity between theoretical and experimental efficiencies is primarily attributed to photochemical back‐conversion, a key factor that must be addressed in the design of future systems. Additionally, the catalytic back‐conversion of the best‐performing photoswitch was demonstrated using a CoPc@C catalyst, and a macroscopic heat release experiment of a flow‐converted sample yielded a temperature increase of 5.78 °C for an effective concentration of 0.1 M of **NBD‐O2**, comparable to that of monomeric systems. This study highlights both the potential and inherent challenges of dimer‐based systems, providing valuable insights into the limitations of spectral overlap and photochemical back‐conversion, as well as competitive fluorescence, that will help inform the development of future, more efficient devices.

## Conflict of Interests

The authors declare no conflict of interest.

## Supporting information



Supporting Information

## Data Availability

The data that support the findings of this study are available in the Supporting Information of this article.
