# Supplementary material for: Modulation of Photoluminescence and Solar Thermal Energy Storage in Norbornadiene–Quadricyclane Dimers
Source: Angew Chem Int Ed Engl. 2025 Nov 4;65(1):e16629. doi: 10.1002/anie.202516629 (PMC12759240; doi:10.1002/anie.202516629)
Supplement: Supplementary file 1 — Supporting Information [file ANIE-65-e16629-s001.docx]

Supporting Information

**Modulation of Photoluminescence and Solar Thermal Energy Storage in Norbornadiene-Quadricyclane Dimers**

Rebecca J. Salthouse, Jacob L. Elholm, Irene Cortellazzi, Helen Hölzel, Pedro Ferreira, Lorette Fernandez, Marc K. Etherington and Kasper Moth-Poulsen

**Table of contents**

[1 General experimental S2](#_Toc211350528)

[2 Experimental S3](#_Toc211350529)

[3 NMR spectra S14](#_Toc211350530)

[4 NMR irradiation studies S21](#_Toc211350531)

[5 Quantum yield determination and photoswitching behaviour S30](#_Toc211350532)

[6 Kinetics S61](#_Toc211350533)

[7 Energy density (Differential Scanning Calorimetry) S67](#_Toc211350534)

[8 Fluorescence S79](#_Toc211350535)

[9 Solar simulator measurements S85](#_Toc211350536)

[10 Catalytic back conversion and macroscopic heat release S92](#_Toc211350537)

[11 Computational details S101](#_Toc211350538)

[12 Stern-Volmer plots S122](#_Toc211350539)

[13 References S126](#_Toc211350540)

# 1 General experimental

Reagents, including dry solvents, were obtained from commercial sources and used without further purification unless stated otherwise. All solvents used in preparative work were at least of Analar grade. For procedures involving dry solvent, glassware was oven-dried at 110 °C prior to use. Reactions requiring an inert atmosphere were carried out using Schlenk-line techniques under an atmosphere of nitrogen. Flash column chromatography was carried out on a Buchi Pure C-815 Flash. Thin-layer chromatography was carried out using silica plates (MerckArt 5554) and visualized by UV radiation at 254 and/or 365 nm. NMR spectra were recorded on a Bruker Ascend-400 (400 MHz) spectrometer at 298 K. Chemical shifts (δ) are in ppm, referenced to residual protio solvent resonances (CDCl_3_: ^1^H δ = 7.26 ppm; ^13^C δ = 77.16 ppm). Electrospray ionization mass spectral data (positive and negative modes) were obtained on an SQD mass spectrometer interfaced with an Acquity UPLC system with acetonitrile as the carrier solvent. Spectra acquired using an atmospheric solids atomization probe (ASAP) were recorded on a Waters Xevo QToF mass spectrometer. Differential Scanning Calorimetry (DSC) was performed on a Q100 thermal analyser from TA Instruments. The device has been calibrated using the melting point of indium (T_fus_ = 429.75 K and Δ_fus_H = 28.45 J g^-1^) and the samples were weighed using a microbalance sensitive to 0.01 mg and sealed in aluminium pans. The heating and cooling runs were performed at a rate of 20 °C min^-1^ for the heat release measurements. The melting points of the NBDs were also determined by DSC with a heating/cooling rate of 5 °C min^-1^; the onset temperature of the peak is reported as the melting point.The solution samples for fluorescence measurements were prepared by dissolving the compound in a stock toluene solution at either 1 mg mL^-1^ or 0.1 mg mL^-1^ (depending on solubility) before dilution to spectroscopic concentrations of 20 µM. The absorbance measurements were performed on a Shimadzu SolidSpec 3700 using the relevant solvent as a blank. The steady-state photoluminescence emission and excitation measurements were performed on a HORIBA Fluorolog-QM. Photoluminescence quantum yields (PLQYs) were obtained via the absolute method using an integrating sphere (HORIBA FM-Sphere) in conjunction with the HORIBA Fluorolog-QM, with neutral density filters to correct for high intensity excitation. The PLQYs were calculated using the mathematical formula included within the HORIBA FelixFl software. Finally, the lifetimes were obtained via time-correlated single photon counting (TCSPC) using a 350 nm Delta Diode as the excitation source and the TCSPC add-on that is provided for the Fluorolog-QM.

# 2 Experimental

The synthesis of the *ortho*-NBD dimers is shown in **Scheme S1**, whilst that of the *para*-NBD dimers is given in **Scheme S2**. The first step of the synthesis involves alkylation to prepare the methoxy (**1**) or hexoxy chains (**2**), followed by iodination that is *para*-directed owing to the donating alkoxy groups to give the intermediates **3** and **4** in excellent yields (82 and 74% respectively). A Sonogashira coupling was employed to introduce the trimethylsilylacetylene groups, followed by deprotection using potassium carbonate. These acetylene precursors **7** and **8** are reacted with the key intermediate 3-bromo-2-cyanonorbornadiene (synthesised from 2,3-dibromonorbornadiene as reported elsewhere)^1^ through a Sonogashira cross-coupling to give the dimers **NBD-O1** and **NBD-O2** in good yields (43% and 53% respectively). The synthesis of the *para*-dimers involved fewer steps, not requiring iodination, as 1,4-diiodo-2,5-dihydroxybenzene and 1,4-diiodo-2,5-dimethoxybenzene are commercially available.

***Scheme S1.*** *Synthesis of the ortho-substituted dimers* ***NBD-O1*** *and* ***NBD-O2****.*

***Scheme S2.*** *Synthesis of the para-substituted dimers* ***NBD-P1*** *and* ***NBD-P2****.*

***1,2-dimethoxybenzene (1)***

To a 250 mL round-bottom flask under an atmosphere of nitrogen were added catechol (2.00 g, 18.2 mmol), methyl iodide (7.73 g, 54.5 mmol), and K_2_CO_3_ (10.0 g, 72.6 mmol) with dry DMF (60 mL). The solution was degassed by N_2_ purging and stirred at RT for 16 h. The crude mixture was extracted with CH_2_Cl_2_ (3 x 50 mL) and the combined organics washed with water (2 x 20 mL) followed by 1 M HCl (1 x 20 mL). The organic phase was dried over MgSO_4_, filtered and the solvent removed under reduced pressure to give the product as an orange liquid without the need for further purification (1.59 g, 63%); ^1^H-NMR (400 MHz, CDCl_3_): 𝛿 6.90-6.84 (m, 4H, H^1^ and H^2^), 3.85 (s, 6H, H^3^). The data obtained were in good agreement with the literature.^2^

***1,2-dihexyloxybenzene (2)***

To a 500 mL round-bottom flask were added 1,2-dihydroxybenzene (5.00 g, 45.4 mmol), 1-bromohexane (30.0 g, 181 mmol) and K_2_CO_3_ (25.1 g, 182 mmol) in CH_3_CN (50 mL). The resulting grey suspension was stirred and heated to 90°C for 22 h. The mixture was then filtered and the solvents evaporated under reduced pressure to give the product as a dark yellow liquid that was used in the subsequent step without further purification; ^1^H-NMR (400 MHz, CDCl_3_): 𝛿 6.89 (s, 4H, H^1^ and H^2^), 4.00 (t, *J =* 6.6 Hz, 4H, H^3^), 1.90-1.78 (m, 4H, H^4^), 1.52-1.40 (m, 4H, H^5^), 1.37-1.30 (m, 8H, H^6^ and H^7^), 0.93-0.89 (m, 6H, H^8^). The data obtained were in good agreement with the literature.^2^

***1,2-diiodo-3,4-dimethoxybenzene (3)***

H_5_IO_6_ (1.05 g, 4.62 mmol), I_2_ (2.34 g, 9.23 mmol), and 1,2-dimethoxybenzene (1.59 g, 11.5 mmol) were added with EtOH (15 mL) to a 100 mL 3-necked round-bottom flask under an atmosphere of nitrogen. The mixture was heated to 70°C with stirring for 19 h, before quenching with the addition of Na_2_S_2_O_5_ (20 mL, 1 M). The mixture was then extracted with CH_2_Cl_2_ (50 mL) and washed with deionised water (2 x 20 mL). The organic phase was dried over MgSO_4_, filtered and the solvent evaporated under reduced pressure to give the product as a light orange solid without the need for further purification (3.67 g, 82%); ^1^H-NMR (400 MHz, CDCl_3_): 𝛿 7.24 (s, 2H, H^1^), 3.84 (s, 6H, H^2^). The data obtained were in good agreement with the literature.^2^

***4,5-dihexyloxy-1,2-diiodobenzene (4)***

To a 250 mL 3 necked round-bottom flask under nitrogen was added 1,2-dihexyloxybenzene (7.50 g, 26.9 mmol), H_5_IO_6_ (2.49 g, 10.2 mmol), I_2_ (5.63 g, 22.2 mmol) and ethanol (60 mL). The resulting suspension was heated to 70°C for 16 h in an inert atmosphere. The reaction mixture was cooled to room temperature and quenched by the addition of saturated aqueous Na_2_S_2_O_5_ solution (60 mL). The mixture was then extracted with CH_2_Cl_2_ (100 mL) and washed with deionised water (2 x 40 mL). The organic phase was dried over MgSO_4_, filtered and the solvent evaporated to give the product as an orange liquid (10.6 g, 74%); ^1^H-NMR (400 MHz, CDCl_3_): 𝛿 7.25 (s, 2H, H^1^), 3.92 (t, *J* = 6.6 Hz, 4H, H^2^), 1.89-1.75 (m, 4H, H^3^), 1.47-1.39 (m, 4H, H^4^), 1.34-1.29 (m, 8H, H^5^ and H^6^), 0.91-0.88 (m, 6H, H^7^). The data obtained were in good agreement with the literature.^2^

***1,4-dihexyloxy-2,5-diiodobenzene (9)***

To a 50 mL round-bottom flask were added 1,4-diiodo-2,5-dihydroxybenzene (1.00 g, 2.76 mmol), 1-bromohexane (1.82 g, 11.1 mmol) and K_2_CO_3_ (1.53 g, 11.1 mmol) with CH_3_CN (10 mL). The brown mixture was stirred and heated to 90°C for 19 h. The resulting mixture was then filtered and the solvent evaporated under reduced pressure. The crude mixture was purified by flash column chromatography (SiO_2_, hexane: ethyl acetate gradient) to obtain the pure product as transparent crystals (651 mg, 45%); ^1^H-NMR (400 MHz, CDCl_3_): 𝛿 7.18 (s, 2H, H^1^), 3.93 (t, *J* = 6.6 Hz, 4H, H^2^), 1.84-1.77 (m, 4H, H^3^), 1.52-1.47 (m, 4H, H^4^), 1.37-1.33 (m, 8H, H^5^ and H^6^), 0.93-0.90 (m, 6H, H^7^). The data obtained were in good agreement with the literature.^3^

**General Sonogashira procedure for acetylation**

To a 3-necked round-bottom flask were added the aryl bromide (1 equiv.) in Et_3_N and THF (1:1). The solution was degassed for 1 hour by nitrogen purging. CuI (10 mol%), Pd(PPh_3_)_2_Cl_2_ (10 mol%) and trimethylsilylacetylene (2.2 equiv.) were added and the reaction mixture stirred at 50°C for 24 h. The mixture was cooled to room temperature, filtered over silica and washed with CH_2_Cl_2_. The solvent was evaporated under reduced pressure and the crude product purified by flash column chromatography (SiO_2_, hexane: ethyl acetate gradient).

***1,2-dimethoxy-4,5-bis[2-(trimethylsilyl)ethynyl]benzene (5)***

The general Sonogashira procedure for acetylation was followed with 1,2-diiodo-3,4-dimethoxybenzene (1.34 g, 3.44 mmol), THF (15 mL) and NEt_3_ (15 mL) to give the product as an orange solid (862 mg, 76%); ^1^H-NMR (400 MHz, CDCl_3_): 𝛿 6.92 (s, 2H, H^1^), 3.88 (s, 6H, H^2^), 0.27 (s, 18H, H^3^). The data obtained were in good agreement with the literature.^4^

***1,2-dihexyloxy-4,5-bis[2-(trimethylsilyl)ethynyl]benzene (6)***

The general Sonogashira procedure for acetylation was followed with 4,5-dihexyloxy-1,2-diiodobenzene (1.00 g, 1.86 mmol), THF (10 mL) and NEt_3_ (10 mL) to give the product as a dark brown oil (612 mg, 70%); ^1^H-NMR (400 MHz, CDCl_3_): 𝛿 6.90 (s, 2H, H^1^), 3.97 (t, *J* = 6.6 Hz, 4H, H^2^), 1.86-1.77 (m, 4H, H^3^), 1.48-1.40 (m, 4H, H^4^), 1.36-1.29 (m, 8H, H^5^ and H^6^), 0.92-0.89 (m, 6H, H^7^), 0.27 (s, 18H, H^8^). The data obtained were in good agreement with the literature.^5^

***1,4-dimethoxy-2,5-bis[2-(trimethylsilyl)ethynyl]benzene (10)***

The general Sonogashira procedure for acetylation was followed with 1,4-diiodo-2,5-dimethoxybenzene (1.00 g, 2.56 mmol), THF (10 mL) and NEt_3_ (10 mL) to give the product as a pale yellow solid (327 mg, 39%); ^1^H-NMR (400 MHz, CDCl_3_): 𝛿 6.92 (s, 2H, H^1^), 3.84 (s, 6H, H^2^), 0.27 (s, 18H, H^3^). The data obtained were in good agreement with the literature.^6^

***1,4-dihexyloxy-2,5-bis[2-(trimethylsilyl)ethynyl]benzene (11)***

The general Sonogashira procedure for acetylation was followed with 1,4-dihexyloxy-2,5-diiodobenzene (651 mg, 3.44 mmol), THF (7 mL) and NEt_3_ (7 mL) to give the product as a yellow solid (568 mg, 98%); ^1^H-NMR (400 MHz, CDCl_3_): 𝛿 6.89 (s, 2H, H^1^), 3.95 (t, *J =* 6.4 Hz, 4H, H^2^), 1.81-1.75 (m, 4H, H^3^), 1.53-1.47 (m, 4H, H^4^), 1.35-1.31 (m, 8H, H^5^ and H^6^), 0.93-0.91 (m, 6H, H^7^), 0.26-0.25 (m, 18H, H^8^). The data obtained were in good agreement with the literature.^3^

**General procedure for TMS deprotection**

K_2_CO_3_ (2.5 equiv.) was added to a stirred solution of the desired TMS-protected acetylene (1 equiv.) in MeOH under an inert atmosphere. The resulting suspension was stirred under nitrogen at room temperature for 1 h. The mixture was filtered on celite, and the solvent evaporated under reduced pressure to give the product without the need for further purification.

***1,2-diethynyl-4,5-dimethoxybenzene (7)***

The general procedure for deprotection was followed with 1,2-dimethoxy-4,5-bis[2-(trimethylsilyl)ethynyl]benzene (177 mg, 0.54 mmol) to give the product as a brown solid (95 mg, 95%); ^1^H-NMR (400 MHz, CDCl_3_): 𝛿 6.97 (s, 2H, H^1^), 3.89 (s, 6H, H^2^), 3.28 (s, 2H, H^3^). The data obtained were in good agreement with the literature.^4^

***1,2-diethynyl-4,5-dihexyloxybenzene (8)***

The general procedure for deprotection was followed with 1,2-dihexyloxy-4,5-bis[2-(trimethylsilyl)ethynyl]benzene (280 mg, 0.59 mmol) to give the product as a brown solid (139 mg, 72%); ^1^H-NMR (400 MHz, CDCl_3_): 𝛿 6.95 (s, 2H, H^1^), 3.99 (t, *J* = 6.6 Hz, 4H, H^2^), 3.50 (s, 2H, H^8^), 1.85-1.78 (m, 4H, H^3^), 1.48-1.43 (m, 4H, H^4^), 1.36-1.32 (m, 8H, H^5^ and H^6^), 0.92-0.89 (m, 6H, H^7^). The data obtained were in good agreement with the literature.^5^

***1,4-diethynyl-2,5-dimethoxybenzene (12)***

The general procedure for deprotection was followed with 1,4-dimethoxy-2,5-bis[2-(trimethylsilyl)ethynyl]benzene (195 mg, 0.59 mmol) to give the product as a brown solid (105 mg, 96%); ^1^H-NMR (400 MHz, CDCl_3_): 𝛿 6.99 (s, 2H, H^1^), 3.87 (s, 6H, H^2^), 3.40 (s, 2H, H^3^). The data obtained were in good agreement with the literature.^6^

***1,4-diethynyl-2,5-dihexyloxybenzene (13)***

The general procedure for deprotection was followed with 1,4-dihexyloxy-2,5-bis[2-(trimethylsilyl)ethynyl]benzene (205 mg, 0.44 mmol) to give the product as a brown solid (125 mg, 88%); ^1^H-NMR (400 MHz, CDCl_3_): 𝛿 6.96 (s, 2H, H^1^), 3.98 (t, *J =* 6.6 Hz, 4H, H^2^), 3.33 (s, 2H, H^8^), 1.84-1.77 (m, 4H, H^3^), 1.51-1.44 (m, 4H, H^4^), 1.38-1.32 (m, 8H, H^5^ and H^6^), 0.92-0.89 (m, 6H, H^7^). The data obtained were in good agreement with the literature.^3^

**General Sonogashira procedure for coupling to NBD**

To a 2 necked round-bottom flask were added the deprotected acetylene (1 equiv.) and 3-bromo-2-cyanonorbornadiene (2.3 equiv.) in dry THF and Et_3_N under an inert atmosphere. The solution was degassed by nitrogen bubbling before the addition of CuI (10 mol%) and Pd(PPh_3_)_2_Cl_2_ (10 mol%). The resulting suspension was stirred at room temperature under an inert atmosphere for 3 days before filtration over celite. The solvent was removed under reduced pressure and the crude purified by flash column chromatography (SiO_2_, hexane: ethyl acetate).

**NBD-P1**

This dimer was synthesised following the general Sonogashira procedure with 1,4-diethynyl-2,5-dimethoxybenzene (105 mg, 0.57 mmol), THF (8 mL) and NEt_3_ (3 mL) to give the product as a bright yellow solid (78 mg, 33%); ^1^H-NMR (400 MHz, CDCl_3_): 𝛿 6.97 (s, 2H, H^7^), 6.90-6.86 (m, 4H, H^1^ and H^2^), 3.91-3.85 (m, 10H, H^3^, H^4^ and H^8^), 2.35-2.33 (m, 2H, H^5^ or H^6^), 2.25-2.23 (m, 2H, H^5^ or H^6^); HRMS (ES^+^): *m/z* 417.1588 [M + H]^+^, calcd for 417.1603 [C_28_H_21_N_2_O_2_]. The data obtained were in good agreement with the literature.^7^

**NBD-P2**

This dimer was synthesised following the general Sonogashira procedure with 1,4-diethynyl-2,5-dihexyloxybenzene (125 mg, 0.38 mmol), THF (8 mL) and NEt_3_ (3 mL) to give the product as a bright orange solid (152 mg, 71%); m.p. 114°C; ^1^H-NMR (400 MHz, CDCl_3_): 𝛿 6.95 (s, 2H, H^7^), 6.90-6.84 (m, 4H, H^1^ and H^2^), 4.01 (t, *J* = 6.4 Hz, 4H, H^8^), 3.91-3.84 (m, 4H, H^3^ and H^4^), 2.34-2.32 (m, 2H, H^5^ or H^6^), 2.24-2.22 (m, 2H, H^5^ or H^6^), 1.88-1.81 (m, 4H, H^9^), 1.52-1.48 (m, 4H, H^10^), 1.38-1.34 (m, 8H, H^11^ and H^12^), 0.93-0.89 (m, 6H, H^13^); ^13^C-NMR (101 MHz, CDCl_3_): 𝛿 153.7, 153.6, 142.1, 141.2, 128.1, 116.6, 116.3, 114.0, 104.2, 89.0, 72.7, 69.6, 57.3, 54.2, 31.5, 29.1, 25.7, 22.6, 14.0; HRMS (ASAP^+^): *m/z* 557.3168 [M + H]^+^, calcd for 557.3168 [C_38_H_41_N_2_O_2_].

**NBD-O1**

This dimer was synthesised following the general Sonogashira procedure with 1,2-diethynyl-4,5-dimethoxybenzene (95 mg, 0.51 mmol), THF (4 mL) and NEt_3_ (3 mL) to give the product as a dark yellow solid (91 mg, 43%); ^1^H NMR (400 MHz, CDCl_3_) δ 6.99 (s, 2H, H^7^), 6.89 (t, *J =* 2.0 Hz, 4H, H^1^ and H^2^), 3.98 – 3.87 (m, 10H, H^3^, H^4^ and H^8^), 2.42 – 2.32 (m, 2H, H^5^ or H^6^), 2.24 (dt, *J =* 7.1 Hz, 1.6, 2H, H^5^ or H^6^); ^13^C-NMR (101 MHz, CDCl_3_): 𝛿 153.8, 150.1, 142.0, 141.4, 128.1, 117.8, 116.5, 114.5, 106.1, 86.5, 72.8, 57.5, 56.2, 54.2; HRMS (ES^+^): *m/z* 417.1588 [M + H]^+^, calcd for 417.1603 [C_28_H_21_N_2_O_2_].

**NBD-O2**

This dimer was synthesised following the general Sonogashira procedure with 1,2-diethynyl-4,5-dihexyloxybenzene (139 mg, 0.43 mmol), THF (8 mL) and NEt_3_ (3 mL) to give the product as a brown oily solid (126 mg, 53%); ^1^H-NMR (400 MHz, CDCl_3_): 𝛿 6.97 (s, 2H, H^7^), 6.88 (t, *J* = 2.0 Hz, 4H, H^1^ and H^2^), 4.02 (t, *J =* 6.6 Hz, 4H, H^8^), 3.92-3.91 (m, 4H, H^3^ and H^4^), 2.37-2.34 (m, 2H, H^5^ or H^6^), 2.24-2.21 (m, 2H, H^5^ or H^6^), 1.87-1.80 (m, 4H, H^9^), 1.50-1.44 (m, 4H, H^10^), 1.37-1.34 (m, 8H, H^11^ and H^12^), 0.93-0.89 (m, 6H, H^13^); ^13^C-NMR (101 MHz, CDCl_3_): 𝛿 153.9, 150.2, 142.0, 141.4, 127.7, 117.5, 116.5, 116.0, 106.4, 86.3, 72.7, 69.3, 57.5, 54.2, 31.5, 29.0, 25.6, 22.6, 14.0; HRMS (ASAP^+^): *m/z* 557.3170 [M + H]^+^, calcd for 557.3168 [C_38_H_41_N_2_O_2_].

# 3 NMR spectra


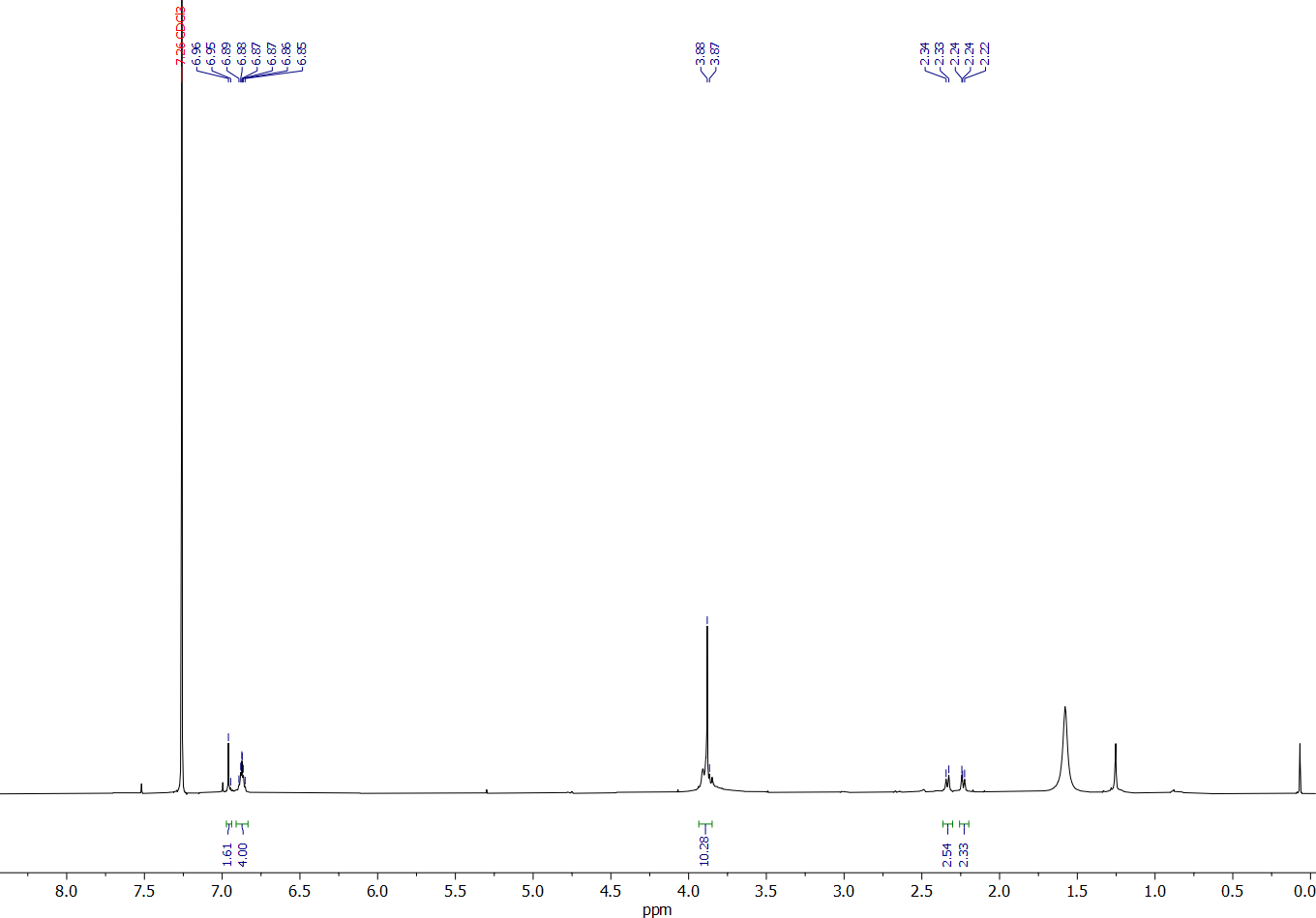


**Figure S1.** ^1^H-NMR spectrum of **NBD-P1** in CDCl_3_ at 400 MHz.


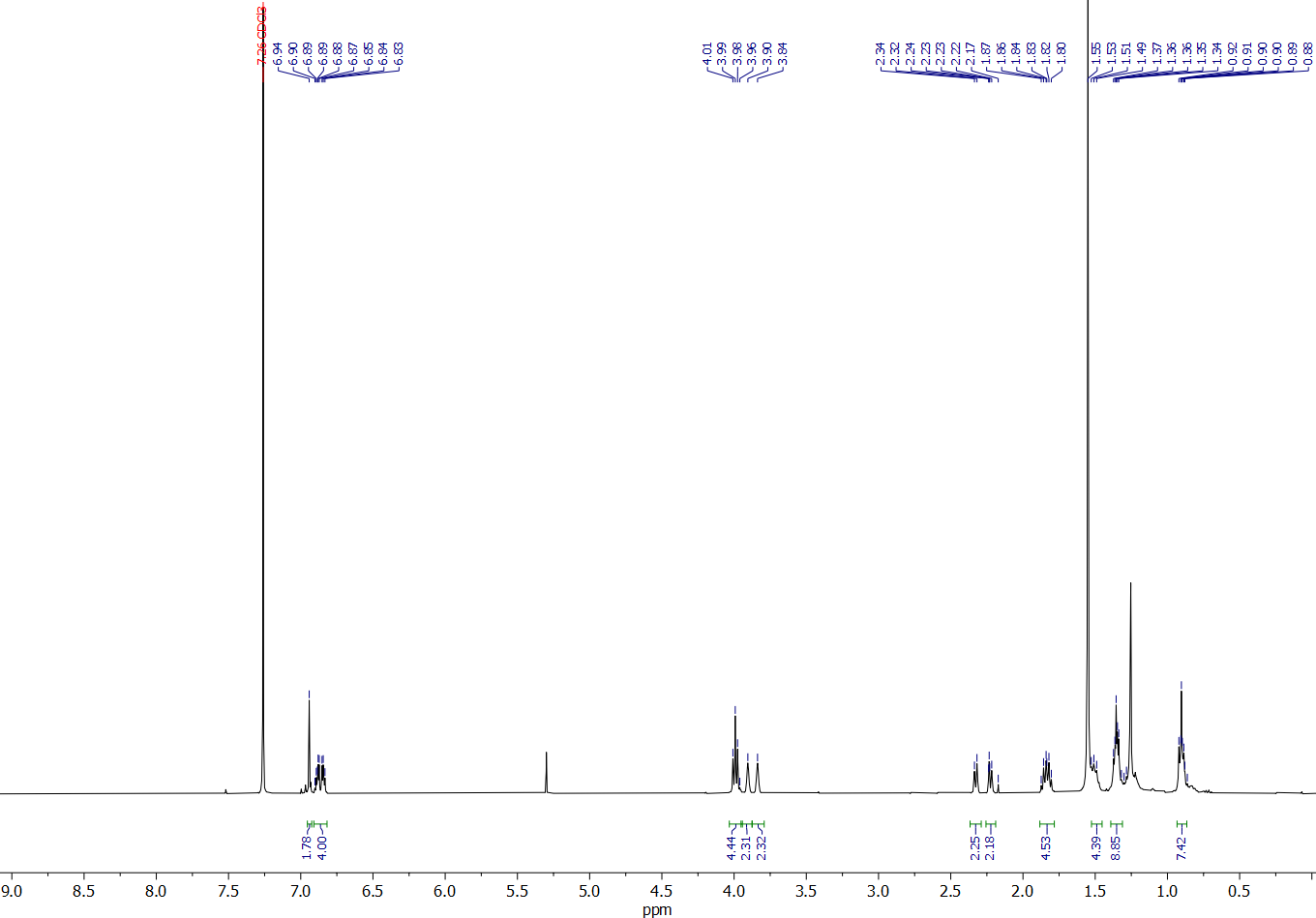


**Figure S2.** ^1^H-NMR spectrum of **NBD-P2** in CDCl_3_ at 400 MHz.

**
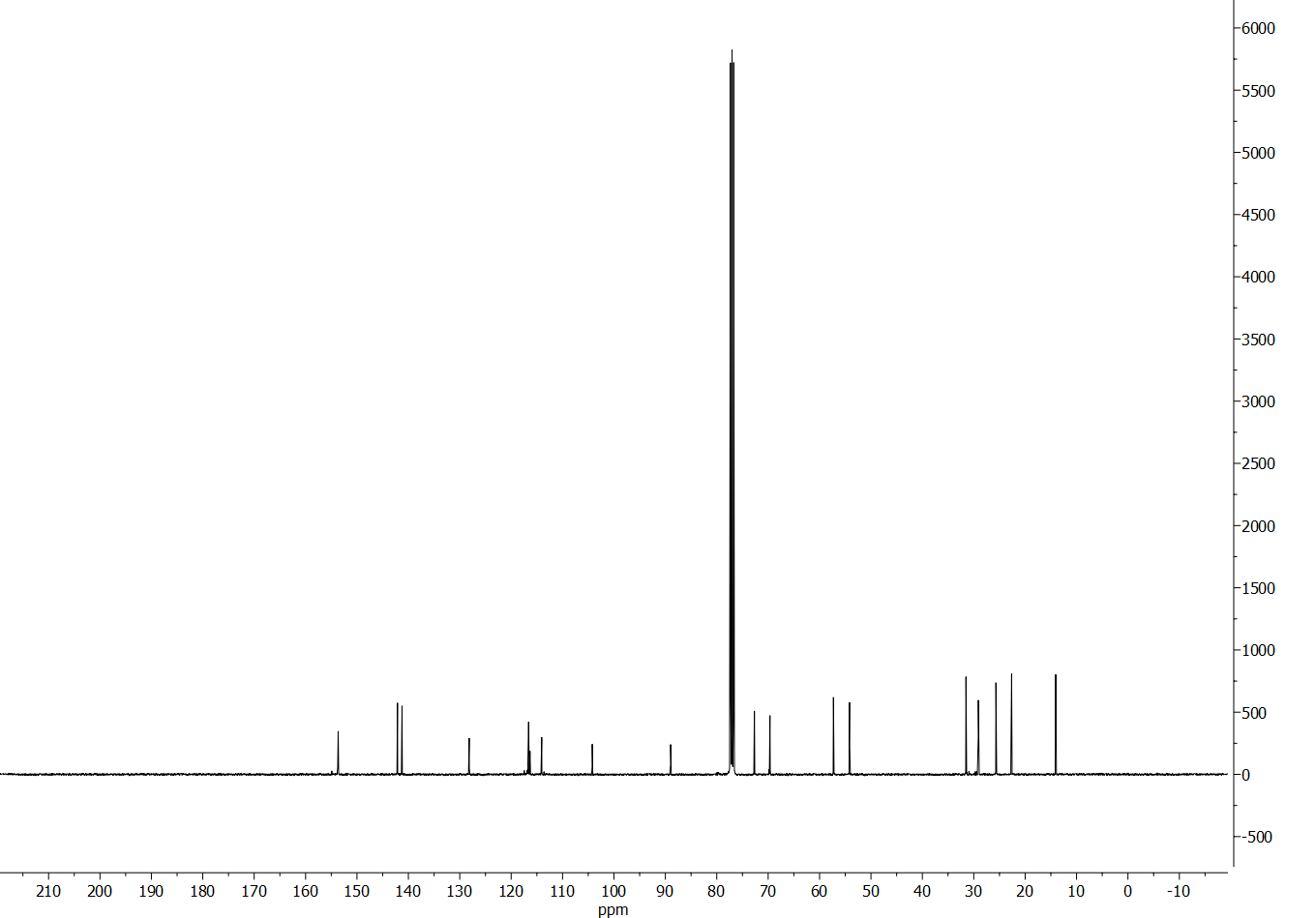
Figure S3.** ^13^C-NMR spectrum of **NBD-P2** in CDCl_3_ at 101 MHz.

**
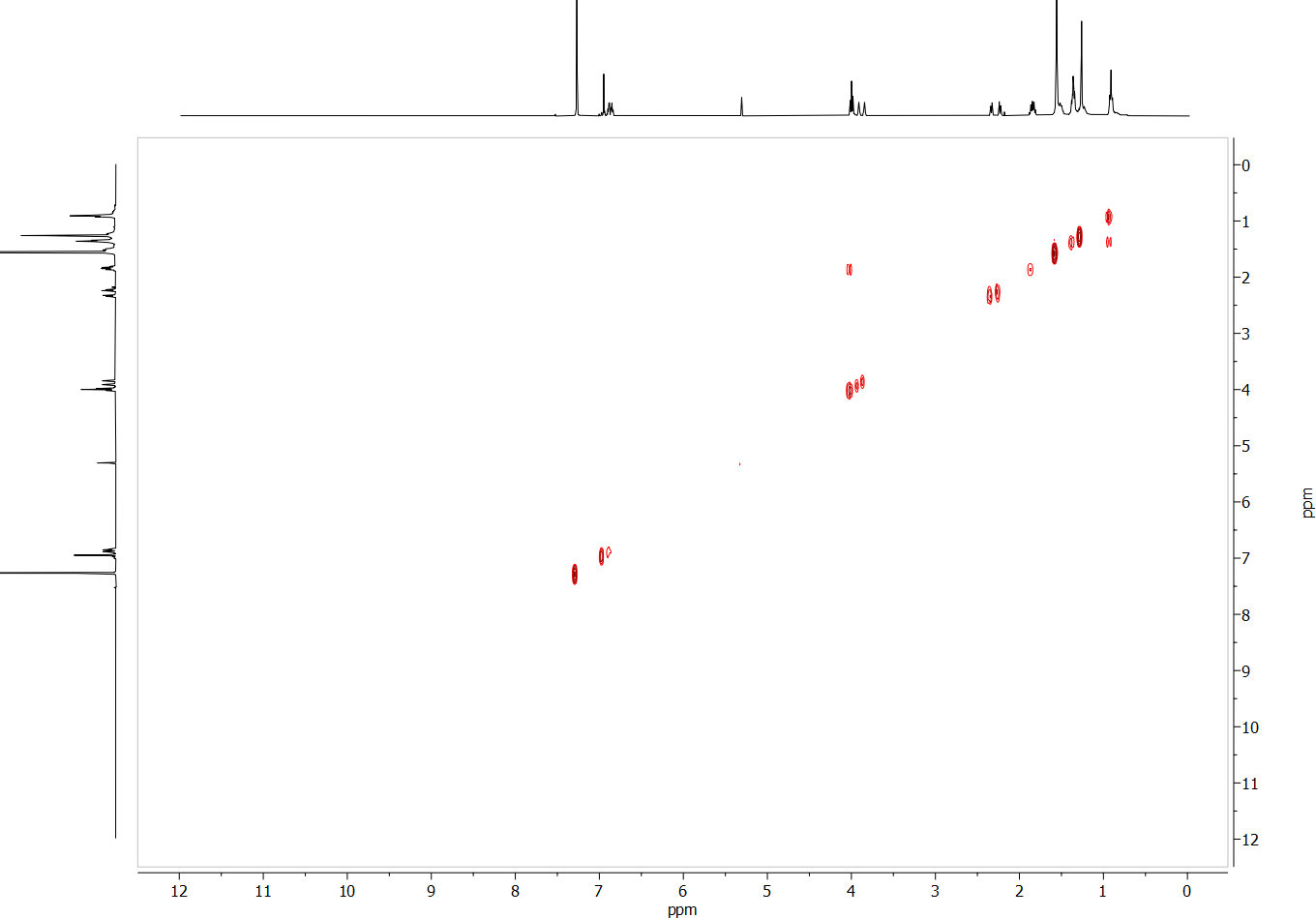
**

**Figure S4.** COSY NMR spectrum of **NBD-P2** in CDCl_3_.


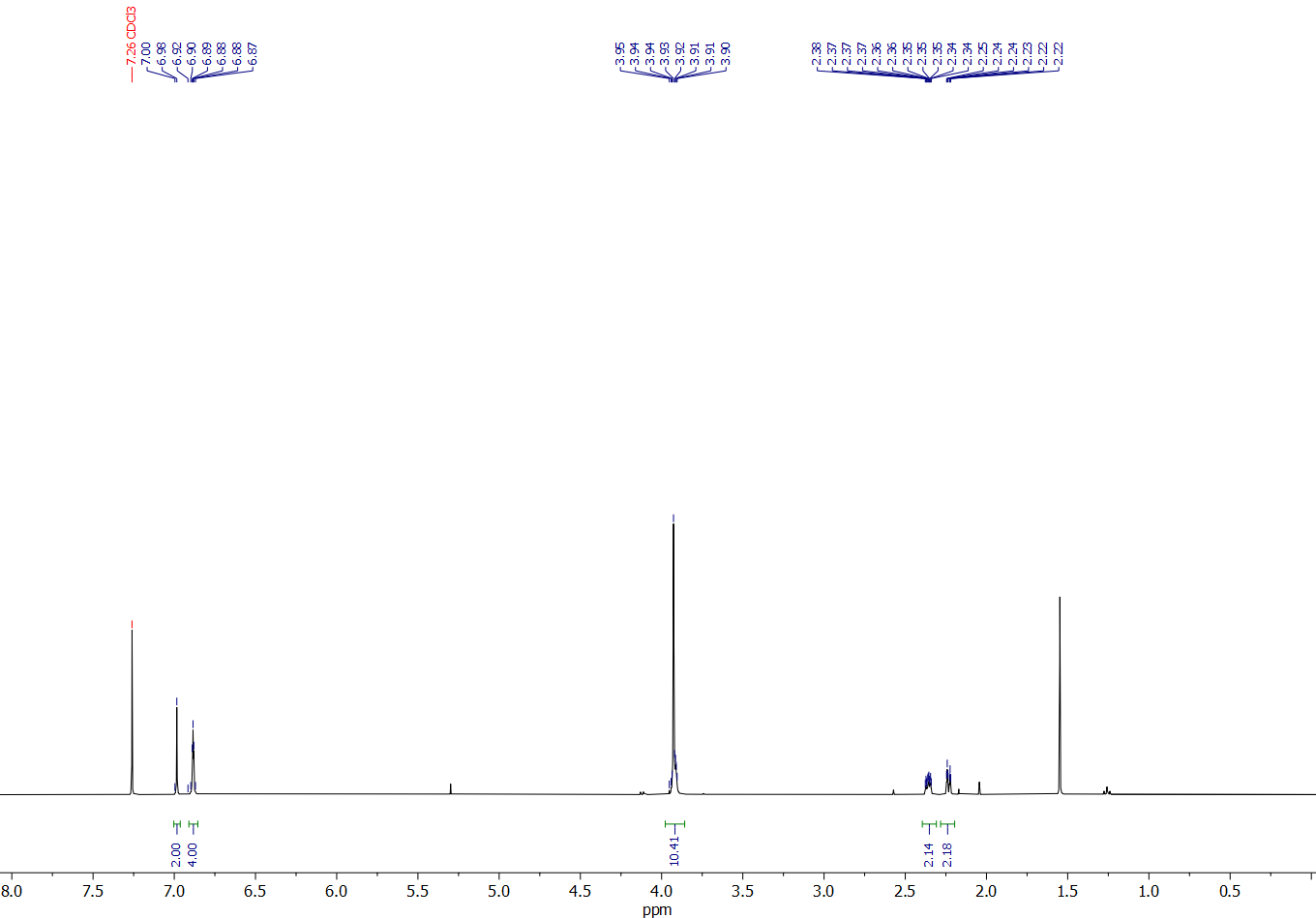

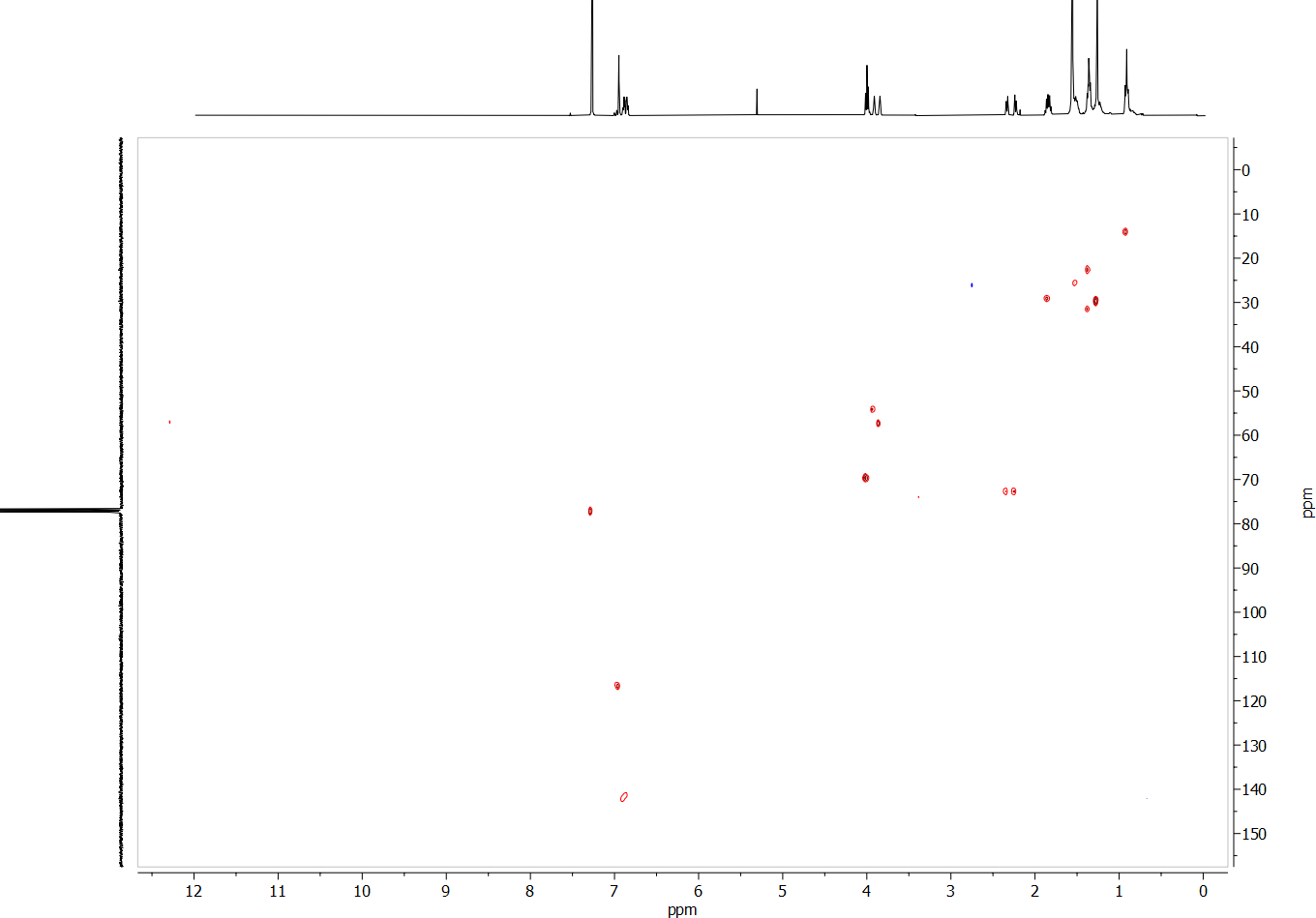
**Figure S5.** HSQC NMR spectrum of **NBD-P2** in CDCl_3_.

**Figure S6.** ^1^H-NMR spectrum of **NBD-O1** in CDCl_3_ at 400 MHz.

**Figure S7.** ^13^C-NMR spectrum of **NBD-O1** in CDCl_3_ at 101 MHz.
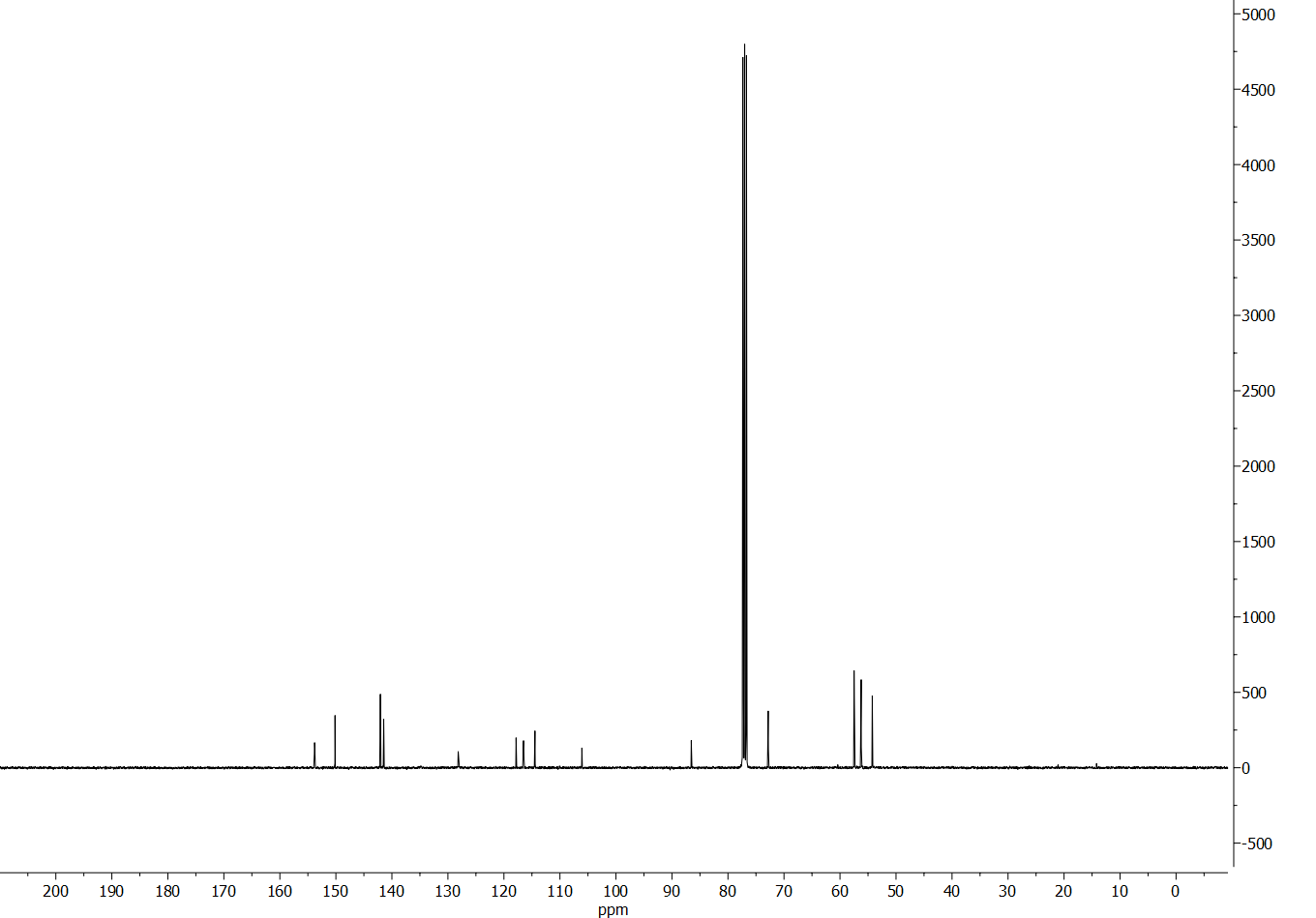


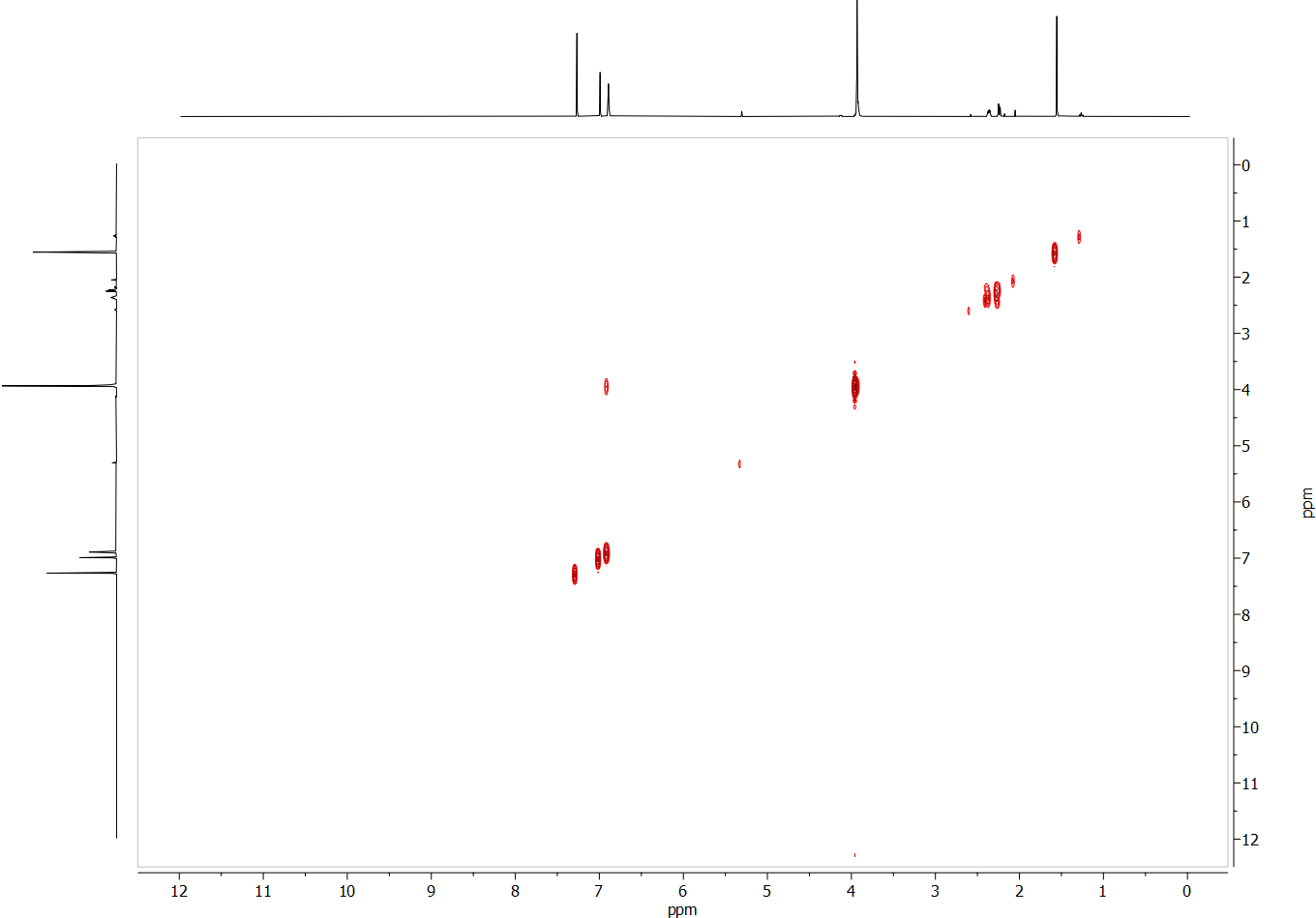
**Figure S8.** COSY NMR spectrum of **NBD-O1** in CDCl_3_.

**
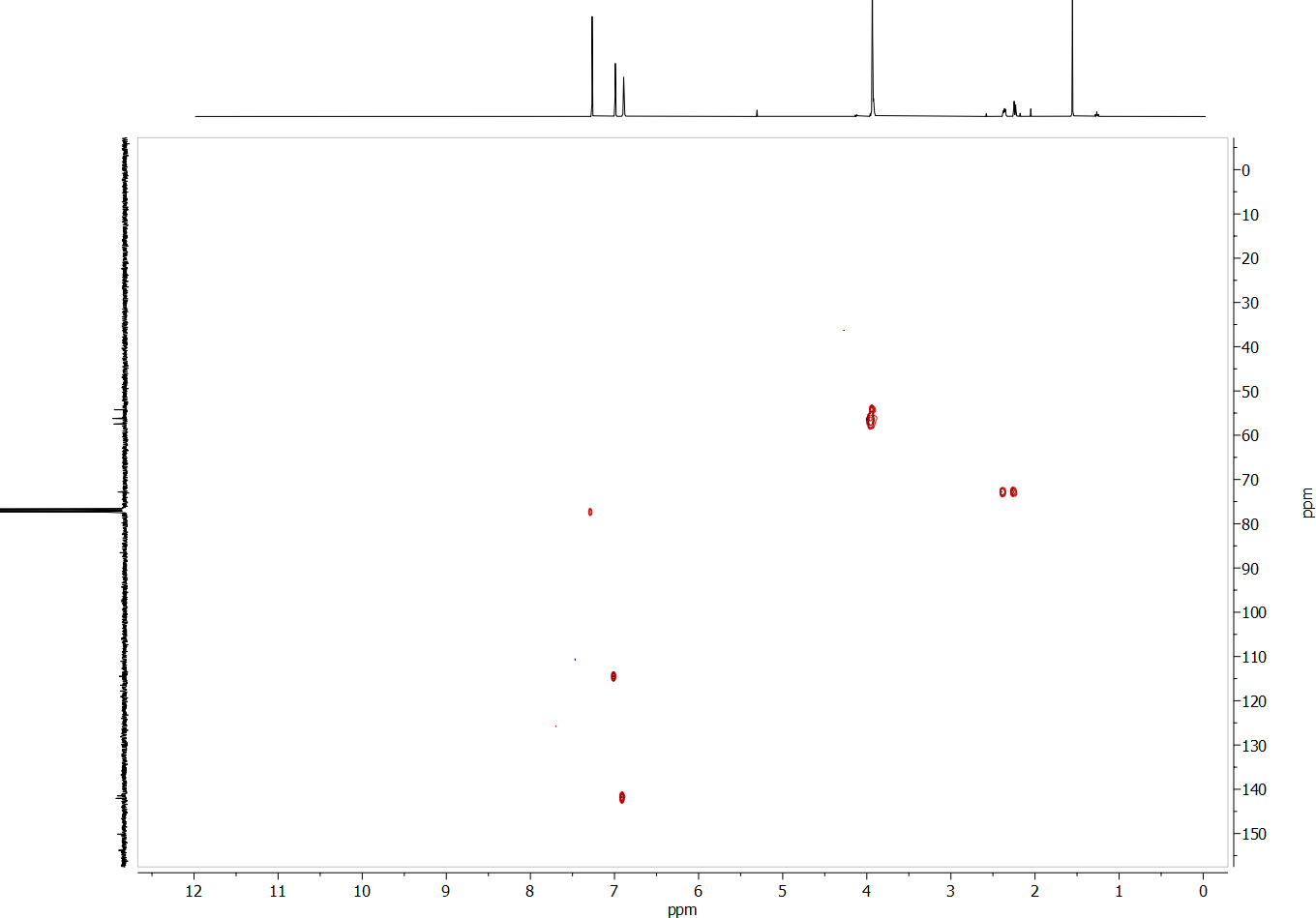
Figure S9.** HSQC NMR spectrum of **NBD-O1** in CDCl_3_.


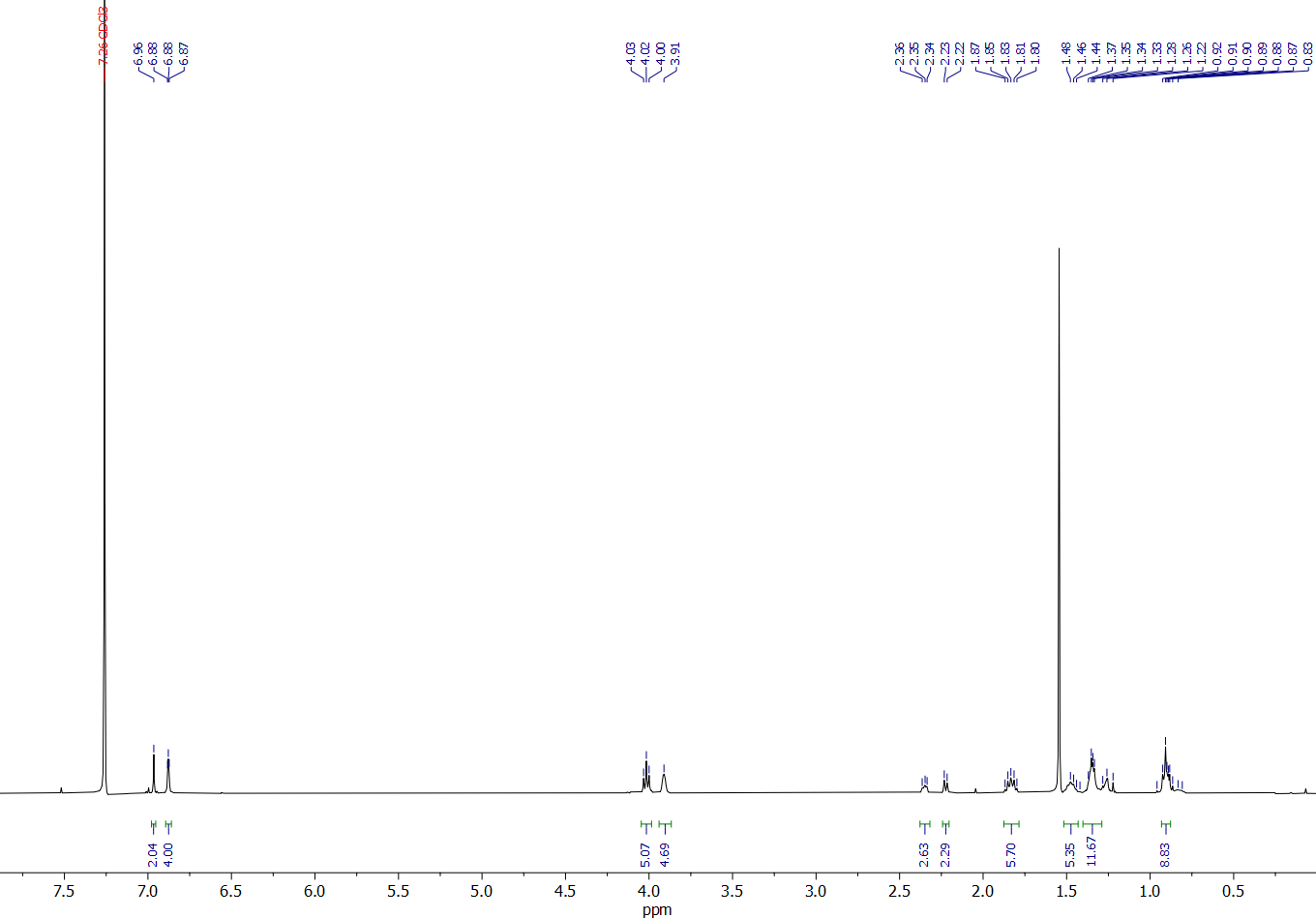


**Figure S10.** ^1^H-NMR spectrum of **NBD-O2** in CDCl_3_ at 400 MHz.


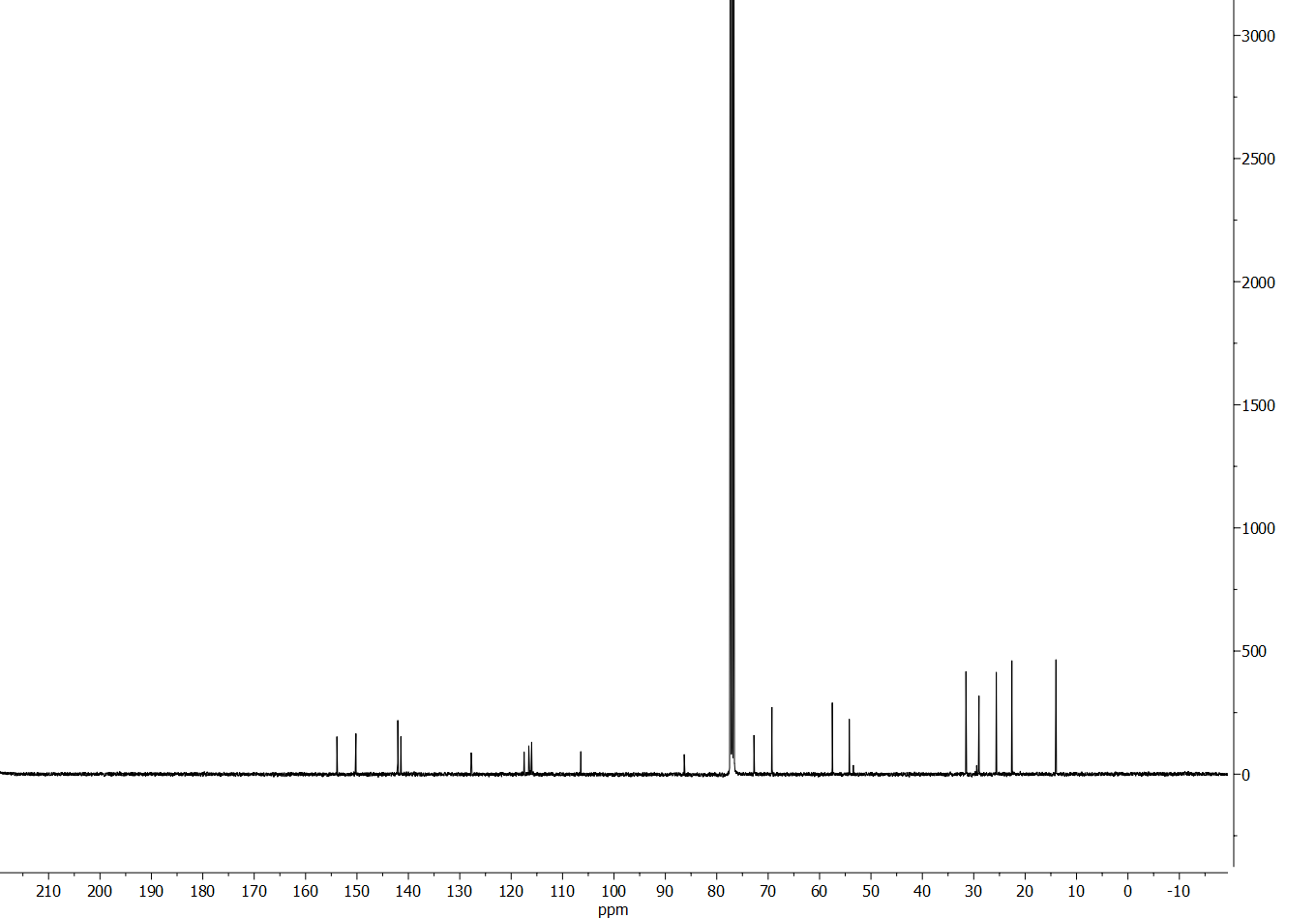


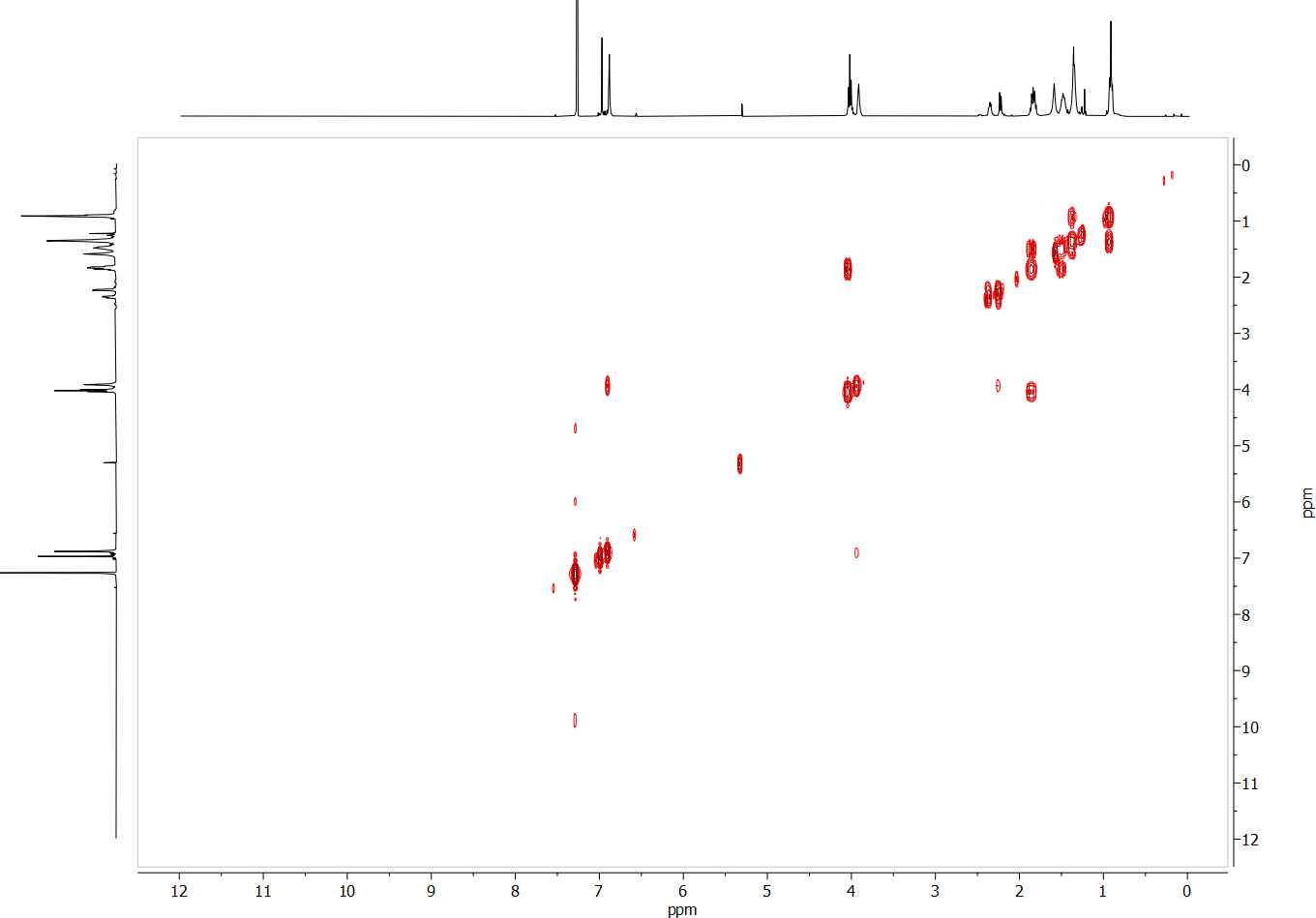
**Figure S11.** ^13^C-NMR spectrum of **NBD-O2** in CDCl_3_ at 101 MHz.

**Figure S12.** COSY NMR spectrum of **NBD-O2** in CDCl_3_.


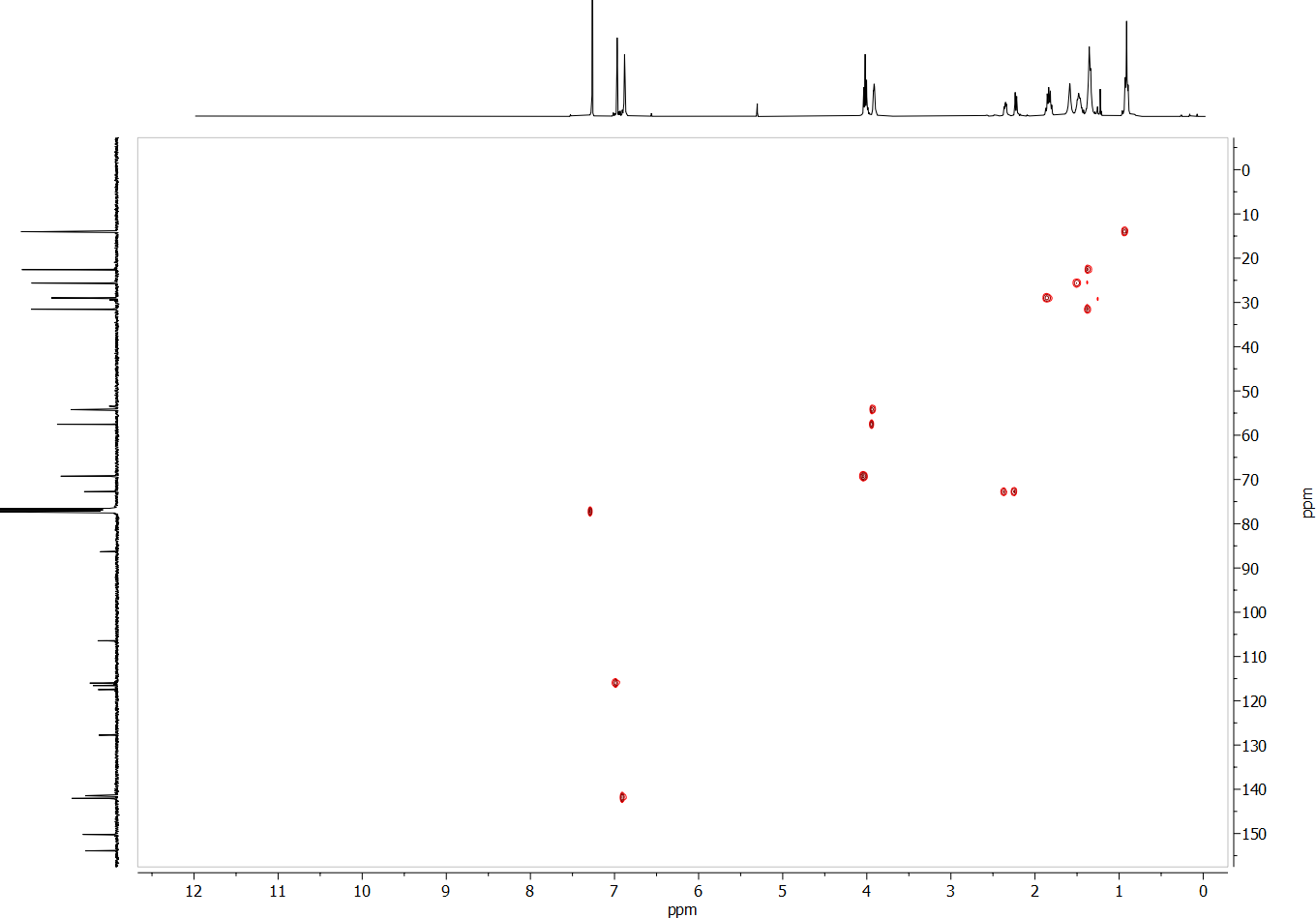


**Figure S13.** HSQC NMR spectrum of **NBD-O2** in CDCl_3_.

# 4 NMR irradiation studies


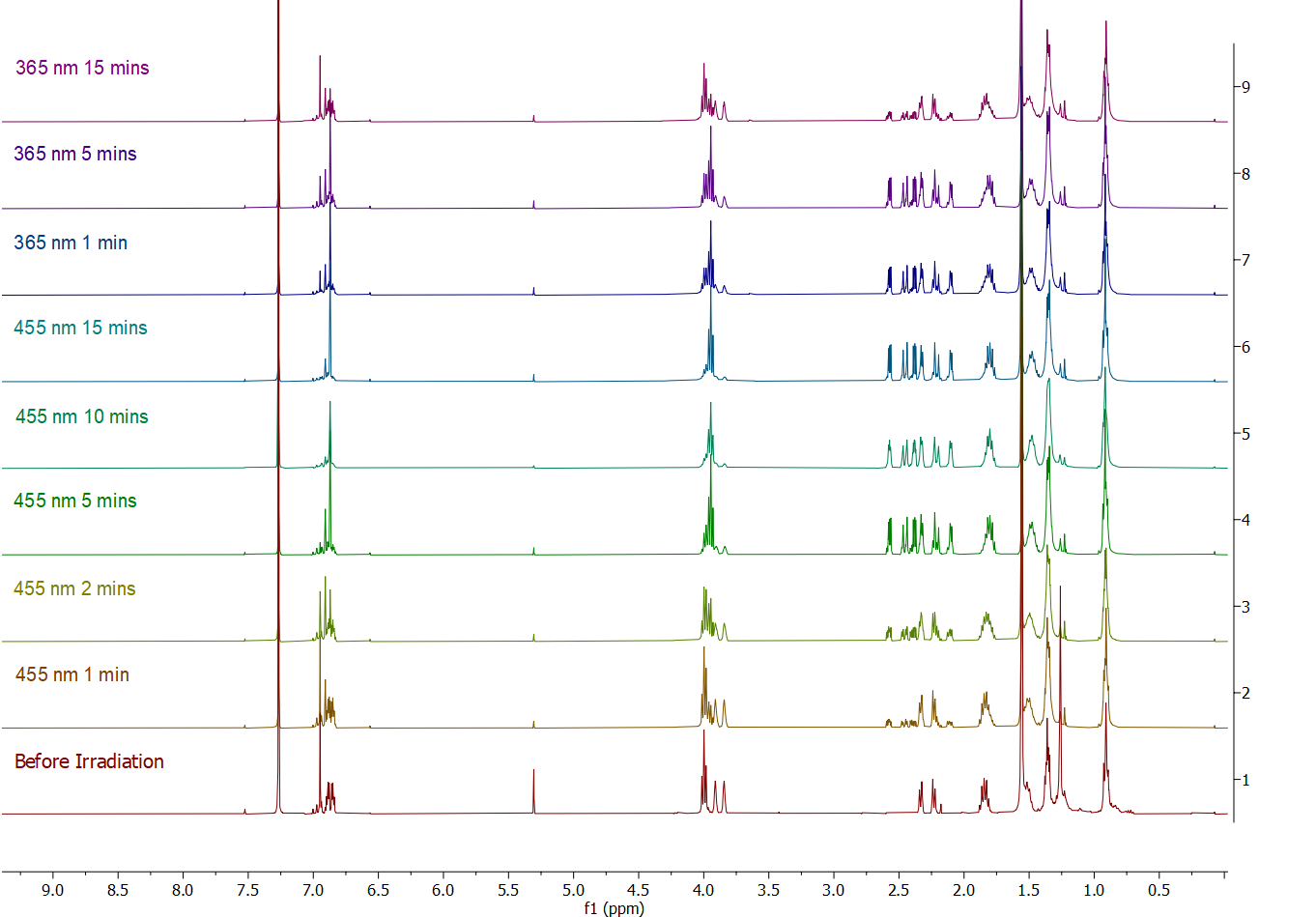


NBD-NBD

NBD-QC

QC-QC

**Figure S14.** ^1^H NMR spectra following the irradiation of **NBD-P2** in CDCl_3_ under 455 nm irradiation, showing conversion from NBD-NBD to QC-QC. Back conversion to a photostationary state (PSS) is triggered upon irradiation with 365 nm.


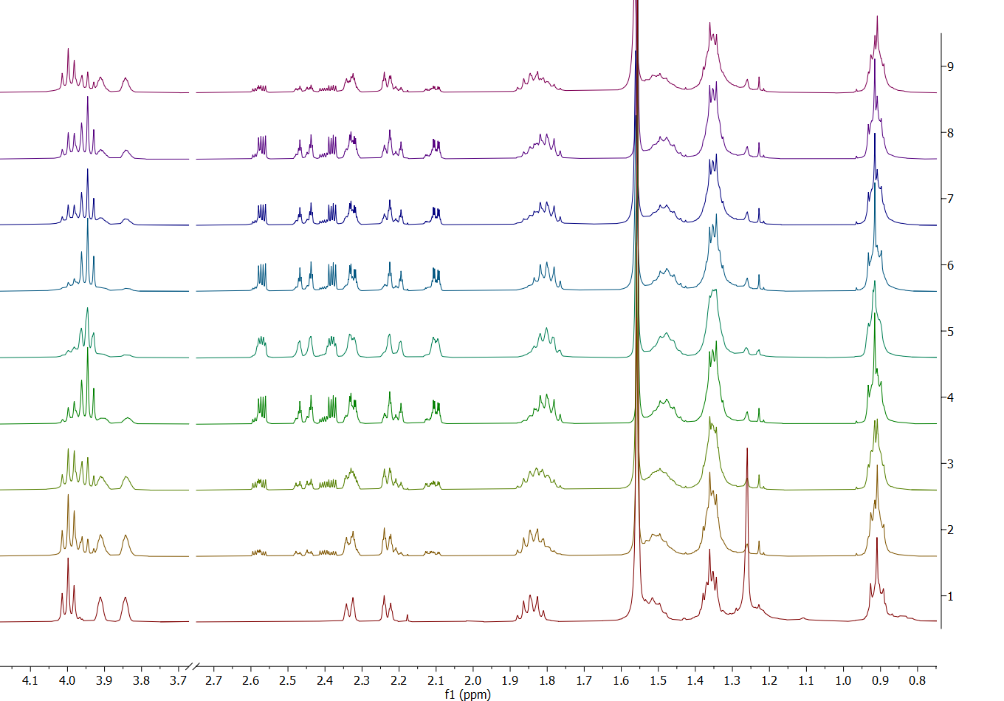


**Figure S15.** Zoom in of the aliphatic region of the spectra shown in **Figure S14** of the photoconversion and back conversion of **NBD-P2**.


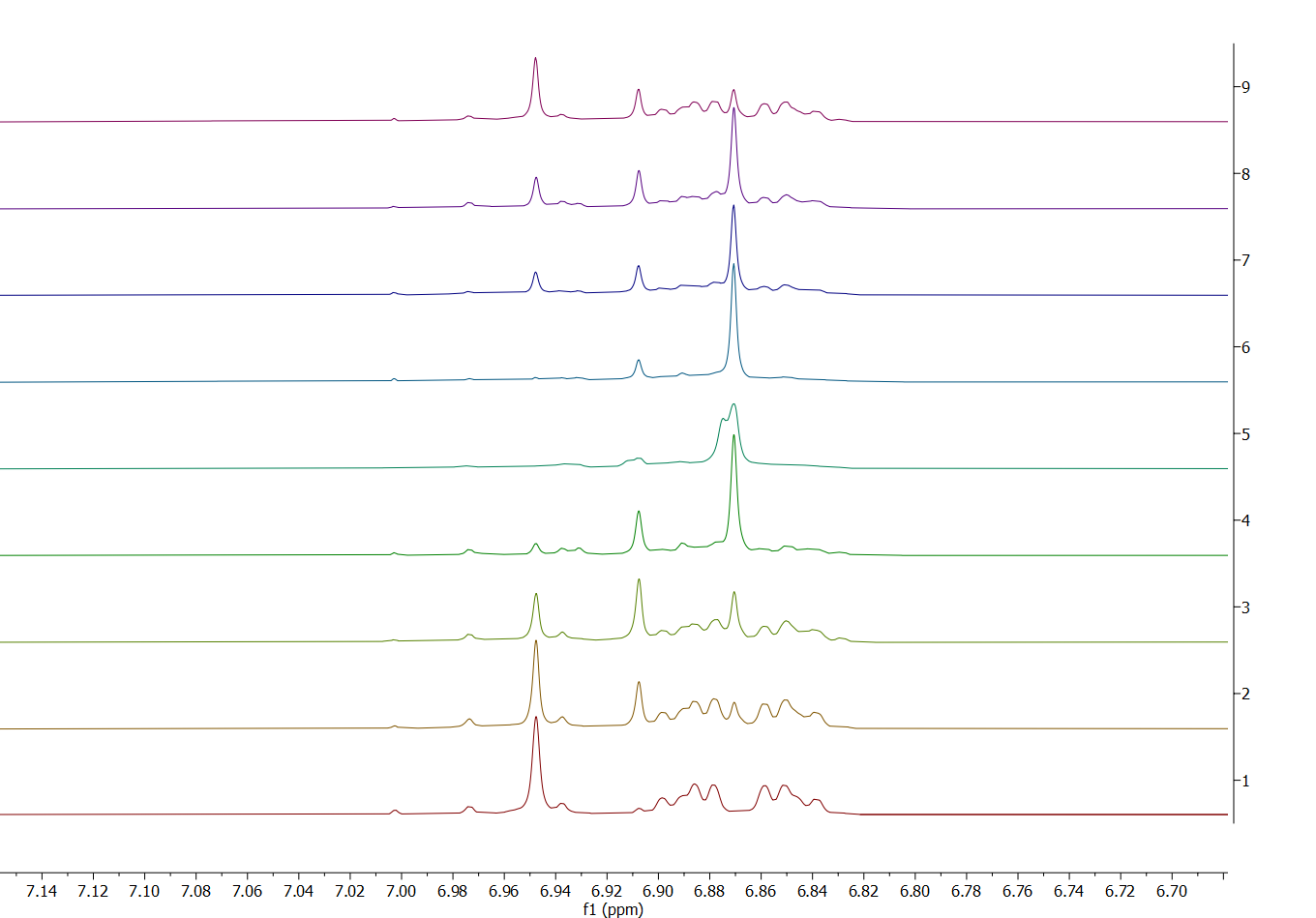


QC-QC

NBD-QC

NBD-NBD

**Figure S16.** Zoom in of the aromatic region of the spectra shown in **Figure S14** to show the singlet peak corresponding to the central phenyl ring aromatic proton used for the integration and determination of the composition of the isomers in the photostationary state (PSS) of **NBD-P2**.

**
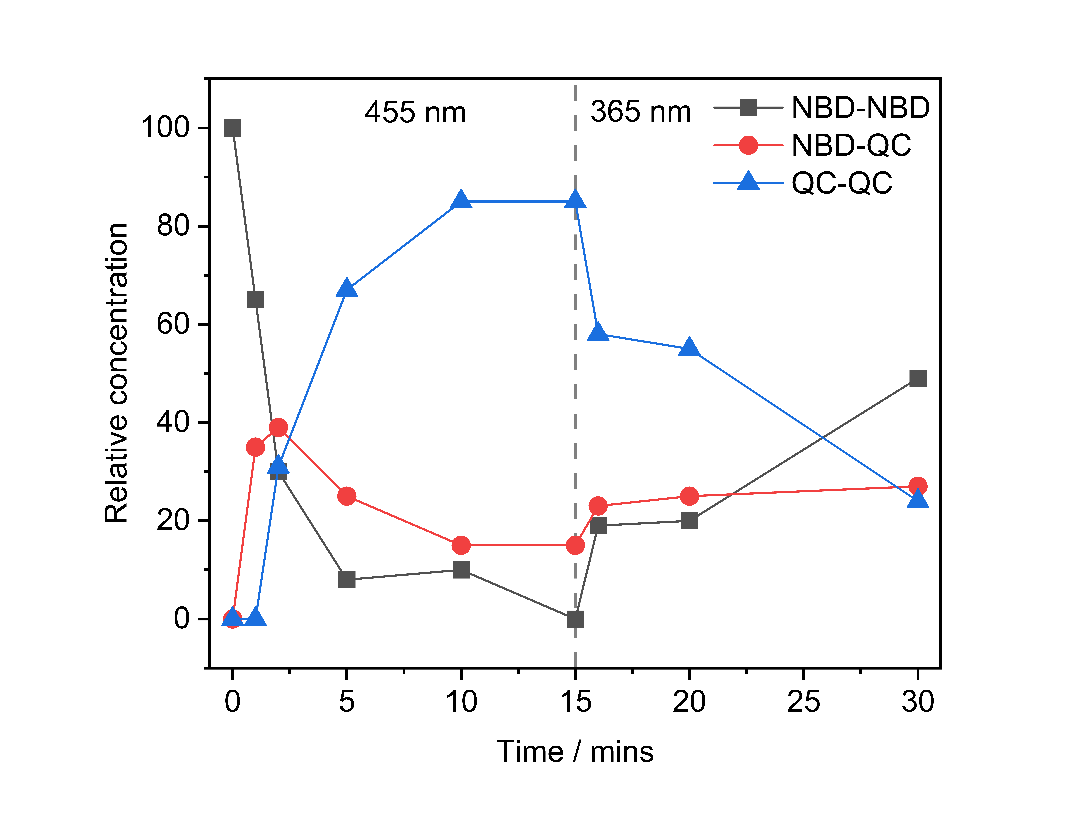
Figure S17.** Percentage conversion of each isomer of **NBD-P2** after irradiation with 455 nm and subsequent irradiation with 365 nm, determined by integrating the singlet peak in the aromatic region corresponding to the proton on the central phenyl ring (orange circle, **Figure S14**).


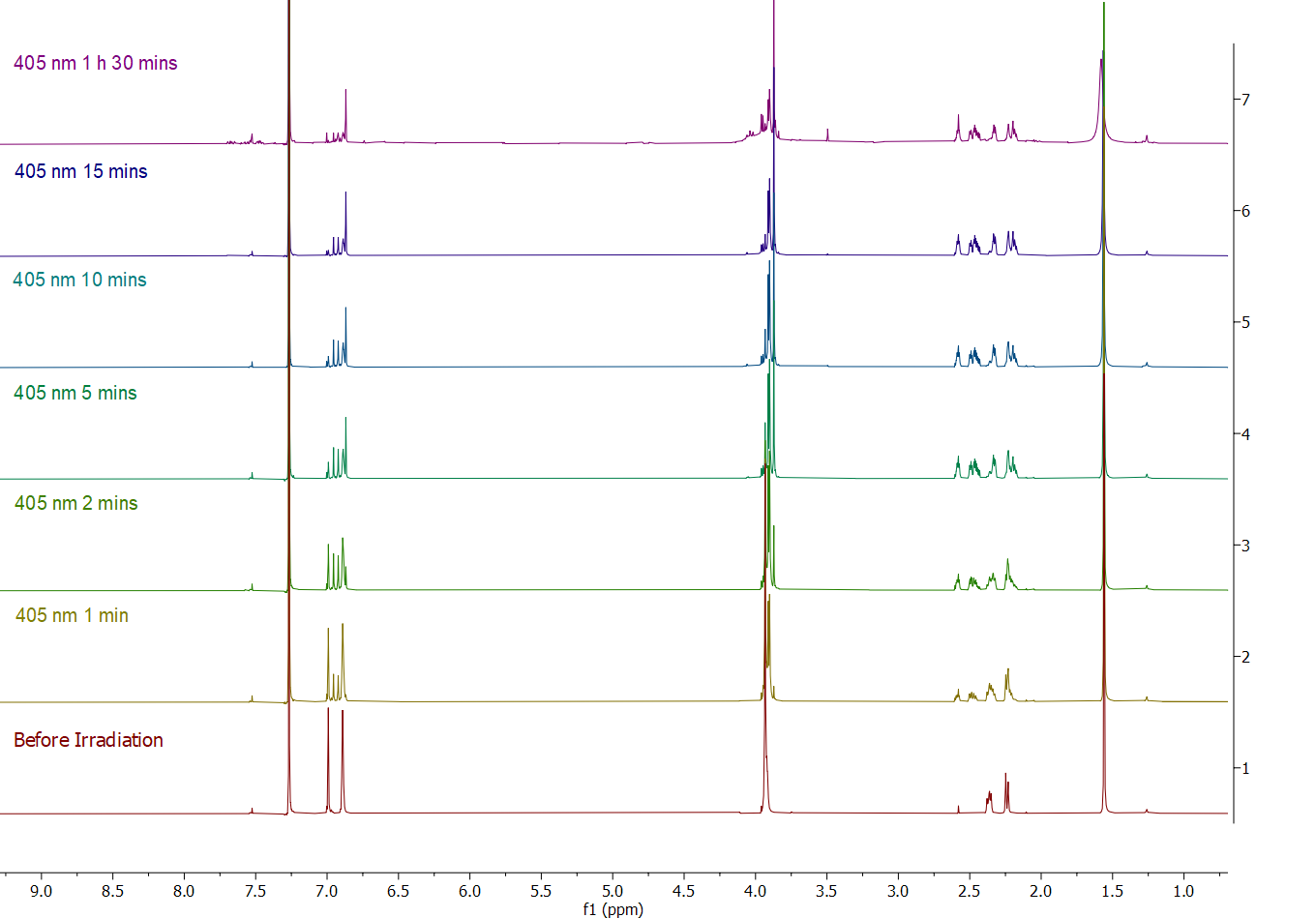


QC-QC

NBD-QC

NBD-NBD

**Figure S18.** ^1^H NMR spectra following the irradiation of **NBD-O1** in CDCl_3_ under 405 nm irradiation, showing conversion from NBD-NBD to QC-QC and degradation under prolonged irradiation.


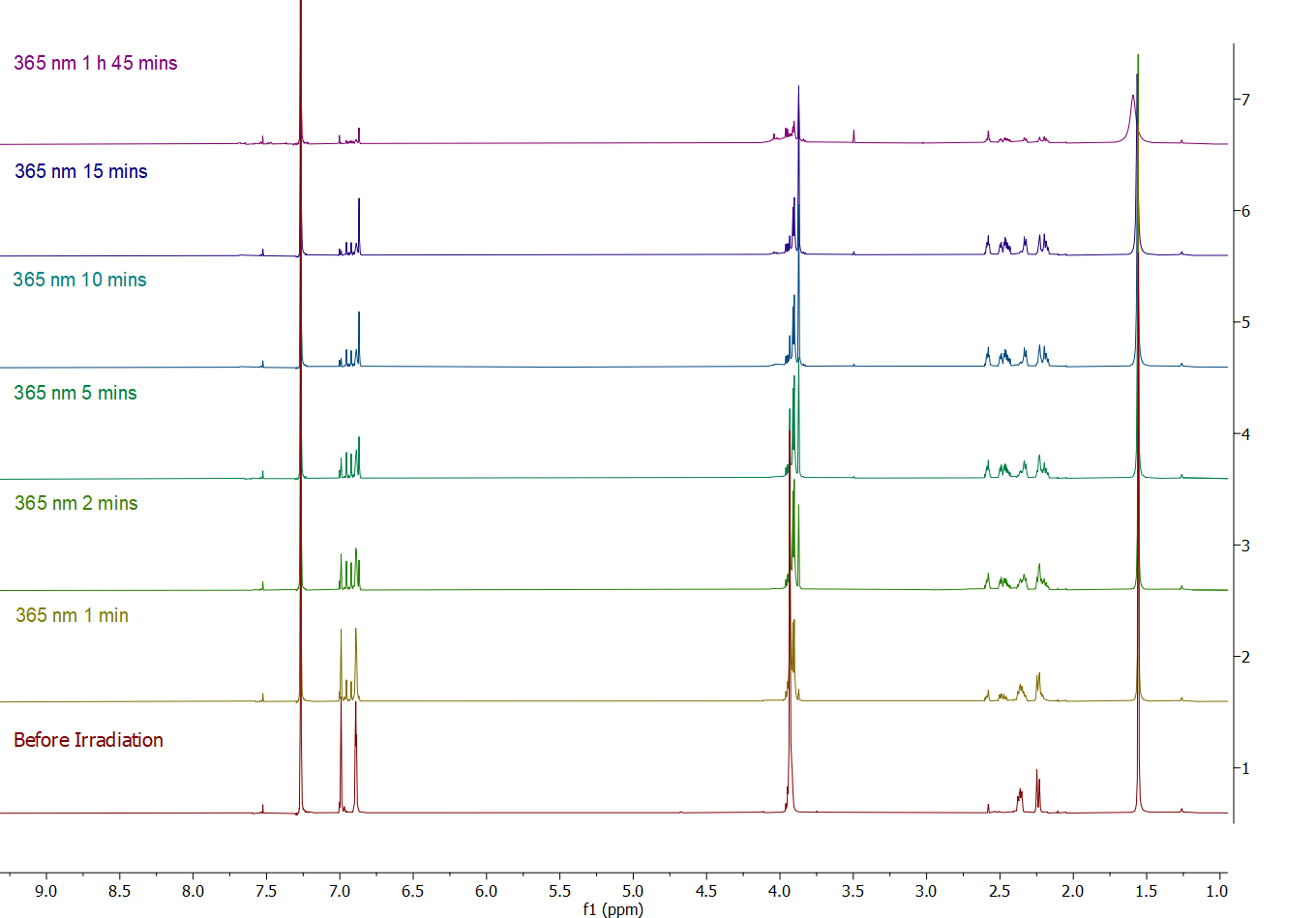

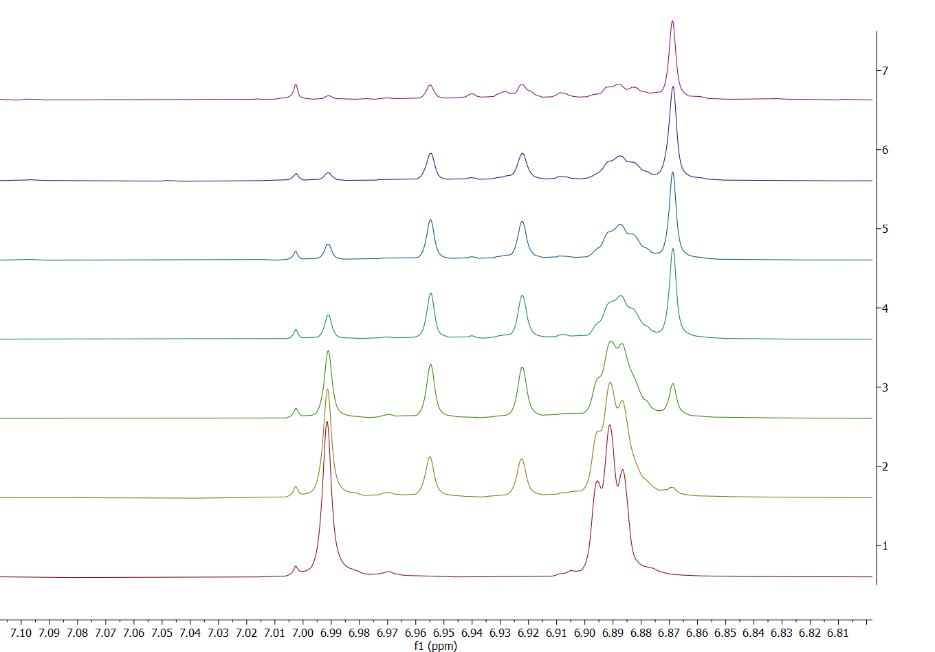
**Figure S19.** Zoom in of the aromatic region of the spectra shown in **Figure S18** for **NBD-O1** to show the singlet peak(s) corresponding to the central phenyl ring aromatic proton used for the integration and determination of the composition of the isomers in the photostationary state (PSS).

NBD-NBD

QC-QC

NBD-QC

**Figure S20. (a)** ^1^H NMR spectra following the irradiation of **NBD-O1** in CDCl_3_ under 365 nm irradiation, showing conversion from NBD-NBD to QC-QC. Degradation is observed under prolonged irradiation.


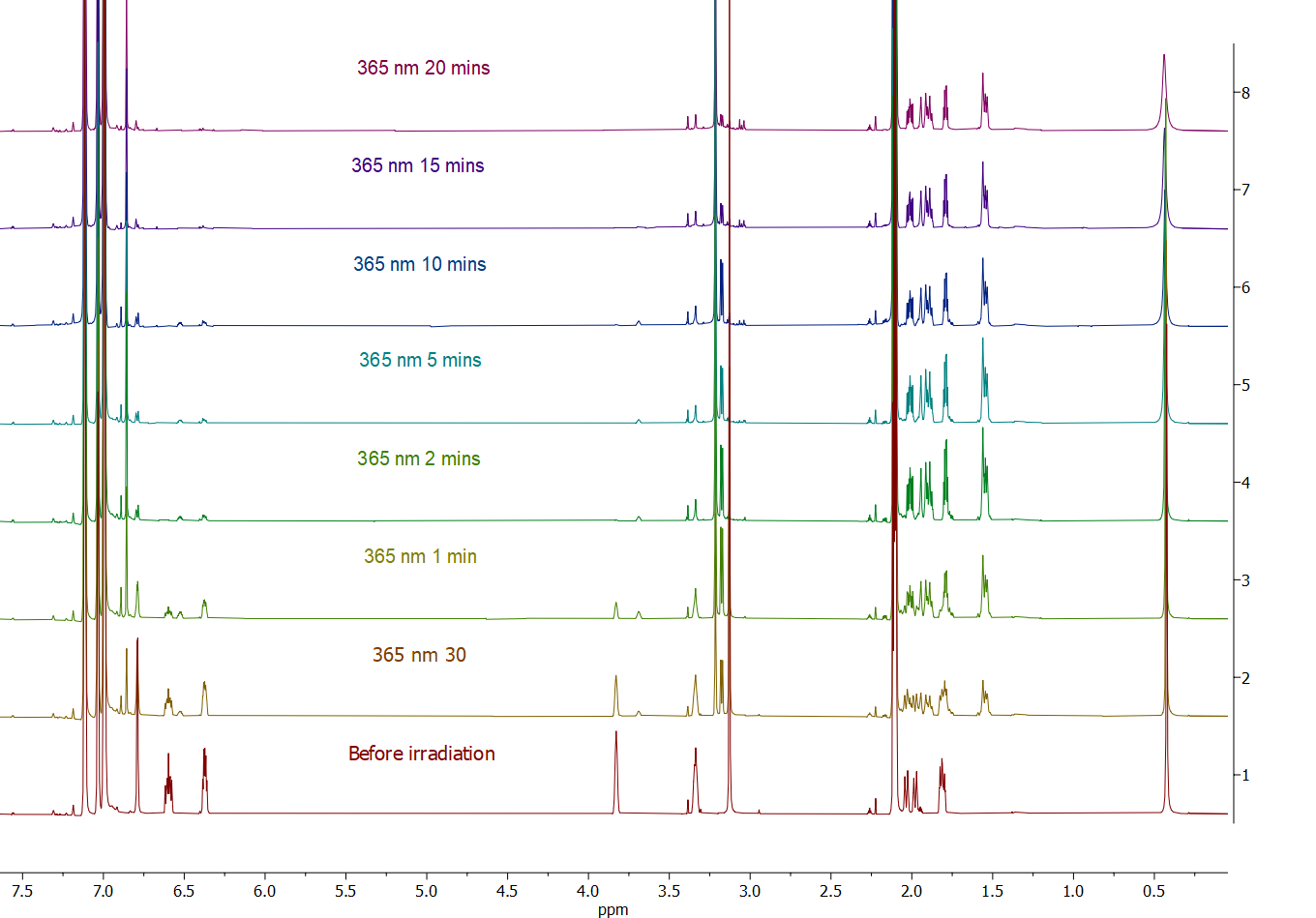
**Figure S20. (b)** ^1^H NMR spectra following the irradiation of **NBD-O1** in toluene-d_8_ under 365 nm irradiation, showing conversion from NBD-NBD to QC-QC. Prolonged irradiation led to precipitation of a solid.


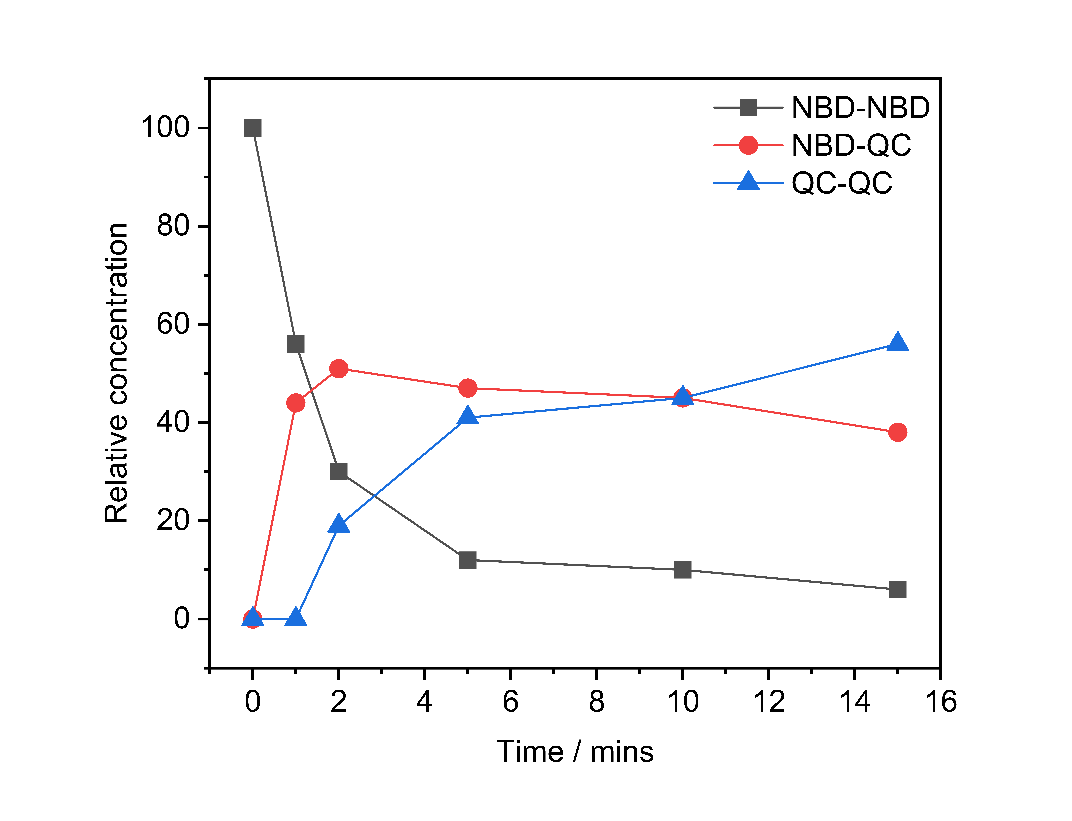


**Figure S21.** Percentage conversion of each isomer of **NBD-O1** after irradiation with 405 nm, determined by integrating the singlet peak(s) in the aromatic region corresponding to the proton on the central phenyl ring (orange circle, **Figure S18**).


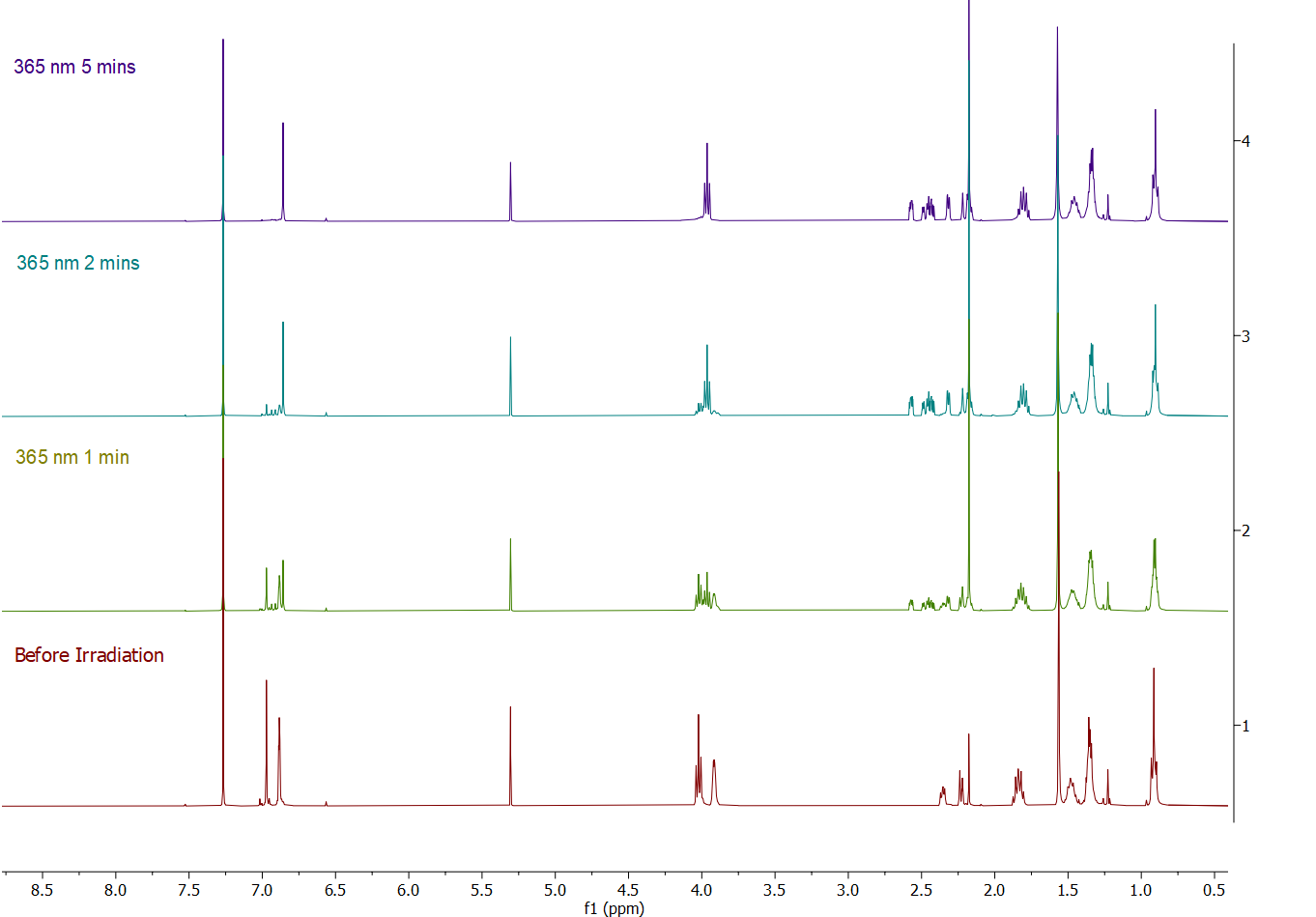


NBD-QC

QC-QC

NBD-NBD

**Figure S22.** ^1^H NMR spectra following the irradiation of **NBD-O2** in CDCl_3_ under 365 nm irradiation, showing conversion from NBD-NBD to QC-QC. Full conversion is triggered after 5 minutes of irradiation.


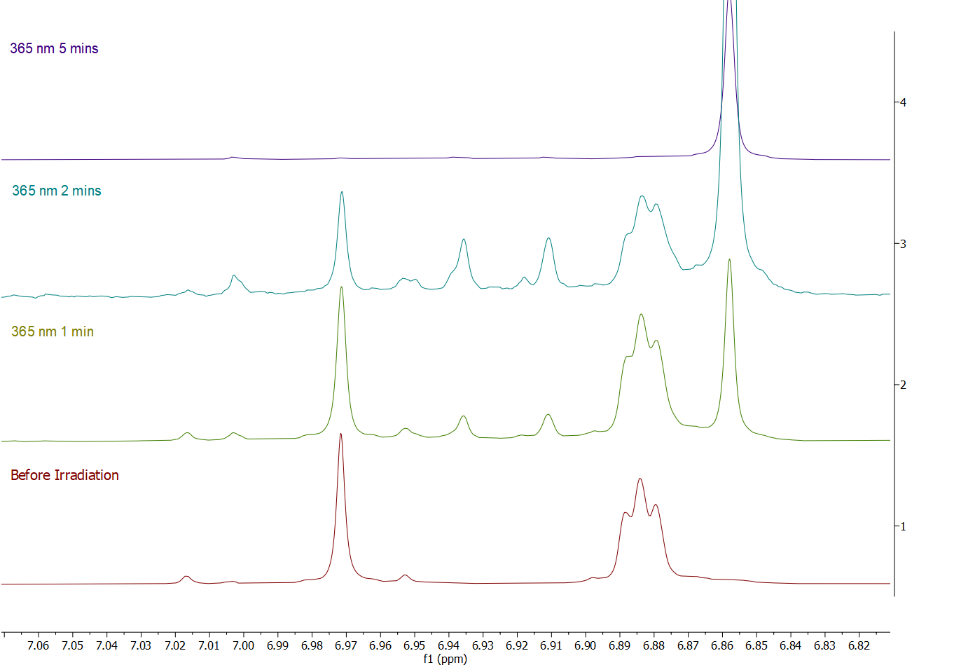


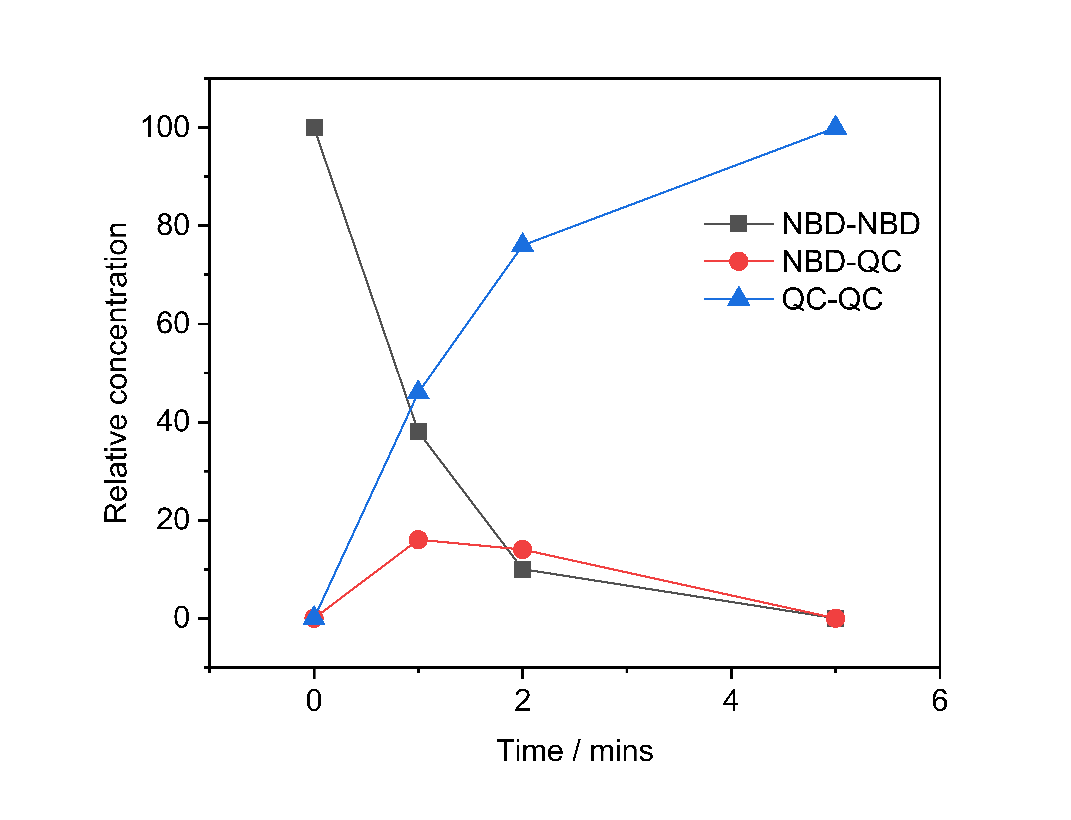
**Figure S23.** Zoom in of the aromatic region of the spectra shown in **Figure S22** for **NBD-O2** to show the singlet peak(s) corresponding to the central phenyl ring aromatic proton used for the integration and determination of the composition of the isomers in the photostationary state (PSS).

**Figure S24.** Percentage conversion of each isomer of **NBD-O2** after irradiation with 365 nm, determined by integrating the singlet peak(s) in the aromatic region corresponding to the proton on the central phenyl ring (orange circle, **Figure S22**)

# 5 Quantum yield determination and photoswitching behaviour


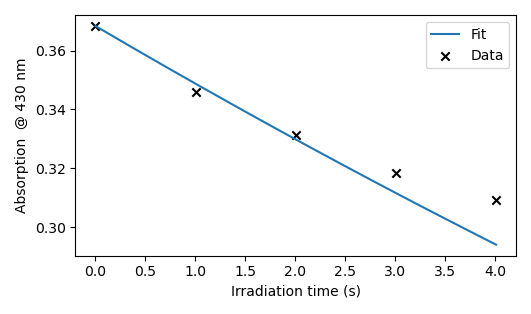
**NBD-P1 in toluene**

**Figure S25.** QY measurement for **NBD-P1** in toluene (concentration = 1.84×10^-5^ M) upon 405 nm irradiation. Average QY = 7.1±0.3 % for the analysis wavelength of 430 nm.


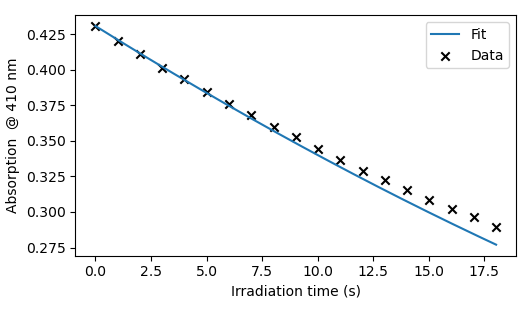


**Figure S26.** QY measurement for **NBD-P1** in toluene (concentration = 1.84×10^-5^ M) upon 405 nm irradiation. Average QY = 3.5±0.2 % for the analysis wavelength of 410 nm.


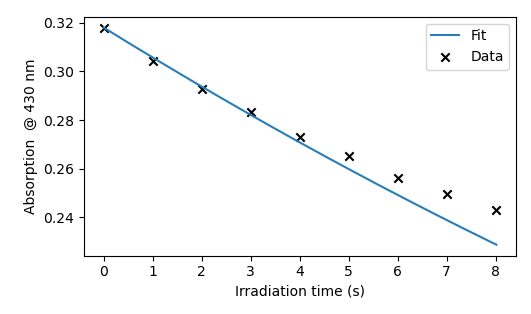
The QY fit for **NBD-P1** is better for the analysis wavelength of 410 nm compared to 430 nm. However, the QY at the analysis wavelength of 410 nm is lower due to competitive absorption, hence going forward the lower energy analysis wavelength was chosen for the QY determination.

**Figure S27.** QY measurement for **NBD-P1** in toluene (concentration = 1.84×10^-5^ M) upon 430 nm irradiation. Average QY = 10.4±0.2% for the analysis wavelength of 430 nm.


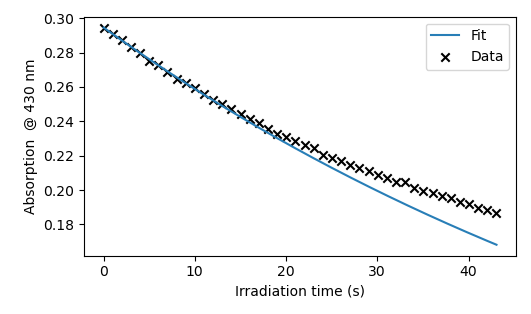


**Figure S28.** QY measurement for **NBD-P1** in toluene (concentration = 1.84×10^-5^ M) upon 455 nm irradiation. Average QY = 12.7±0.1% for the analysis wavelength of 430 nm.

**
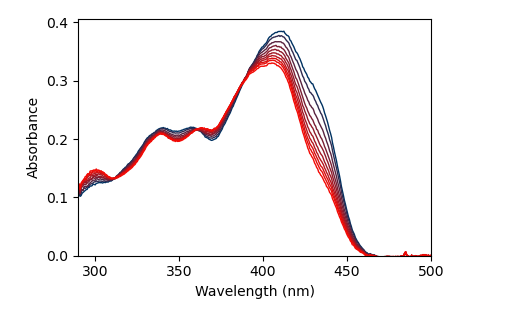

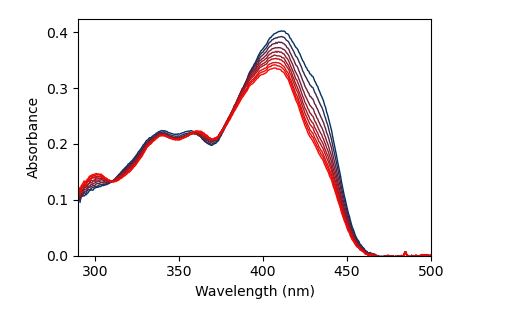
Figure S29.** Spectral change of **NBD-P1** in toluene upon irradiation at 430 nm (Left) over 80 seconds and at 455 nm (Right) over 60 seconds.


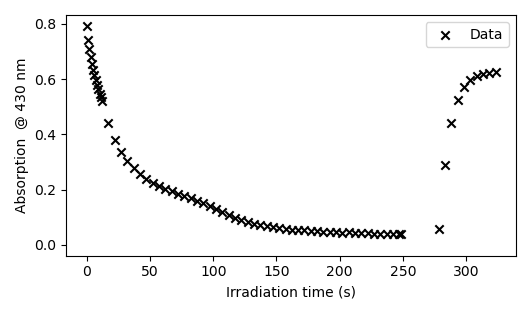


**Figure S30.** Irradiation of **NBD-P1** in toluene, first with 405 nm LED showing conversion (shaded grey area), followed by photochemical back-conversion upon irradiation with 365 nm.


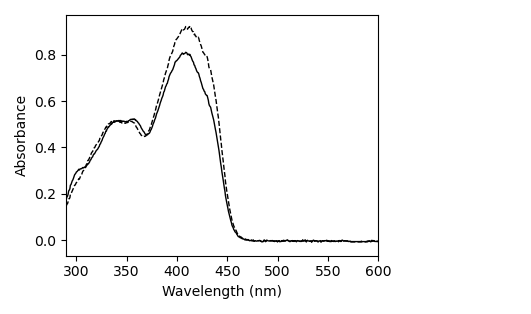

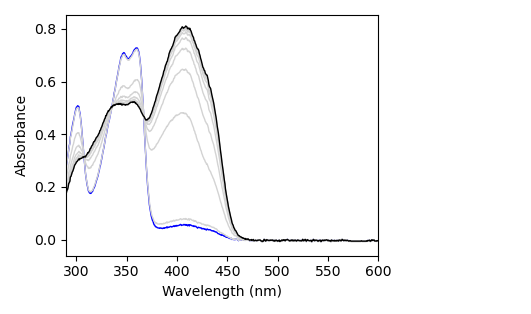

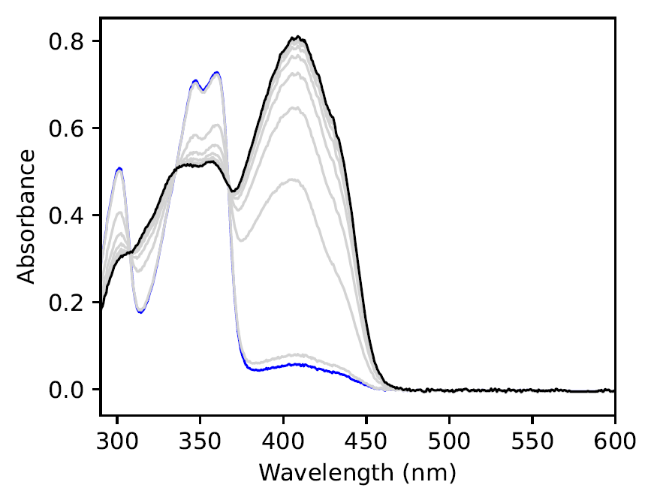

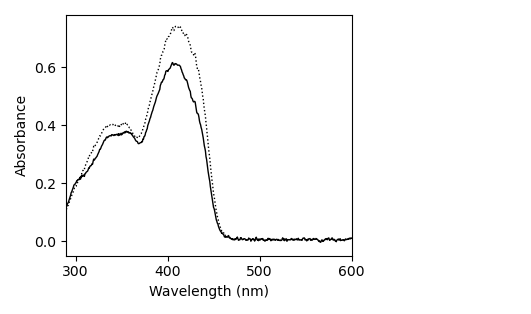


365 nm

365 nm

**Figure S31.** Spectra of the photochemical back-conversion of **NBD-P1** in toluene. Left: The blue line shows the spectrum after conversion upon irradiation with 405 nm, and subsequent irradiation at 365 nm to recover the NBD-NBD isomer. Right: full recovery of the initial NBD-NBD form (dashed line) is not achieved upon 365 nm conversion, instead a photostationary state is reached (black solid line).


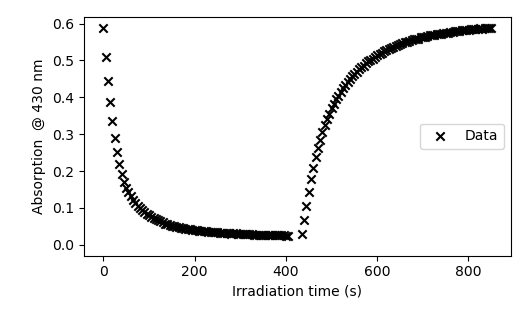


**Figure S32.** Irradiation of **NBD-P1** in toluene, first with 405 nm LED showing conversion (shaded grey area), followed by photochemical back-conversion upon irradiation with 340 nm.

340 nm


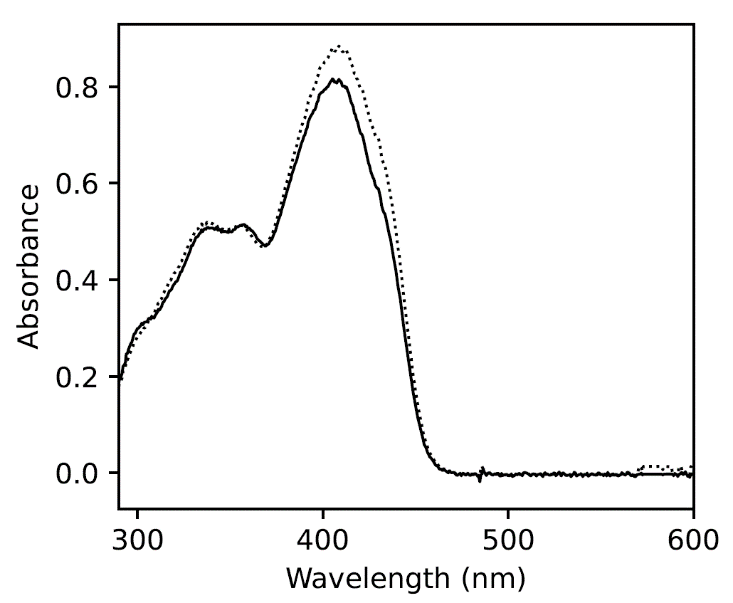

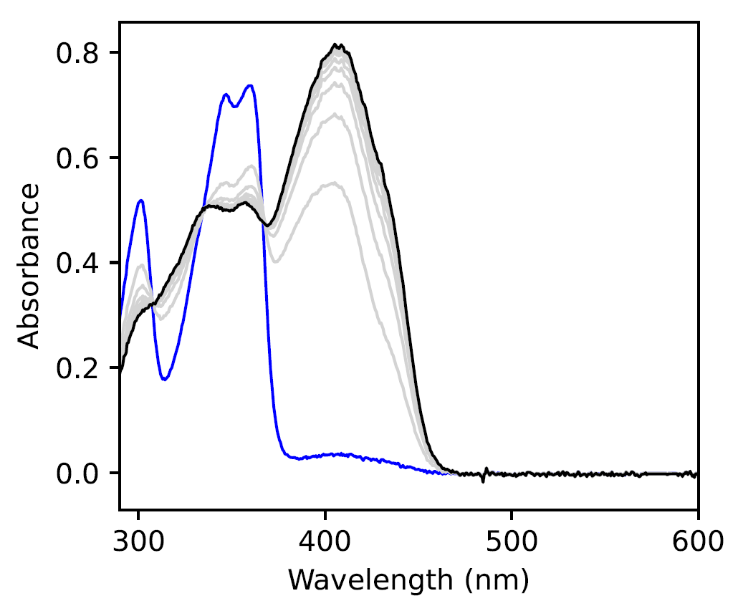


**Figure S33.** Spectra of the photochemical back-conversion of **NBD-P1** in toluene. Left: The blue line shows the spectrum after conversion upon irradiation with 405 nm, and subsequent irradiation at 340 nm to recover the NBD-NBD isomer. Right: full recovery of the initial NBD-NBD form (dashed line) is not achieved upon 340 nm conversion, instead a photostationary state is reached (black solid line).


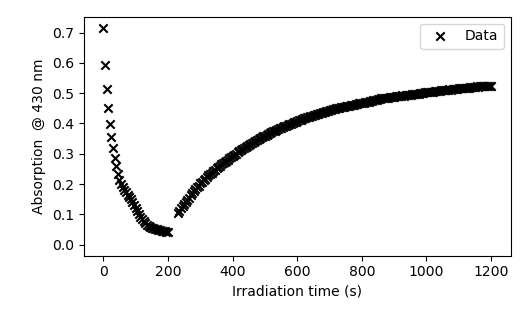
**Figure S34.** Irradiation of **NBD-P1** in toluene, first with 405 nm LED showing conversion (shaded grey area), followed by photochemical back-conversion upon irradiation with 308 nm.


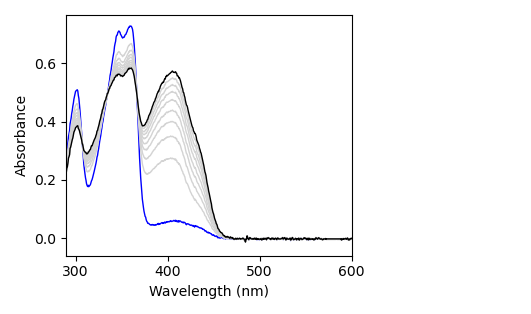


308 nm


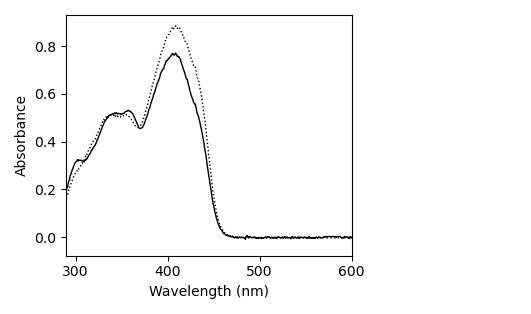


**Figure S35.** Spectra of the photochemical back-conversion of **NBD-P1** in toluene. Left: The blue line shows the spectrum after conversion upon irradiation with 405 nm, and subsequent irradiation at 308 nm to recover the NBD-NBD isomer. Right: full recovery of the initial NBD-NBD form (dashed line) is not achieved upon 308 nm conversion, instead a photostationary state is reached (black solid line).

**NBD-P1 in acetonitrile**


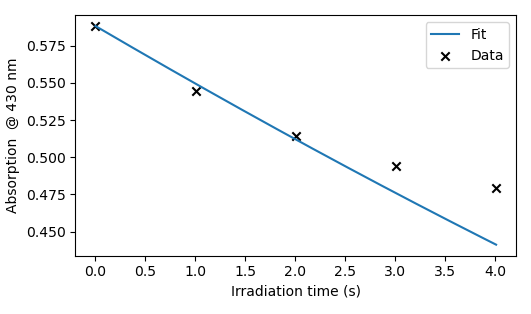
**Figure S36.** QY measurement for **NBD-P1** in acetonitrile (concentration = 3.06×10^-5^ M) upon 405 nm irradiation. Average QY = 11.5±0.5 % for the analysis wavelength of 430 nm.


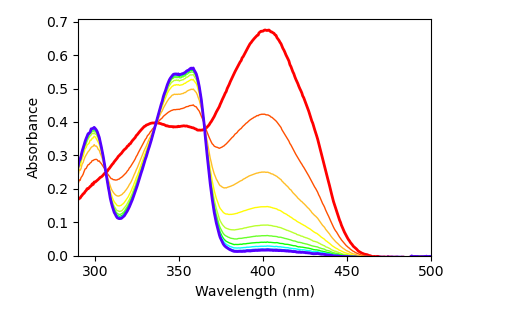


**Figure S37.** Conversion of **NBD-P1** in acetonitrile upon 405 nm irradiation showing full conversion after 240 seconds. Red: t = 0 s; Blue: t = 240 s.


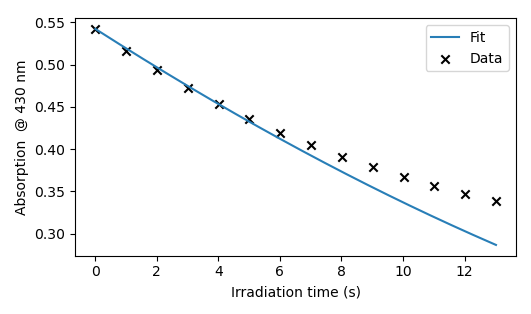


**Figure S38.** QY measurement for **NBD-P1** in acetonitrile (concentration = 3.06×10^-5^ M) upon 430 nm irradiation. Average QY = 14.1±0.2 % for the analysis wavelength of 430 nm.


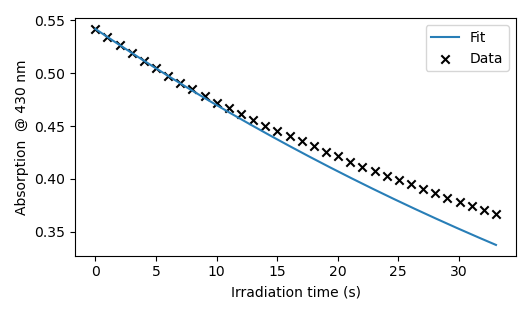
**Figure S39.** QY measurement for **NBD-P1** in acetonitrile (concentration = 3.06×10^-5^ M) upon 455 nm irradiation. Average QY = 27.4±0.1 % for the analysis wavelength of 430 nm.


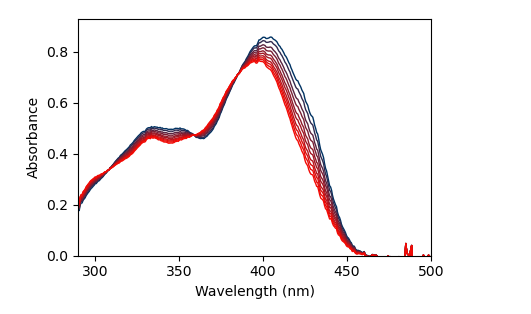

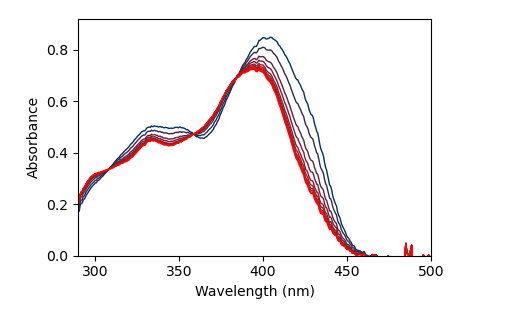


**Figure S40.** Spectral change of **NBD-P1** in acetonitrile upon irradiation at 430 nm (Left) over 50 seconds and at 455 nm (Right) over 50 seconds.


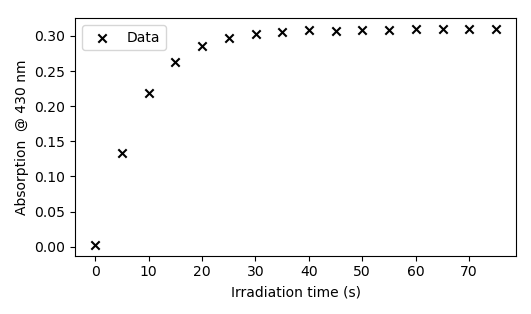


**Figure S41.** Photochemical backconversion of **NBD-P1** in acetonitrile, after first conversion with 405 nm LED, followed by photochemical back-conversion upon irradiation with 365 nm.


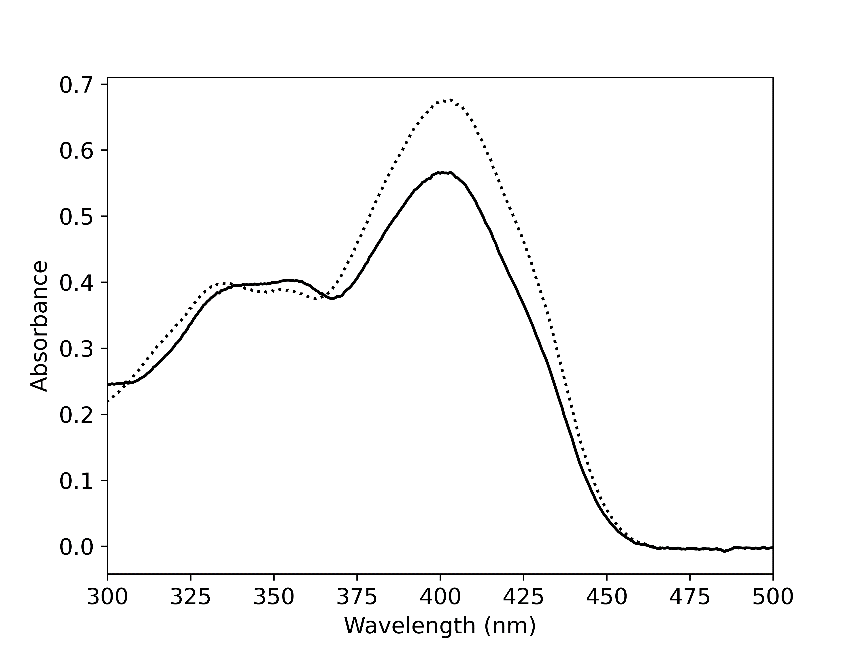

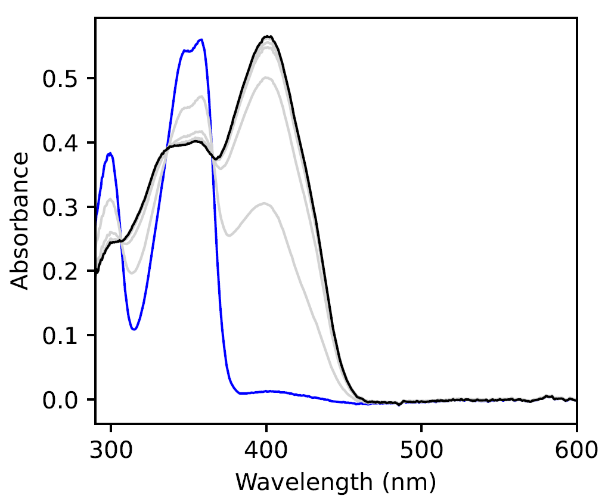


365 nm

**Figure S42.** Spectra of the photochemical back-conversion of **NBD-P1** in acetonitrile. Left: The blue line shows the spectrum after conversion upon irradiation with 405 nm, and subsequent irradiation at 365 nm to recover the NBD-NBD isomer. Right: full recovery of the initial NBD-NBD form (dashed line) is not achieved upon 365 nm conversion, instead a photostationary state is reached (black solid line).

**NBD-P2 in toluene**


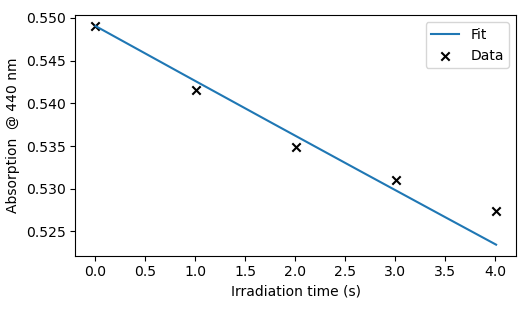


**Figure S43.** QY measurement for **NBD-P2** in toluene (concentration = 2.19×10^-5^ M) upon 405 nm irradiation. Average QY = 1.5±0.1 % for the analysis wavelength of 440 nm.


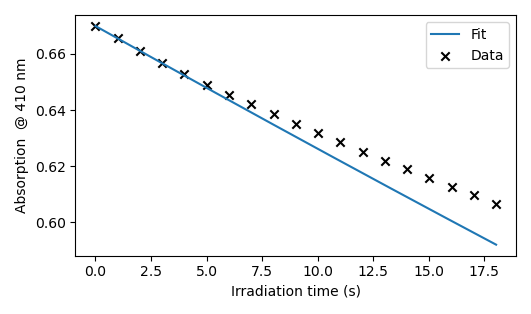


**Figure S44.** QY measurement for **NBD-P2** in toluene (concentration = 2.19×10^-5^ M) upon 405 nm irradiation. Average QY = 0.9±0.1 % for the analysis wavelength of 410 nm.


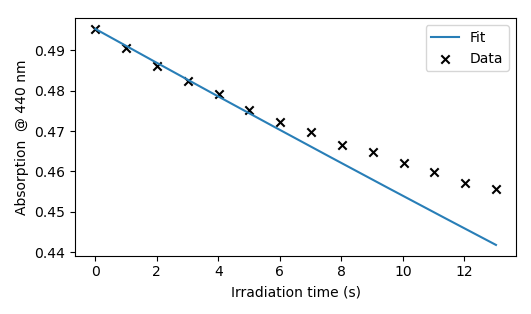


**Figure S45.** QY measurement for **NBD-P2** in toluene (concentration = 2.19×10^-5^ M) upon 430 nm irradiation. Average QY = 1.9±0.1 % for the analysis wavelength of 440 nm.


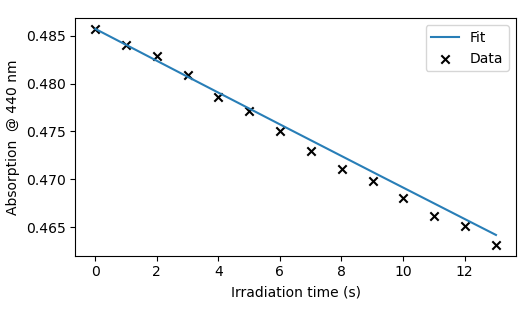
**Figure S46.** QY measurement for **NBD-P2** in toluene (concentration = 2.19×10^-5^ M) upon 455 nm irradiation. Average QY = 0.39±0.01 % for the analysis wavelength of 440 nm.


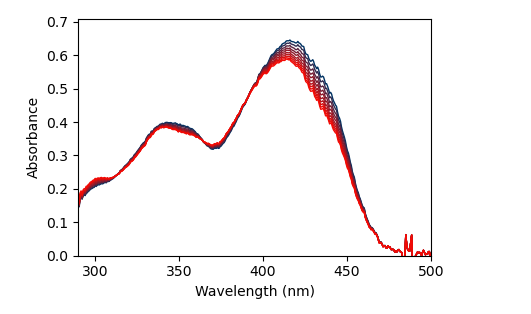

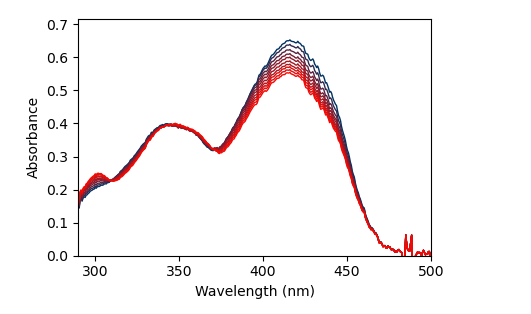


**Figure S47.** Spectral change of **NBD-P2** in toluene upon irradiation at 430 nm (Left) over 60 seconds and at 455 nm (Right) over 80 seconds.


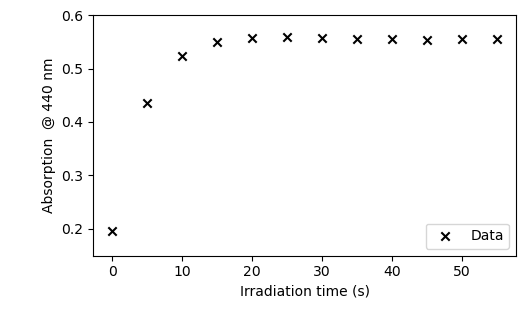


**Figure S48.** Photochemical backconversion of **NBD-P2** in toluene, after first conversion with 405 nm LED, followed by photochemical back-conversion upon irradiation with 365 nm.


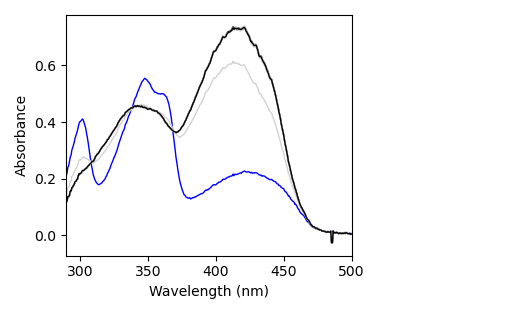

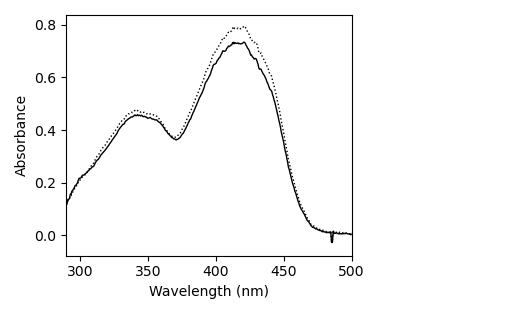


365 nm

**Figure S49.** Spectra of the photochemical back-conversion of **NBD-P2** in toluene. Left: The blue line shows the spectrum after conversion upon irradiation with 405 nm, and subsequent irradiation at 365 nm to recover the NBD-NBD isomer. Right: full recovery of the initial NBD-NBD form (dashed line) is not achieved upon 365 nm conversion, instead a photostationary state is reached (black solid line).


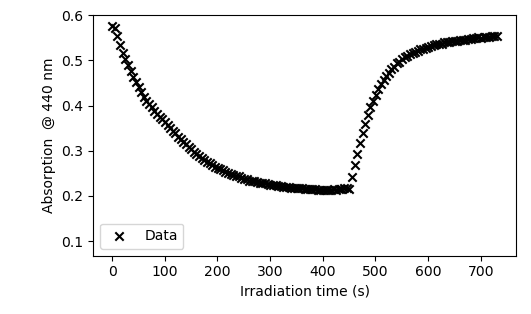


**Figure S50.** Irradiation of **NBD-P2** in toluene, first with 405 nm LED showing conversion (shaded grey area), followed by photochemical back-conversion upon irradiation with 340 nm.


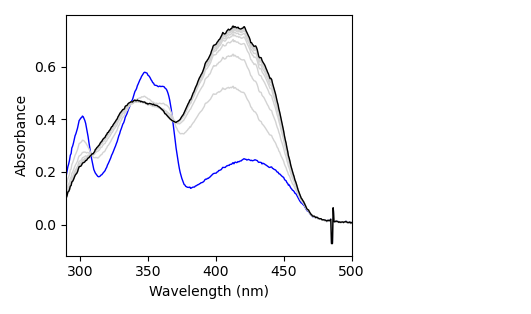

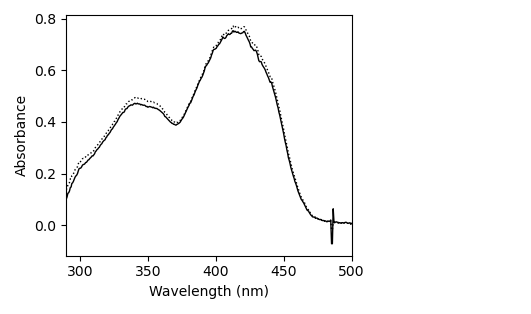


340 nm

**Figure S51.** Spectra of the photochemical back-conversion of **NBD-P2** in toluene. Left: The blue line shows the spectrum after conversion upon irradiation with 405 nm, and subsequent irradiation at 340 nm to recover the NBD-NBD isomer. Right: almost full recovery of the initial NBD-NBD form (dashed line) is achieved upon 340 nm conversion (black solid line).

**NBD-P2 in acetonitrile**


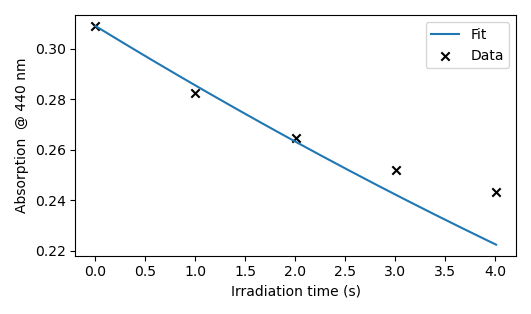


**Figure S52.** QY measurement for **NBD-P2** in acetonitrile (concentration = 1.43×10^-5^ M) upon 405 nm irradiation. Average QY = 3.5±0.3 % for the analysis wavelength of 440 nm.


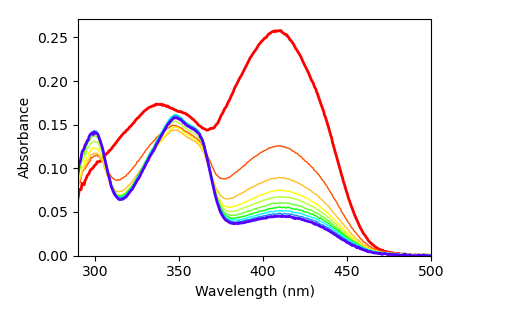


**Figure S53.** Conversion of **NBD-P2** in acetonitrile upon 405 nm irradiation showing conversion to a PSS after 600 seconds. Red: t = 0 s; Blue: t = 600 s.


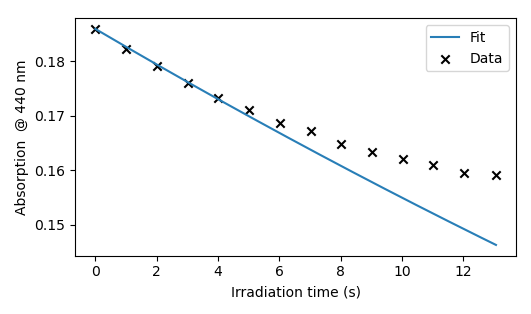
**Figure S54.** QY measurement for **NBD-P2** in acetonitrile (concentration = 1.43×10^-5^ M) upon 430 nm irradiation. Average QY = 4.1±0.1 % for the analysis wavelength of 440 nm.

**
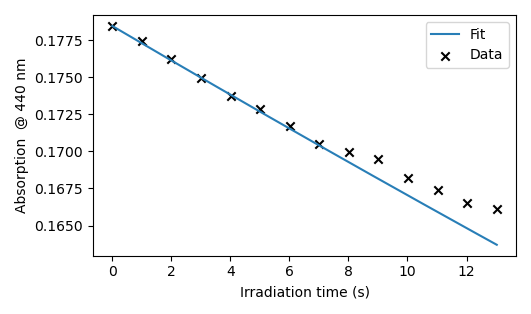
**

**Figure S55.** QY measurement for **NBD-P2** in acetonitrile (concentration = 1.43×10^-5^ M) upon 455 nm irradiation. Average QY = 0.77±0.01 % for the analysis wavelength of 440 nm.

**Figure S56.** Spectral change of **NBD-P2** in acetonitrile upon irradiation at 430 nm (Left) over 80 seconds and at 455 nm (Right) over 100 seconds.


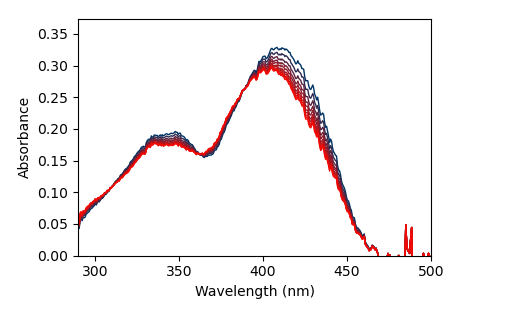

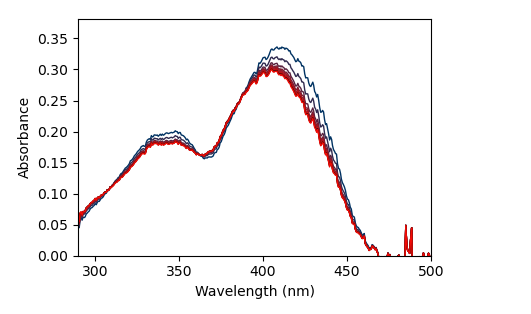


**
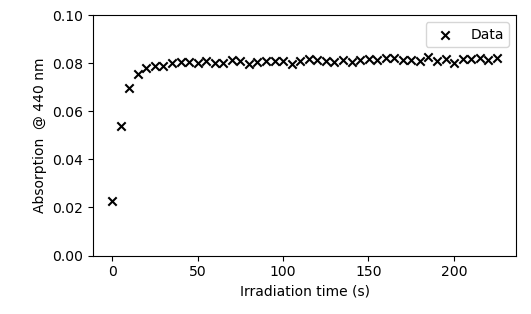
**

**Figure S57.** Photochemical backconversion of **NBD-P2** in acetonitrile, after first conversion with 405 nm LED, followed by photochemical back-conversion upon irradiation with 365 nm.


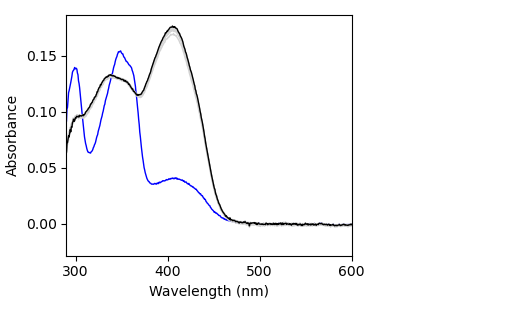


365 nm


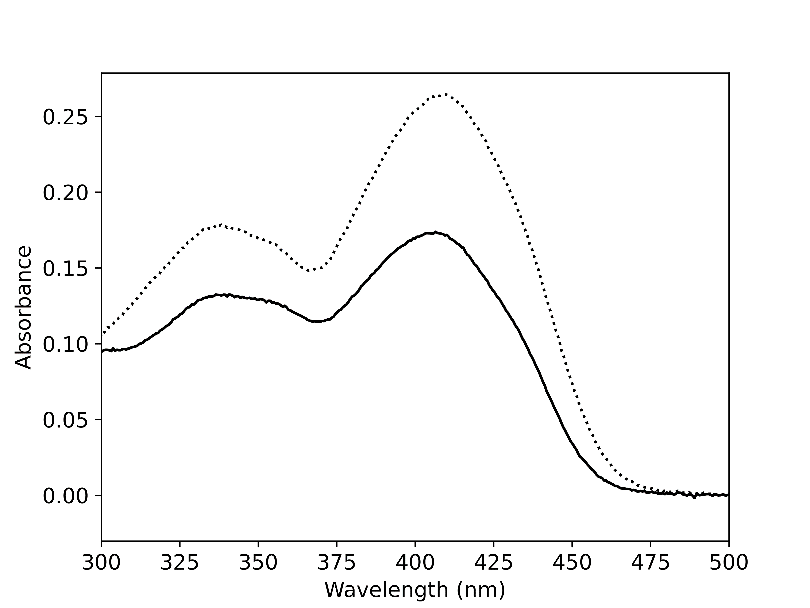


**Figure S58.** Spectra of the photochemical back-conversion of **NBD-P2** in acetonitrile. Left: The blue line shows the spectrum after conversion upon irradiation with 405 nm, and subsequent irradiation at 365 nm to recover the NBD-NBD isomer. Right: full recovery of the initial NBD-NBD form (dashed line) is not achieved upon 365 nm conversion, instead a photostationary state is reached (black solid line).


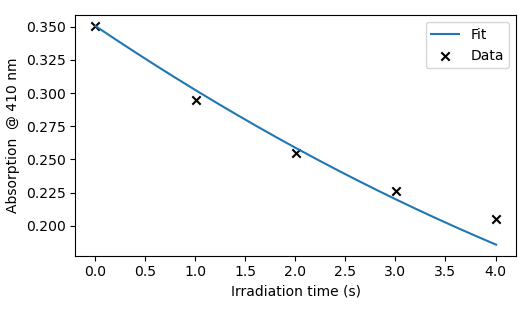
**NBD-O1 in toluene**

**Figure S59.** QY measurement for **NBD-O1** in toluene (concentration = 2.49×10^-5^ M) upon 405 nm irradiation. Average QY = 29±2 % for the analysis wavelength of 410 nm.


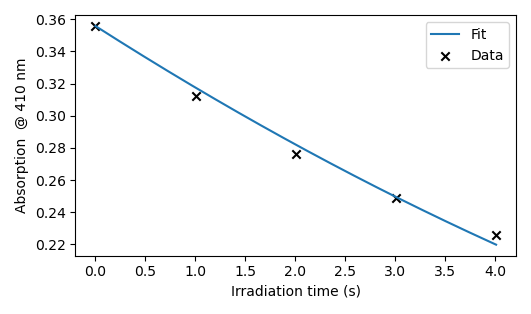


**Figure S60.** QY measurement for **NBD-O1** in toluene (concentration = 2.49×10^-5^ M) upon 365 nm irradiation. Average QY = 43±1 % for the analysis wavelength of 410 nm.


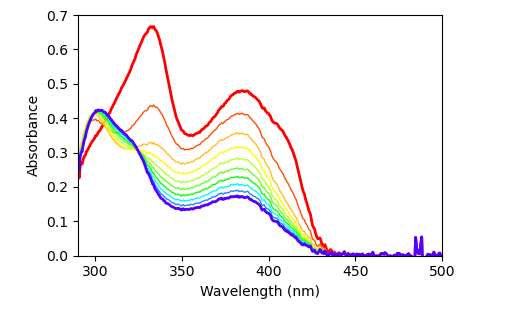

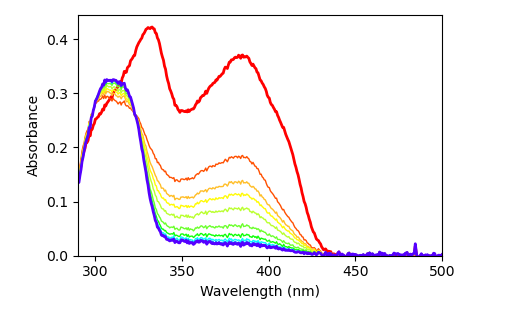


**Figure S61.** Conversion of **NBD-O1** in toluene upon 405 nm irradiation. Left: 1200 mA showing full conversion after 230 seconds; Right: 600 mA showing the first 44 seconds of conversion to highlight the spectral changes.


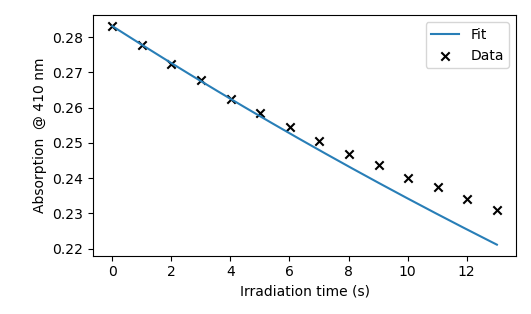


**Figure S62.** QY measurement for **NBD-O1** in toluene (concentration = 2.49×10^-5^ M) upon 430 nm irradiation. Average QY = 39±1 % for the analysis wavelength of 410 nm.


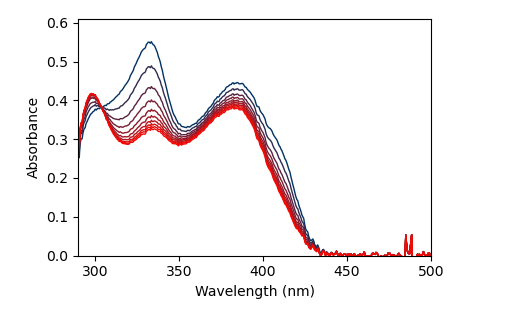


**Figure S63.** Spectral change of **NBD-O1** in toluene upon irradiation at 430 nm over 80 seconds.


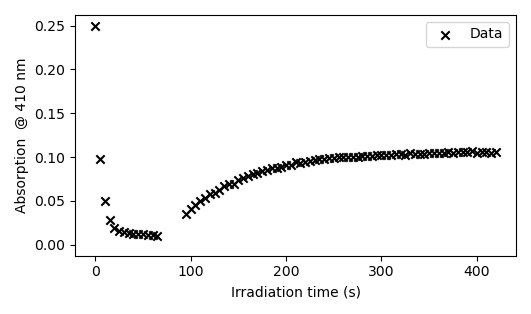


**Figure S64.** Irradiation of **NBD-O1** in toluene, first with 365 nm LED showing conversion (shaded grey area), followed by photochemical back-conversion upon irradiation with 308 nm.


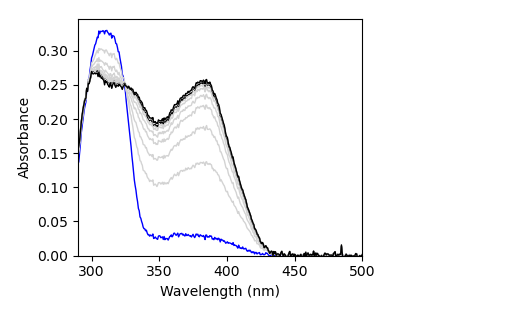

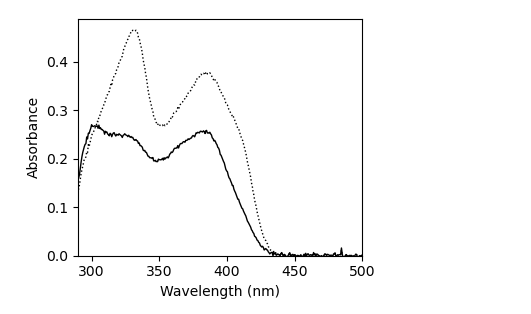


308 nm

**Figure S65.** Spectra of the photochemical back-conversion of **NBD-O1** in toluene. Left: The blue line shows the spectrum after conversion upon irradiation with 365 nm, and subsequent irradiation at 308 nm showing the photochemical back-conversion. Right: full recovery of the initial NBD-NBD form (dashed line) is not achieved upon 308 nm conversion, instead a photostationary state is reached (black solid line).

**NBD-O1 in acetonitrile**


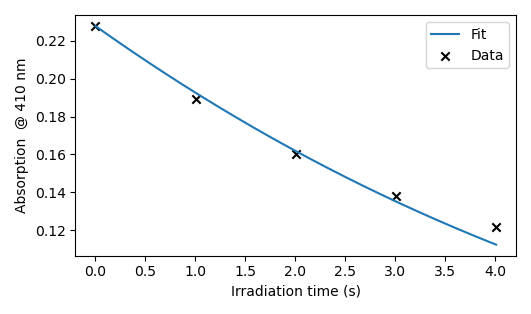


**Figure S66.** QY measurement for **NBD-O1** in acetonitrile (concentration = 3.16×10^-5^ M) upon 405 nm irradiation. Average QY = 48±1 % for the analysis wavelength of 410 nm.


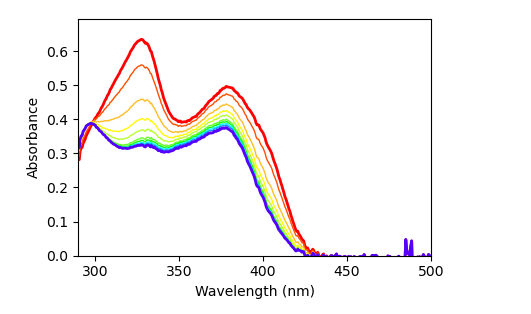

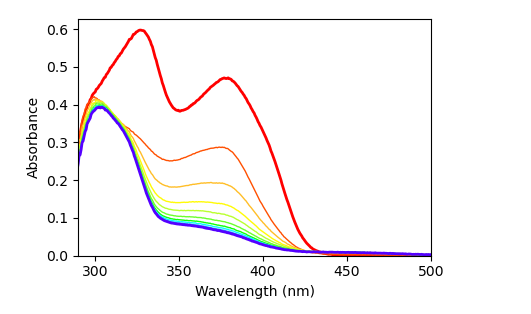


**Figure S67.** Conversion of **NBD-O1** in acetonitrile upon 405 nm irradiation. Left: 1200 mA showing full conversion after 60 seconds; Right: 600 mA showing the first 20 seconds of conversion to highlight the spectral changes.


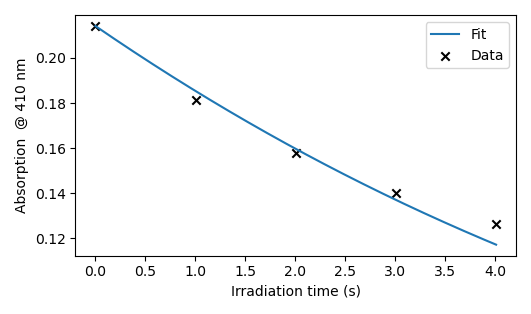


**Figure S68.** QY measurement for **NBD-O1** in acetonitrile (concentration = 3.16×10^-5^ M) upon 365 nm irradiation. Average QY = 63±2 % for the analysis wavelength of 410 nm.


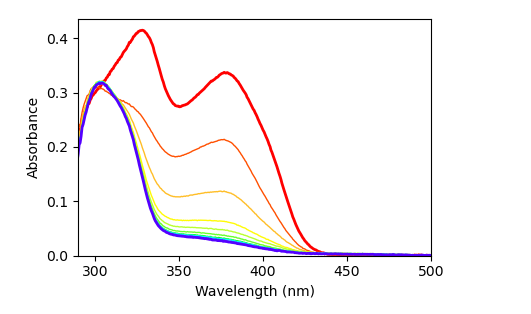


**Figure S69.** Conversion of **NBD-O1** in acetonitrile upon 365 nm irradiation showing full conversion after 30 seconds. Red: t = 0 s; Blue: t = 30 s.


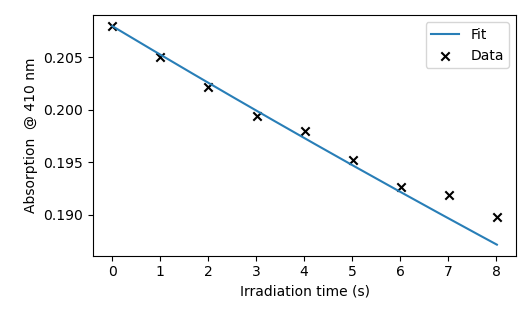


**Figure S70.** QY measurement for **NBD-O1** in acetonitrile (concentration = 3.16×10^-5^ M) upon 430 nm irradiation. Average QY = 86±3 % for the analysis wavelength of 410 nm.


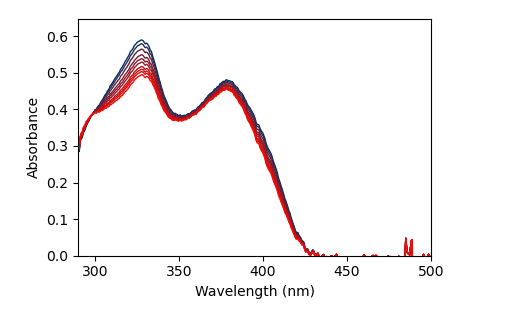


**Figure S71.** Spectral change of **NBD-O1** in acetonitrile upon irradiation at 430 nm over 50 seconds.

**NBD-O2 in toluene**


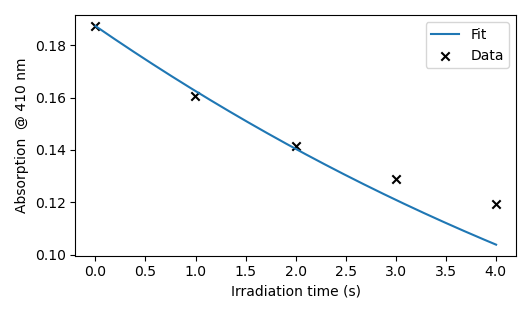


**Figure S72.** QY measurement for **NBD-O2** in toluene (concentration = 1.70×10^-5^ M) upon 405 nm irradiation. Average QY = 29±2 % for the analysis wavelength of 410 nm.


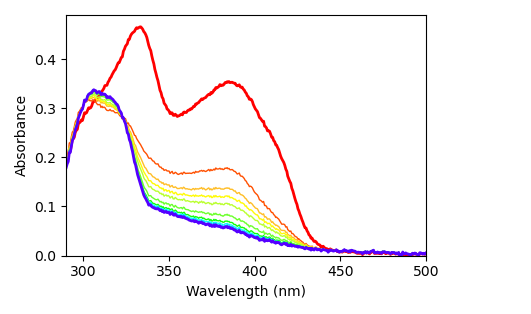

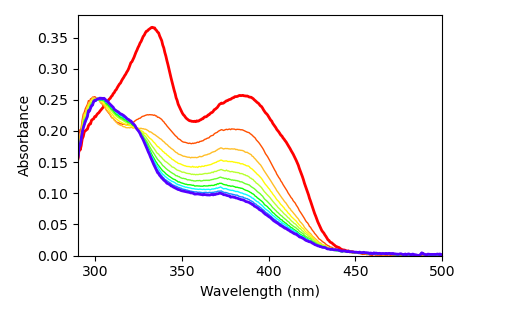


**Figure S73.** Conversion of **NBD-O2** in toluene upon 405 nm irradiation. Left: 1200 mA showing full conversion after 185 seconds; Right: 600 mA showing the first 54 seconds of conversion to highlight the spectral changes.


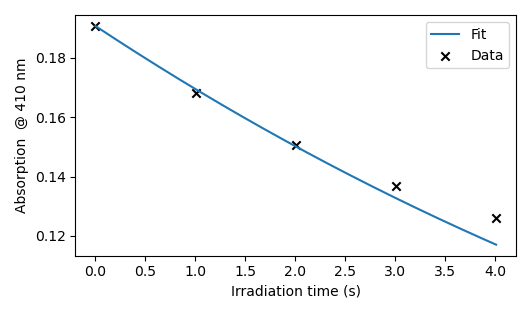


**Figure S74.** QY measurement for **NBD-O2** in toluene (concentration = 1.70×10^-5^ M) upon 365 nm irradiation. Average QY = 41±1 % for the analysis wavelength of 410 nm.


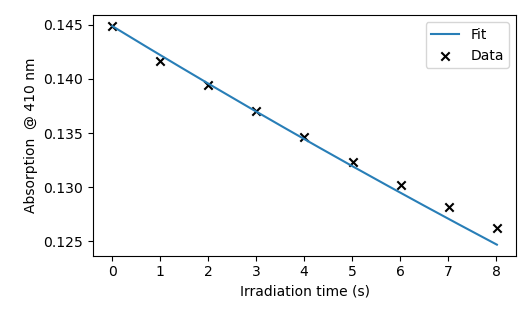


**Figure S75.** QY measurement for **NBD-O2** in toluene (concentration = 1.70×10^-5^ M) upon 430 nm irradiation. Average QY = 24±1 % for the analysis wavelength of 410 nm.


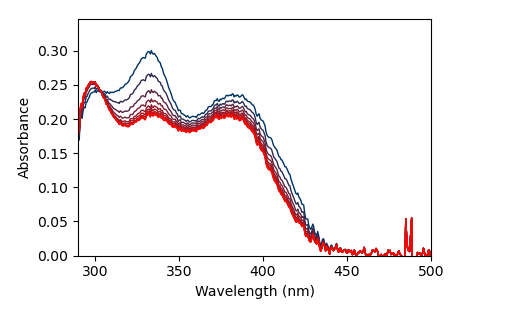


**Figure S76.** Spectral change of **NBD-O2** in toluene upon irradiation at 430 nm over 80 seconds.


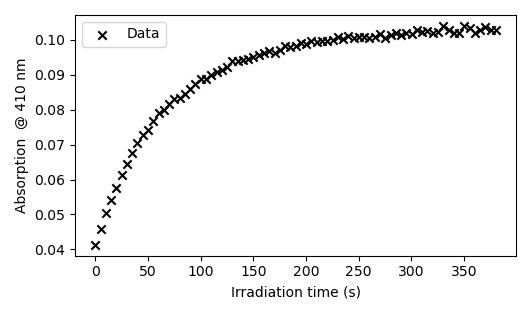


**Figure S77.** Photochemical backconversion of **NBD-O2** in toluene, after first conversion with 405 nm LED, followed by photochemical back-conversion upon irradiation with 308 nm.


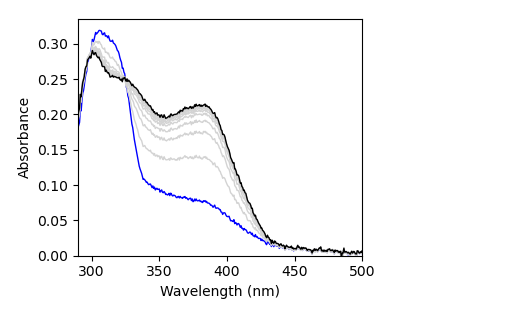
**Figure S78.** Spectra of the photochemical back-conversion of **NBD-O2** in toluene. Left: The blue line shows the spectrum after conversion upon irradiation with 405 nm, and subsequent irradiation at 308 nm showing the photochemical back-conversion. Right: full recovery of the initial NBD-NBD form (dashed line) is not achieved upon 308 nm conversion, instead a photostationary state is reached (black solid line).


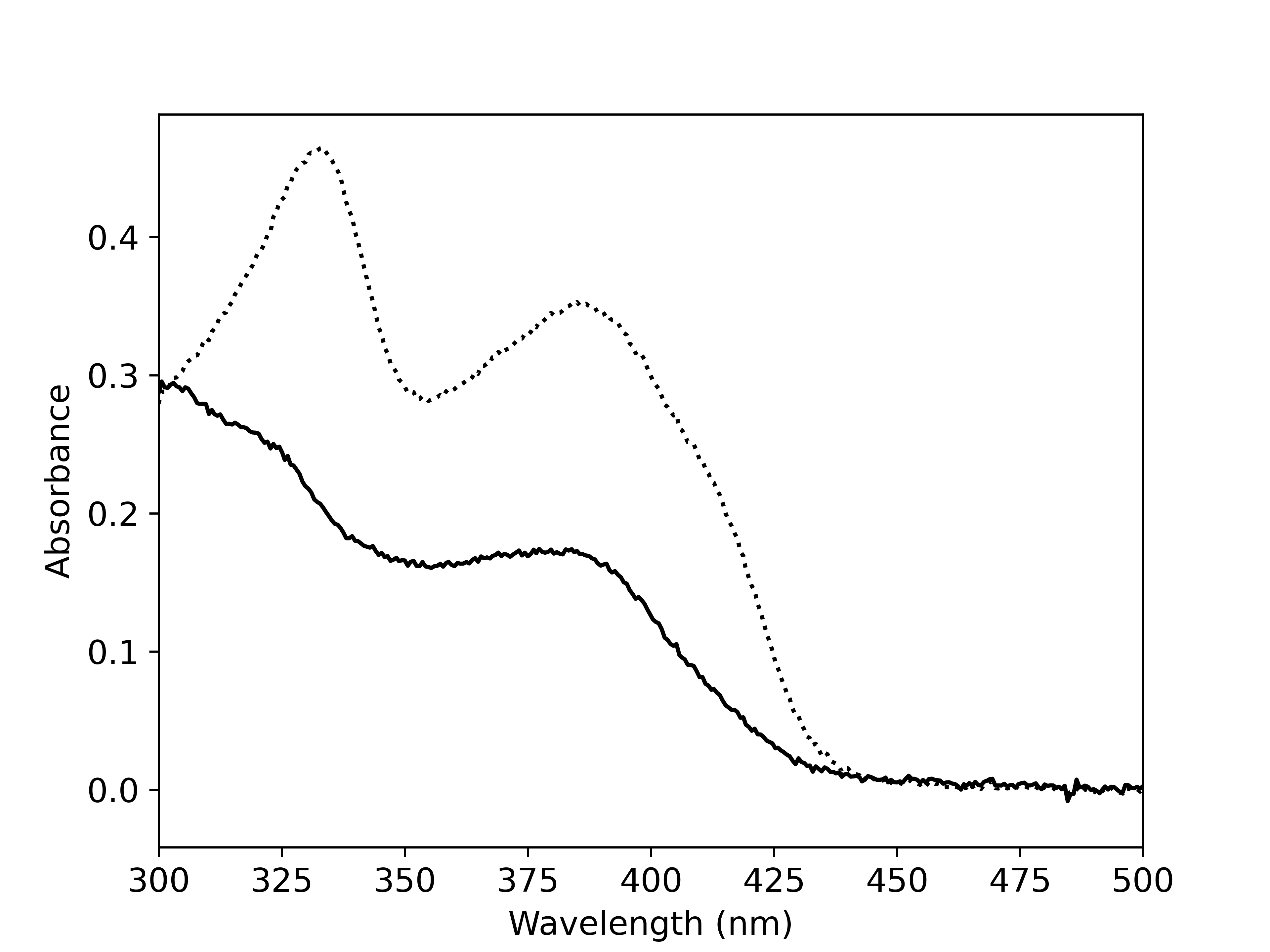


308 nm

**NBD-O2 in acetonitrile**


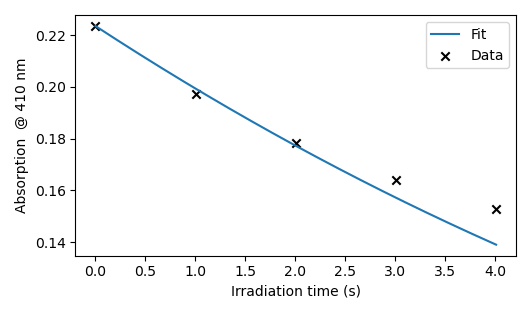


**Figure S79.** QY measurement for **NBD-O2** in acetonitrile (concentration = 2.86×10^-5^ M) upon 405 nm irradiation. Average QY = 35±1 % for the analysis wavelength of 410 nm.


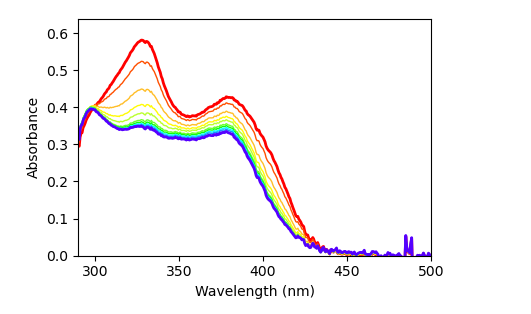

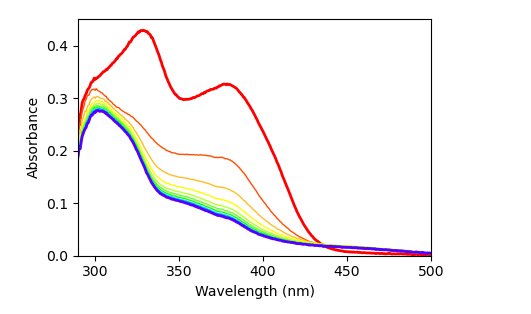


**Figure S80.** Conversion of **NBD-O2** in acetonitrile upon 405 nm irradiation. Left: 1200 mA showing full conversion after 80 seconds; Right: 600 mA showing the first 20 seconds of conversion to highlight the spectral changes.

**Figure S81.** QY measurement for **NBD-O2** in acetonitrile (concentration = 2.86×10^-5^ M) upon 365 nm irradiation. Average QY = 45±2 % for the analysis wavelength of 410 nm.

**Figure S82.** Conversion of **NBD-O2** in acetonitrile upon 365 nm irradiation. Left: 600 mA showing full conversion after 40 seconds; Right: 600 mA showing the first 30 seconds of conversion to highlight the spectral changes.

**Figure S83.** QY measurement for **NBD-O2** in acetonitrile (concentration = 2.86×10^-5^ M) upon 430 nm irradiation. Average QY = 30±2 % for the analysis wavelength of 410 nm.

**Figure S84.** Spectral change of **NBD-O2** in acetonitrile upon irradiation at 430 nm over 40 seconds.

**Table S1.** Quantum yields of photoisomerisation at various irradiation wavelengths

| **Dimer** | **Φ_i_ Toluene** | | | **Φ_i_ Acetonitrile** | | |
| --- | --- | --- | --- | --- | --- | --- |
|  | **405 nm** | **430 nm** | **455 nm** | **405 nm** | **430 nm** | **455 nm** |
| **NBD-P1** | 7% | 10% | 13% | 12% | 14% | 27% |
| **NBD-P2** | 1.5% | 1.9% | 0.4% | 3.5% | 4% | 0.8% |
| **NBD-O1** | 29% | 39% | - | 48% | 86% | - |
| **NBD-O2** | 29% | 24% | - | 35% | 30% | - |

# 6 Kinetics

**25 °C**

**35 °C**

**40 °C**

**65 °C**

**Figure S85.** Increase in absorbance (at 410 nm) of **NBD-P1** at four temperatures during the thermal backreaction.

R^2^ = -0.9982

R^2^ = -0.9983

**Figure S86.** Arrhenius (left) and Eyring (right) plots for **NBD-P1**.

**25 °C**

**35 °C**

**45 °C**

**55 °C**

**65 °C**

**Figure S87.** Increase in absorbance (at 410 nm) of **NBD-P2** at five temperatures during the thermal backreaction.

**Figure S88.** Arrhenius (left) and Eyring (right) plots for **NBD-P2**.

R^2^ = -0.9993

R^2^ = -0.9992

**25 °C**

**35 °C**

**45 °C**

**65 °C**

**Figure S89.** Increase in absorbance (at 380 nm) of **NBD-O1** at four temperatures during the thermal backreaction.

R^2^ = -0.9999

R^2^ = -0.9999

**Figure S90.** Arrhenius (left) and Eyring (right) plots for **NBD-O1**.

**25 °C**

**35 °C**

**45 °C**

**65 °C**

**Figure S91.** Increase in absorbance (at 390 nm) of **NBD-O2** at four temperatures during the thermal backreaction.

R^2^ = -0.9999

R^2^ = -0.9999

**Figure S92.** Arrhenius (left) and Eyring (right) plots for **NBD-O2**.

**Table S2.** Kinetic parameters for all NBD dimers

| **Dimer** | **A / s^-1^** | **E_a_ / kJ mol^-1^** | **ΔH / kJ mol^-1^** | **ΔS / J mol^-1^ K^-1^** | ***t*_1/2_ / h** |
| --- | --- | --- | --- | --- | --- |
| **NBD-P1** | 6.52e+12 | 96.5 | 93.9 | -8.48 | 2.41 |
| **NBD-P2** | 4.59e+11 | 89.4 | 86.7 | -30.5 | 1.90 |
| **NBD-O1** | 2.08e+12 | 93.9 | 91.2 | -18.0 | 2.61 |
| **NBD-O2** | 9.92e+11 | 92.3 | 89.7 | -24.1 | 2.90 |

# 7 Energy density (Differential Scanning Calorimetry)

The *para*-derivatives could not be fully converted in CD_2_Cl_2_, and so CDCl_3_ was chosen instead, despite the higher boiling point, which necessitated longer solvent removal times before measurement. The extent of conversion varied for each dimer depending on both the solvent and concentration used during irradiation.

**NBD-P1**

**Figure S93.** ^1^H-NMR spectra of **NBD-P1** in CDCl_3_ after irradiation at 405 nm before DSC and of samples 1-3 after heat release.

**Figure S94.** DSC spectrum of the heat release for **NBD-P1** Sample 1. Heating rate: 20 °C min^-1^. Sample amount: 1.44 mg. Integration area: 53.76 – 136.62 ºC. Heat release: 209.9 kJ kg^-1^. Corrected heat release: 244.1 kJ kg^-1^.

**Figure S95.** DSC spectrum of the heat release for **NBD-P1** Sample 2. Heating rate: 20 °C min^-1^. Sample amount: 3.13 mg. Integration area: 47.86 – 136.15 ºC. Heat release: 197.4 kJ kg^-1^. Corrected heat release: 229.5 kJ kg^-1^.

**Figure S96.** DSC spectrum of the heat release for **NBD-P1** Sample 3. Heating rate: 20 °C min^-1^. Sample amount: 1.37 mg. Integration area: 62.63 – 140.41 ºC. Heat release: 228.0 kJ kg^-1^. Corrected heat release: 265.1 kJ kg^-1^.

**NBD-P2**

**Figure S97.** ^1^H-NMR spectra of **NBD-P2** in CDCl_3_ after irradiation at 405 nm before DSC and of samples 1-3 after heat release.

**Figure S98.** DSC spectrum of the heat release for **NBD-P2** Sample 1. Heating rate: 20 °C min^-1^. Sample amount: 1.45 mg. Integration area: 29.54 – 107.23 ºC. Heat release: 156.7 kJ kg^-1^. Corrected heat release: 186.5 kJ kg^-1^.

**Figure S99.** DSC spectrum of the heat release for **NBD-P2** Sample 2. Heating rate: 20 °C min^-1^. Sample amount: 1.20 mg. Integration area: 29.59 – 107.45 ºC. Heat release: 151.8 kJ kg^-1^. Corrected heat release: 180.7 kJ kg^-1^.

**Figure S100.** DSC spectrum of the heat release for **NBD-P2** Sample 3. Heating rate: 20 °C min^-1^. Sample amount: 1.32 mg. Integration area: 30.45 – 103.70 ºC. Heat release: 132.4 kJ kg^-1^. Corrected heat release: 157.6 kJ kg^-1^.

**NBD-O1**

**Figure S101.** ^1^H-NMR spectra of **NBD-O1** in CD_2_Cl_2_ after irradiation at 405 nm before DSC and of samples 1-3 after heat release.

**Figure S102.** DSC spectrum of the heat release for **NBD-O1** Sample 1. Heating rate: 20 °C min^−1^. Sample amount: 1.97 mg. Integration area: 42.31 – 111.37 ºC. Heat release: 146.9 kJ kg^−1^. Corrected heat release: 178.1 kJ kg^−1^.

**Figure S103.** DSC spectrum of the heat release for **NBD-O1** Sample 2. Heating rate: 20 °C min^‑1^. Sample amount: 3.13 mg. Integration area: 54.81 – 110.10 ºC. Heat release: 144.4 kJ kg^-1^. Corrected heat release: 175.0 kJ kg^-1^.

**Figure S104.** DSC spectrum of the heat release for **NBD-O1** Sample 3. Heating rate: 20 °C min^‑1^. Sample amount: 1.71 mg. Integration area: 46.49 – 109.88 ºC. Heat release: 109.9 kJ kg^-1^. Corrected heat release: 133.2 kJ kg^-1^.

**NBD-O2**

**Figure S105.** ^1^H-NMR spectra of **NBD-O2** in CD_2_Cl_2_ after irradiation at 405 nm before DSC and of samples 1-3 after heat release.

**Figure S106.** DSC spectrum of the heat release for **NBD-O2** Sample 1. Heating rate: 20 °C min^‑1^. Sample amount: 1.05 mg. Integration area: 39.47 – 120.54 ºC. Heat release: 101.0 kJ kg^-1^. Corrected heat release: 123.2 kJ kg^-1^.

**Figure S107.** DSC spectrum of the heat release for **NBD-O2** Sample 2. Heating rate: 20 °C min^‑1^. Sample amount: 1.31 mg. Integration area: 37.84 – 126.66 ºC. Heat release: 138.3 kJ kg^-1^. Corrected heat release: 168.7 kJ kg^-1^.

**Figure S108.** DSC spectrum of the heat release for **NBD-O2** Sample 3. Heating rate: 20 °C min^‑1^. Sample amount: 1.68 mg. Integration area: 44.87 – 117.88 ºC. Heat release: 104.5 kJ kg^-1^. Corrected heat release: 127.4 kJ kg^-1^.

# 8 Fluorescence

**Table S3.** Absorbance and emission maxima and lifetimes for the most fluorescent dimers **NBD-P1** and **NBD-P2** in methylcyclohexane (MCH), toluene, MeTHF and acetonitrile.

| **Dimer** | **Solvent** | **λ_max_ Abs / nm** | **λ_max_ Em / nm** | **τ / ns** |
| --- | --- | --- | --- | --- |
| **NBD-P1** | MCH | 407 | 448 | 1.70 |
|  | Toluene | 411 | 465 | 1.78 |
|  | MeTHF | 405 | 465 | 1.77 |
|  | MeCN | 401 | 476 | 1.92 |
| **NBD-P2** | MCH | 414 | 461 | 1.70 |
|  | Toluene | 417 | 470 | 1.78 |
|  | MeTHF | 412 | 469 | 1.82 |
|  | MeCN | 409 | 478 | 1.71 |

**Table S4.** Photoluminescence quantum yields (measured via the absolute method) for all dimers in methylcyclohexane (MCH), toluene, MeTHF and acetonitrile.

| **Dimer** | **Φ_f_ MCH** | **Φ_f_ toluene** | **Φ_f_ MeTHF** | **Φ_f_ MeCN** |
| --- | --- | --- | --- | --- |
| **NBD-P1** | 48% | 51% | 44% | 29% |
| **NBD-P2** | 53% | 56% | 60% | 44% |
| **NBD-O1** | 0 | 0 | 4% | 1% |
| **NBD-O2** | 2% | 1% | 2% | 1% |

**Figure S109.** Absorbance and emission spectra of **NBD-O1** and **NBD-O2** in methylcyclohexane, toluene, MeTHF and acetonitrile at 20 µM. The sharp peak at 455 nm is due to the Raman solvent peak (λ_excitation_ = 400 nm) as the dimers are very weakly emissive.

**Figure S110.** Normalised absorbance and emission spectra of **NBD-P1** and **NBD-P2** in methylcyclohexane, toluene, MeTHF and acetonitrile at 20 µM.

**Figure S111.** Absorbance and excitation spectra (normalised) for **NBD-O1** in various solvents for λ_em_ = 425 nm (MCH), 435 nm (toluene), 450 nm (MeTHF) and 465 nm (MeCN).

**Figure S112.** Absorbance and excitation spectra (normalised) for **NBD-O2** in various solvents for λ_em_ = 450 nm (MCH), 455 nm (toluene), 460 nm (MeTHF) and 485 nm (MeCN).

**Figure S113.** Absorbance and excitation spectra (normalised) for **NBD-P1** in various solvents for λ_em_ = 450 nm (MCH), 465 nm (toluene), 465 nm (MeTHF) and 480 nm (MeCN).

**Figure S114.** Absorbance and excitation spectra (normalised) for **NBD-P2** in various solvents for λ_em_ = 460 nm (MCH), 470 nm (toluene), 470 nm (MeTHF) and 480 nm (MeCN).

# 9 Solar simulator measurements

The solar conversion efficiency (SCE) experiments were performed using a flow-based setup in a solar simulator. The solar simulator was an ISOSun Solar Simulator from InfinityPV. A Vapourtec V10 peristaltic pump was used to flow the solution through ETFE tubing and into the microfluidic chip (see **Figure S115**). An Avantes spectrophotometer (AvaSpec-ULS2048CL-EVO) and an Avantes (AvaLight-D(H)-S) deuterium lamp were used to generate absorbance spectra of the sample to continuously monitor the photoconversion. In the case of the higher concentration (0.01 M) experiment, samples were taken and diluted 100-fold before measurement of absorbance spectra. Optical fibre cables from Thorlabs were used to connect the light source and the spectrophotometer to a 2.5 mm path length flow cell from FIA Lab (FIA-ZSMA-MK-2.5-PE). The concentrations of the samples were prepared to obtain an absorbance below 1 at the path length of 2.5 mm. A similar setup has been described by Wang *et al*.^8^

To evaluate the experimental solar conversion efficiency (eSCE, $\eta_{eff})$ values, we used the following equation:

$$\eta_{eff}=\frac{ṅ_{parent}*\alpha_{conversion}*{\Delta H}_{storage}}{S*E_{AM1.5G}}$$

where $ṅ_{parent}$ is the flowrate of the parent molecule in mL min^-1^, $\alpha_{conversion}$ is the conversion percentage of the dimer, Δ*H* is the storage energy (of the fully switched dimer), *S* is the effective irradiated area of the chip in m^2^ and E_AM1.5G_ is the power of the solar simulator. In evaluating the SCE, we used the theoretical Δ*H* obtained from the DFT methods described in **Section 11**. The theoretical value was used because the measured heat release from the DSC experiments was largely underestimated due to rapid thermal back-conversion. The solar conversion efficiencies are higher at higher flow rates despite a lower conversion percentage.

**Figure S115.** Photo of the liquid chip flow setup used for solar conversion efficiency measurements in the solar simulator.

**Figure S116.** Initial spectra and converted spectra of **NBD-O1** in toluene at 1.88×10^-4^ M in the solar simulator at various flowrates.

**Figure S117.** Initial spectra and converted spectra of **NBD-O2** in toluene at 2.13×10^-4^ M in the solar simulator at various flowrates.

**Figure S118.** Initial spectra and converted spectra of **NBD-O1** in acetonitrile at 1.94×10^-4^ M in the solar simulator at various flowrates.

**Figure S119.** Initial spectra and converted spectra of **NBD-O2** in acetonitrile at 1.90×10^-4^ M in the solar simulator at various flowrates.

**Figure S120.** Initial spectra and converted spectra of **NBD-O2** in acetonitrile at 0.01 M in the solar simulator at various flowrates.

The theoretical conversion efficiency limit was also evaluated to simulate the solar conversion efficiency at higher concentrations in the solar spectrum AM1.5G. The spectral overlap was determined using the AM1.5G spectrum and the spectra of two samples with different concentrations. The high concentration UV-Vis spectra were of saturated solutions, which can explain the baseline offset.

**Figure S121.** Absorption spectra for **NBD-O1** at low concentration (1×10^-4^ M) and high concentration (0.03 M) in toluene and acetonitrile compared to the AM1.5G solar spectrum.

**Figure S122.** Absorption spectra for **NBD-O2** at low concentration (1×10^-4^ M) and high concentration (0.03 M) in toluene and acetonitrile compared to the AM1.5G solar spectrum

The theoretical solar conversion efficiency (tSCE, $\eta_{limit}$) limit was then calculated using the following equation:

$$\eta_{limit}=\frac{1\int_{0}^{\lambda_{onset}} \frac{E_{AM1.5G}\left( \lambda\right)\cdot\left( 1-T\left( c \right) \right)\cdot\phi_{iso}\cdot\Delta H_{storage}}{h\nu\cdot N_{A}}\cdot d\lambda}{2\int E_{AM1.5G}\left( \lambda\right)\cdot d\lambda} ,$$

where E_AM1.5G_ is the solar spectrum measured by Gueymard *et al*.,^9^ T(c) is the attenuation, Φ_iso_ is the photoconversion quantum yield, Δ*H*_storage_ is the storage energy, *h*ν is the photon energy, and N_A_ is Avogadro’s number. Note the factor of two as there are two photoswitches per molecule. The theoretical solar conversion efficiencies were calculated for **NBD-O1** and **NBD-O2** using the low and high concentration UV-Vis spectra to compare to our low-concentration experiment and to simulate the gain in efficiency in a higher concentration. A cut-off of 500 nm was chosen as we do not know with certainty the quantum yield of conversion at this and longer wavelengths. **NBD-O1** and **NBD-O2** follow very similar trends in predicted SCEs due to their similar absorbance profiles. The efficiency differences are largely due to the difference in quantum yields, where $\phi_{c}$ (**NBD-O1**) = 0.3 and $\phi_{c}$ (**NBD-O2**) = 0.4. The quantum yields were set to be the highest measured quantum yield and assumed to be consistent across the absorbing spectral overlap.

**Figure S123.** Calculated solar conversion efficiency limits for **NBD-O1** at low concentration (1×10^-4^ M) and high concentration (0.03 M) in toluene and acetonitrile in a solar simulator and the AM1.5G solar spectrum.

**Figure S124.** Calculated solar conversion efficiency limits for **NBD-O2** at low concentration (1×10^-4^ M) and high concentration (0.03 M) in toluene and acetonitrile in a solar simulator and the AM1.5G solar spectrum.

# 10 Catalytic back conversion and macroscopic heat release

The CoPc@C catalyst was prepared according to a reported procedure.^10^

**Figure S125.** NMR spectra of **NBD-O2** in CDCl_3_: before irradiation in the NBD-NBD form, after irradiation with a 405 nm LED to convert to the QC-QC form, after addition of CoPc catalyst and shaking for 5 minutes, and full back-conversion to the NBD-NBD form 10 minutes after addition of the catalyst.

**A**

**B**

**Figure S126.** A) UV-Vis spectra of **NBD-O2** in toluene (9×10^-5^ M) following the catalytic back conversion of the QC-QC form to NBD-NBD upon the addition of CoPc (0.5 mg) at various time intervals as given in the legend. The compound was first converted to the QC-QC form upon irradiation with 365 nm. B) Increase in absorbance at 390 nm upon catalytic back conversion of **NBD-O2** with CoPc in toluene at 25 °C. The rate constant for the catalytic back conversion from QC-QC to NBD-NBD is *k*_cat_ = 1.68×10^-3^ s^-1^. The absorption wavelength of 390 nm was chosen to match with that used for the determination of the rate constant of the thermal back reaction as shown in **Figure S90**.

**Photoconversion of NBDs under flow conditions**

In order to convert the selected compound **NBD-O2** to its QC-QC isomer ready for the heat release experiment, photoconversions under flow conditions were carried out using Vapourtec R-series flow systems equipped with a photo-irradiation unit, a Vapourtec UV-150 photochemical reactor. For the photoconversion, a 365 nm LED was chosen (Vapourtec 365 nm Gen-2, 16 W radiant power) for the experiment, and its power was set to 100%. The volume of the sample loop for the photoirradiation was 10 mL. Various residence times were investigated; however, the best result so far was achieved for a residence time of t_res_ = 15 min.

**Figure S127.** General scheme for photoconversion of NBD derivatives to QC derivatives under flow conditions in this study.

We carried out a test experiment for a 0.1 M solution in toluene (2.93 mL) with a residence time of 15 min. ^1^H NMR spectra were recorded before and after the experiment to determine the conversion percentage. Despite the overlapping peaks with the toluene, the peaks corresponding to the NBD-NBD isomer disappeared after 15 mins of irradiation at 365 nm (**Figure S203**), hence we conclude that the 0.1 M sample was fully converted.

**Figure S128.** ^1^H NMR of 0.1 M **NBD-O2** in CDCl_3_ before and after flow irradiation at 365 nm. Note there is residual toluene from the conversion experiment.

**Figure S129.** Scheme for photoconversion of **NBD-O2** (0.5 M) under flow conditions in toluene.

The sample for the macroscopic heat release was prepared similarly. The solution of **NBD-O2** in toluene (0.5 M, 1.96 mL) was pumped through the photoreactor with a flow rate of 0.667 mL/min. ^1^H NMR spectra were recorded before and after the experiment to determine the conversion percentage. Even after two photoconversion experiments in flow, the composition of the sample was the following: NBD-NBD 45%, NBD-QC 38%, QC-QC 17%. Nevertheless, this sample was used for the experiment of the macroscopic heat release.

NBD-QC

NBD-QC

NBD-NBD

QC-QC

**Figure S130.** ^1^H NMR spectra of 0.5 M **NBD-O2** in CDCl_3_ before conversion, after 365 nm irradiation in flow, and after the macroscopic heat release experiment. Composition of converted sample before the heat release experiment: 45% NBD-NBD, 38% NBD-QC and 17% QC-QC. Composition of sample after the heat release experiment: 68% NBD-NBD, 32% NBD-QC and 0% QC-QC.

The setup for the macroscopic heat release is depicted schematically in **Figure S206**, along with a photo. A similar setup has been published previously.^10^ In the central part of the setup, inside the vacuum chamber is a 2.5 cm Teflon tubing (1.27 mm inner diameter) with a loading length of 1.5 cm with cotton to block each side, from which 1 cm corresponds to the loaded catalyst CoPc@C (4 mg), which was reported previously. The 0.5 M solution in toluene (1.9 mL) was pumped through the setup with a flow rate of 5 mL h^-1^ (84 mL min^-1^). The vacuum varied in the range of 10^-4^ to 10^-3^ mbar during the experiment. We observed a heat release of 5.78 ⁰C. Due to pressure jumps during the experiment, we did not use the total volume of 1.9 mL for the experiment. The small spikes in the heat release graph (**Figure S207**) can be attributed to the pressure jumps of the pump. The initial working temperature was 17.29 ºC before the solution hit the catalyst. The temperature increased to 23.07 ºC immediately when the solution passed the catalytic center. ^1^H NMR spectra were recorded before and after the experiment to determine the conversion percentage (**Figure S205**). After the heat release experiment, the composition of the sample was the following: NBD-NBD 68%, NBD-QC 32%, QC-QC 0%.

**Figure S131.** Left: schematic depiction of the vacuum setup used in this study. Right: photo.

**Figure S132.** Heat release experiment of **NBD-O2** in toluene, showing a ΔT of 5.78 ⁰C.

An estimation of the macroscopic heat release was calculated based on the theoretical Δ*H*_storage_ value, previous experiments with a CoPc catalyst,^10^ and the following equation for ΔT:

$$\Delta T = \frac{c\cdot M_{w}\cdot\Delta H_{storage}}{c\cdot M_{w}\cdot C_{p}(PI)+ \rho_{solvent}\cdot C_{p}(solvent)}$$

where c and M_w_ represent the concentration of the reactive photoswitch species and molecular weight, respectively; Δ*H*_storage_ is the DSC-measured energy storage capacity of the photoswitch couple in J g^-1^; *C_p_* (PI) is the specific heat capacity of the photoisomer in J g^-1^ K^-1^; and *ρ*_solvent_ and *C_p_* (solvent) correspond to the volumetric mass density in g L^-1^ and the specific heat capacity in J g^-1^ K^-1^ of the solvent (here toluene, 867 g L^-1^ and 1.7 J g^-1^ K^-1^). The specific heat capacity *C_p_* (PI) herein is experimentally determined based on a single experiment, resulting in a ΔT of 5.78 ⁰C. This value for the heat capacity *C_p_* (PI) is also used for the theoretical prediction of the heat release based on the calculated energy storage density. It is to be noted, that the assumption made here is that the sample containing only reactive photoisomer species (i.e. NBD-QC and QC-QC) contributes to ΔH_storage_, with NBD-QC contributing half of that of the fully-converted QC-QC. As the sample was not full converted before the heat release experiment, and based on the NMR before and after conversion, we assumed that only 20% of reactive photoisomer species was present in the sample, resulting in the heat release. Thus, the initial 0.5 M solution contained 0.1 M reactive photoisomer species. Based on this, we estimated a theoretical heat release with the theoretically predicted energy storage density of 303.9 kJ kg^-1^ to result in a ΔT of 12.6 ⁰C for a 0.1 M reactive photoisomer species.

# 11 Computational details

The global optimiser algorithm (GOAT) using the semi-empirical method GFN2-xTB in Orca 6.0 was used to determine the structure of the lowest energy conformer of each compound.

The density functional theory (DFT) functional and basis set wB97X-D3BJ / aug-cc-pVDZ were used to further optimise the structure and calculate the ground-state energy. The storage energy was calculated as the energy difference between the NBD-NBD state and the QC-QC state. The energy levels of the HOMO and the LUMO of the NBD-NBD ground-state were calculated using the TD-DFT functional and basis set B3LYP / def2-TZVP. The orbitals were visualised in Avogadro after using the orca_plot and orca_2mkl functions of Orca 6.0 to generate the cube files.

**LUMO+1**

**LUMO**

**HOMO**

**Figure S133.** Molecular structure and frontier orbital plots for **NBD-P1**. The HOMO$\to$LUMO is the transition with the highest oscillator strength (445.6 nm, *f* = 1.5827) whilst the HOMO$\to$LUMO+1 transition is negligble (388.2 nm, *f* = 0.000001).

**LUMO+1**

**LUMO**

**HOMO**

**Figure S134.** Molecular structure and frontier orbital plots for **NBD-P2**. The HOMO$\to$LUMO is the transition with the highest oscillator strength (474.9 nm, *f* = 0.9227) whilst the HOMO$\to$LUMO+1 transition is negligble (396.7 nm, *f* = 0.00009).

**HOMO**

**LUMO**

**LUMO+1**

**Figure S135.** Molecular structure and frontier orbital plots for **NBD-O1**. The HOMO$\to$LUMO+1 is the transition with the highest oscillator strength (404.5 nm, *f* = 0.1894) whilst the HOMO$\to$LUMO transition is negligble (417.0 nm, *f* = 0.0069).

**LUMO+1**

**LUMO**

**HOMO**

**Figure S136.** Molecular structure and frontier orbital plots for **NBD-O2**. The HOMO$\to$LUMO+1 is the transition with the highest oscillator strength (406.9 nm, *f* = 0.1844) whilst the HOMO$\to$LUMO transition is negligble (418.8 nm, *f* = 0.0098).

**Figure S137.** Calculated absorption spectra of the isomers of **NBD-P1**.

**Figure S138.** Calculated absorption spectra of the isomers of **NBD-O1**.

**Cartesian coordinates**

**NBD-P1 (NBD-NBD form)**

Atom x y z

C -0.70956 -1.38618 3.35900

O -1.37547 -1.07588 2.15817

C -0.64201 -0.60467 1.11597

C 0.72606 -0.40328 1.12989

C 1.39084 0.08722 0.00216

C 2.77774 0.27812 0.05372

C 3.97681 0.43250 0.15084

C 5.34696 0.60259 0.23035

C 6.18058 0.36405 1.27636

C 5.86443 -0.10457 2.54486

N 5.61718 -0.50071 3.59679

C 7.58335 0.76175 0.82156

C 7.51883 2.26787 0.62814

C 6.70555 2.49905 -0.39286

C 6.21732 1.15166 -0.89788

C 7.48524 0.31589 -0.64802

C 0.67466 0.38677 -1.17765

O 1.40823 0.85756 -2.22024

C 0.74123 1.17398 -3.41869

C -0.69330 0.18483 -1.19191

C -1.35806 -0.30535 -0.06410

C -2.74480 -0.49752 -0.11592

C -3.94432 -0.65202 -0.20669

C -5.31253 -0.83442 -0.29214

C -6.14429 -0.59939 -1.34037

C -5.82989 -0.10316 -2.59884

N -5.58007 0.29859 -3.64802

C -7.55598 -0.94154 -0.86851

C -7.87136 0.09134 0.20095

C -7.05952 -0.13993 1.22307

C -6.19243 -1.33152 0.85284

C -7.21402 -2.14025 0.03389

H -0.22013 -0.50568 3.78674

H 0.03084 -2.17921 3.21490

H -1.48330 -1.73510 4.04070

H 1.31851 -0.61679 2.00544

H 8.40014 0.38086 1.42792

H 8.02300 2.96979 1.26420

H 6.37859 3.43691 -0.79874

H 5.76503 1.13393 -1.88485

H 8.33476 0.63773 -1.24992

H 7.33169 -0.75690 -0.76933

H 0.00007 1.96539 -3.26985

H 1.51412 1.52748 -4.09906

H 0.25244 0.29549 -3.85140

H -1.28581 0.39755 -2.06761

H -8.29503 -1.07440 -1.65338

H -8.59648 0.87234 0.07571

H -6.95469 0.40543 2.14111

H -5.66516 -1.82376 1.66445

H -8.06110 -2.48390 0.62713

H -6.77473 -2.98059 -0.50462

**NBD-P1 (NBD-QC form)**

Atom x y z

C 0.49857 -3.58644 -0.05980

O 1.26605 -2.40696 -0.05591

C 0.61558 -1.21257 -0.05382

C -0.75988 -1.05529 -0.05272

C -1.33485 0.21567 -0.04969

C -2.73623 0.33261 -0.04862

C -3.94118 0.41134 -0.03840

C -5.34435 0.44016 -0.04126

C -6.33316 1.15895 -0.94780

C -7.22707 -0.07686 -0.97323

C -7.58864 -0.31950 0.45945

C -7.38629 0.92753 1.27961

C -6.24321 1.52906 0.50448

C -6.25459 -0.81481 -0.06513

C -5.81243 -2.14200 -0.09235

N -5.43213 -3.22479 -0.11798

C -0.52375 1.36871 -0.04836

O -1.17953 2.56336 -0.04596

C -0.41246 3.74264 -0.04548

C 0.84950 1.21364 -0.04979

C 1.42829 -0.05979 -0.05255

C 2.82435 -0.17804 -0.05351

C 4.03621 -0.22779 -0.06069

C 5.41605 -0.31640 -0.05685

C 6.87859 -1.64808 -1.44239

C 7.78171 -0.67757 -1.44424

C 7.71249 0.00530 -0.08853

C 7.39095 -1.21546 0.79123

C 6.19583 -1.62943 -0.08537

C 6.34142 0.67865 -0.05692

C 6.13689 2.05167 -0.07192

N 5.98345 3.19254 -0.07773

H -0.13048 -3.65486 -0.95287

H 1.21808 -4.40357 -0.06250

H -0.13049 -3.66073 0.83285

H -1.42423 -1.90502 -0.05390

H -6.13765 1.81357 -1.77675

H -7.75767 -0.45608 -1.82773

H -8.35107 -1.03841 0.71687

H -7.11485 0.70523 2.31249

H -8.27104 1.56655 1.27331

H -5.80394 2.46911 0.79754

H 0.21600 3.81509 -0.93885

H -1.13193 4.55983 -0.04421

H 0.21733 3.81345 0.84710

H 1.51384 2.06324 -0.04906

H 6.60300 -2.31746 -2.23444

H 8.42887 -0.35710 -2.23803

H 8.55307 0.63685 0.18474

H 8.18979 -1.95678 0.80295

H 7.11312 -0.95561 1.81325

H 5.62774 -2.51345 0.18832

**NBD-P1 (QC-QC form)**

Atom x y z

C 0.59454 1.42941 3.39757

O 1.31848 0.91953 2.30507

C 0.61883 0.44114 1.23580

C -0.76105 0.41561 1.13514

C -1.38614 -0.09479 -0.00325

C -2.79208 -0.10209 -0.06791

C -3.99855 -0.09272 -0.10738

C -5.40289 -0.06713 -0.10186

C -6.42834 -1.16398 -0.34729

C -7.28606 -0.63630 0.79852

C -7.61489 0.77343 0.41572

C -7.43518 0.98017 -1.06535

C -6.32359 -0.00868 -1.30151

C -6.27611 0.47075 1.06129

C -5.80096 0.99622 2.26770

N -5.40180 1.41554 3.25907

C -0.62092 -0.59589 -1.07284

O -1.32020 -1.07436 -2.14228

C -0.59631 -1.58737 -3.23334

C 0.75905 -0.57152 -0.97150

C 1.38409 -0.06005 0.16629

C 2.78993 -0.05286 0.23146

C 3.99673 -0.04716 0.26004

C 5.40089 -0.08028 0.25465

C 6.39724 -0.63992 1.25880

C 7.25001 -1.19530 0.12243

C 7.62753 0.00063 -0.69560

C 7.48009 1.26570 0.10842

C 6.34365 0.83890 1.00047

C 6.26917 -0.64245 -0.90082

C 5.78776 -1.19921 -2.09054

N 5.37915 -1.65801 -3.06034

H -0.01981 2.28845 3.10909

H -0.04413 0.66439 3.85080

H 1.34270 1.74877 4.12142

H -1.39190 0.78739 1.92714

H -6.26858 -2.19752 -0.59269

H -7.82051 -1.23249 1.51590

H -8.35112 1.33361 0.97134

H -7.13940 2.00160 -1.30878

H -8.33849 0.72807 -1.62383

H -5.90753 -0.15515 -2.28540

H 0.04294 -0.82392 -3.68843

H 0.01743 -2.44610 -2.94271

H -1.34446 -1.90815 -3.95657

H 1.38882 -0.94409 -1.76398

H 6.21213 -1.12006 2.20175

H 7.75392 -2.14490 0.11523

H 8.37018 -0.08149 -1.47427

H 8.38506 1.49652 0.67332

H 7.21925 2.12380 -0.51263

H 5.94075 1.50415 1.74726

**NBD-P2 (NBD-NBD form)**

Atom x y z

C 3.92064 2.77880 0.18091

C 4.42853 1.41355 0.62759

C 3.34111 0.34894 0.51595

C 3.84312 -1.02766 0.94441

C 2.75704 -2.10473 0.90001

C 2.43235 -2.58362 -0.51784

O 1.84249 -1.59698 -1.33857

C 0.59464 -1.14197 -1.07854

C -0.34961 -1.75379 -0.27371

C -1.60655 -1.17557 -0.09659

C -2.56052 -1.72466 0.76972

C -3.44857 -2.04088 1.53385

C -4.47589 -2.35116 2.40473

C -5.55659 -1.58466 2.71090

C -5.88163 -0.32110 2.23438

N -6.17371 0.72362 1.84934

C -6.35496 -2.35266 3.76189

C -5.45807 -2.38197 4.98871

C -4.40437 -3.12822 4.68885

C -4.58276 -3.61067 3.25846

C -6.11238 -3.78004 3.24040

C -1.95296 0.03368 -0.74861

O -3.20630 0.47878 -0.50015

C -3.56823 1.81247 -0.79367

C -2.84260 2.86736 0.04615

C -3.18989 2.81921 1.53588

C -2.62427 1.61839 2.29609

C -1.09794 1.56900 2.33922

C -0.60451 0.40178 3.18568

C -1.01525 0.63537 -1.56934

C 0.25068 0.07011 -1.72669

C 1.25557 0.69334 -2.47738

C 2.24220 1.14521 -3.02061

C 3.39980 1.63874 -3.59269

C 4.65484 1.12547 -3.48742

C 5.07335 -0.00340 -2.79527

N 5.44764 -0.93485 -2.23178

C 5.58311 2.08048 -4.23418

C 5.55036 3.36708 -3.42555

C 4.32673 3.86634 -3.52874

C 3.52432 2.92142 -4.40897

C 4.63465 2.47919 -5.37847

H 3.56859 2.73331 -0.84757

H 4.71068 3.52416 0.24183

H 3.09328 3.10195 0.80963

H 5.27669 1.11864 0.00614

H 4.77880 1.47523 1.66105

H 2.48674 0.63761 1.13585

H 3.00679 0.30362 -0.52000

H 4.22711 -0.96267 1.96597

H 4.66798 -1.32064 0.29230

H 1.85022 -1.72519 1.37762

H 3.09379 -2.97823 1.46256

H 1.79862 -3.48016 -0.47946

H 3.35885 -2.83310 -1.04109

H -0.13317 -2.67572 0.24334

H -7.37821 -2.02057 3.91204

H -5.66714 -1.83806 5.88976

H -3.53763 -3.34509 5.28314

H -3.96288 -4.44542 2.94418

H -6.52014 -3.95539 2.24430

H -6.46516 -4.55133 3.92478

H -4.63827 1.84078 -0.57239

H -3.42457 2.02022 -1.86324

H -1.76400 2.77064 -0.08528

H -3.13776 3.84240 -0.34858

H -2.81945 3.73675 2.00118

H -4.27663 2.80340 1.63994

H -2.99864 1.66661 3.32206

H -3.00818 0.69809 1.85681

H -0.69452 1.46060 1.33019

H -0.71515 2.50629 2.75188

H 0.48322 0.36584 3.19577

H -0.97962 -0.53808 2.78537

H -0.95404 0.49852 4.21140

H -1.23310 1.55573 -2.08876

H 6.56338 1.68525 -4.48475

H 6.38233 3.73006 -2.85348

H 3.91062 4.73872 -3.06256

H 2.59665 3.30808 -4.82104

H 5.01981 3.29607 -5.98845

H 4.34803 1.64386 -6.01832

**NBD-P2 (QC-QC form)**

Atom x y z

C -0.97270 -2.70801 1.60519

C -1.09898 -1.37256 2.32783

C -2.55989 -0.95297 2.47404

C -2.75517 0.33565 3.27417

C -2.17770 1.59306 2.62090

C -2.93682 2.03581 1.36650

O -2.78912 1.16952 0.26246

C -1.58248 1.03261 -0.34739

C -0.50050 1.88827 -0.22614

C 0.67954 1.63114 -0.91957

C 1.82027 2.44356 -0.77959

C 2.86405 3.04491 -0.69568

C 4.13235 3.64254 -0.62336

C 4.99894 4.02327 0.56734

C 6.23354 3.47401 -0.13958

C 6.33722 4.26735 -1.40537

C 5.56276 5.55438 -1.29431

C 4.47983 5.08956 -0.35585

C 5.38881 3.08515 -1.34377

C 5.44778 1.94472 -2.15367

N 5.50949 1.00769 -2.81393

C 0.79586 0.50771 -1.76555

O 1.99306 0.38600 -2.39682

C 2.36265 -0.83647 -2.99695

C 2.59198 -1.98119 -2.00555

C 3.83895 -1.79891 -1.13769

C 3.74572 -0.67448 -0.10534

C 2.69725 -0.91733 0.97790

C 2.71481 0.18697 2.02760

C -0.28983 -0.34203 -1.89520

C -1.46577 -0.09147 -1.19252

C -2.57135 -0.96038 -1.25220

C -3.55442 -1.65938 -1.19624

C -4.71646 -2.42797 -1.02330

C -4.90975 -3.89613 -0.67570

C -6.02655 -3.53184 0.29679

C -7.05406 -2.83507 -0.54055

C -6.87291 -3.17062 -1.99766

C -5.37965 -3.36982 -2.00270

C -5.85403 -2.05705 -0.03507

C -5.89893 -0.93578 0.80203

N -5.94493 -0.02628 1.50096

H -1.48699 -3.49186 2.15797

H 0.07286 -2.99056 1.50094

H -1.41523 -2.64024 0.61348

H -0.64072 -1.44903 3.31783

H -0.54913 -0.61773 1.76311

H -3.10273 -1.75243 2.98589

H -3.00503 -0.83573 1.48635

H -2.29105 0.21544 4.25731

H -3.82643 0.48508 3.42375

H -2.23393 2.41505 3.33859

H -1.12549 1.43599 2.37859

H -2.64403 3.05883 1.09141

H -4.01230 2.02057 1.56235

H -0.54330 2.76031 0.40809

H 4.78478 3.94109 1.61701

H 7.03125 2.91942 0.32058

H 7.22025 4.20033 -2.02207

H 5.16480 5.88282 -2.25551

H 6.16563 6.35686 -0.86522

H 3.70550 5.76302 -0.02411

H 3.29948 -0.59853 -3.50778

H 1.61796 -1.13372 -3.74864

H 2.70643 -2.89655 -2.59114

H 1.71233 -2.10120 -1.37128

H 4.68795 -1.59140 -1.79226

H 4.03640 -2.74091 -0.61801

H 4.72415 -0.57439 0.37267

H 3.53241 0.26558 -0.61350

H 1.70226 -0.95731 0.53134

H 2.88666 -1.88085 1.45923

H 1.95807 0.00555 2.78808

H 3.68678 0.23580 2.51478

H 2.51550 1.14969 1.56138

H -0.24748 -1.21332 -2.53050

H -4.17084 -4.66218 -0.52870

H -6.20007 -3.98174 1.25759

H -8.03039 -2.61469 -0.13712

H -7.18407 -2.35539 -2.65244

H -7.41218 -4.07767 -2.27691

H -4.85828 -3.63937 -2.90753

**NBD-O1 (NBD-NBD form)**

Atom x y z

C 5.25813 -3.07085 2.08693

O 5.37532 -1.70198 1.78946

C 4.27082 -1.03540 1.36571

C 3.02120 -1.59520 1.19170

C 1.94536 -0.81989 0.74713

C 0.68139 -1.38212 0.52188

C -0.42967 -1.78919 0.25329

C -1.70280 -2.25717 -0.01416

C -2.61646 -1.77304 -0.89512

C -2.49355 -0.70977 -1.77747

N -2.41972 0.17770 -2.50758

C -3.84828 -2.66907 -0.78919

C -3.39647 -4.02287 -1.30878

C -2.50535 -4.49661 -0.44916

C -2.35096 -3.46652 0.65635

C -3.79663 -2.94264 0.72393

C 2.13608 0.55074 0.49174

C 1.03719 1.32878 0.10311

C 0.02325 1.92777 -0.18966

C -1.11394 2.63543 -0.53190

C -2.41390 2.32818 -0.28045

C -2.93194 1.24582 0.41192

N -3.38482 0.36051 0.99372

C -3.25851 3.41924 -0.93438

C -3.02808 3.24723 -2.42637

C -1.76112 3.55124 -2.66618

C -1.12695 3.93152 -1.33821

C -2.33155 4.61898 -0.67243

C 3.40577 1.11367 0.65923

C 4.46839 0.34432 1.08777

O 5.73435 0.79599 1.28205

C 6.00913 2.14986 1.02249

H 6.24996 -3.38725 2.40536

H 4.95875 -3.65052 1.20734

H 4.54418 -3.24751 2.89830

H 2.84854 -2.64209 1.38498

H -4.76062 -2.25991 -1.21258

H -3.73495 -4.44797 -2.23446

H -1.93473 -5.40447 -0.49878

H -1.87464 -3.80115 1.57327

H -3.90163 -2.03839 1.32115

H -4.51134 -3.69926 1.04772

H -4.29070 3.47709 -0.60030

H -3.77510 2.88393 -3.10372

H -1.21684 3.49974 -3.58857

H -0.18148 4.46500 -1.38169

H -2.64734 5.52424 -1.19063

H -2.18263 4.83270 0.38704

H 3.52775 2.16511 0.45241

H 5.81806 2.40655 -0.02482

H 7.06845 2.28008 1.23721

H 5.42492 2.81152 1.67081

**NBD-O1 (NBD-QC form)**

Atom x y z

C -4.68627 -2.78854 -3.45468

O -4.77264 -1.41074 -3.19323

C -3.79977 -0.83520 -2.43578

C -2.71823 -1.49846 -1.89213

C -1.77381 -0.81409 -1.12032

C -0.68257 -1.48093 -0.54235

C 0.26805 -1.98527 0.01668

C 1.37448 -2.56970 0.60757

C 1.88579 -4.89450 1.03773

C 2.50609 -4.56315 2.16161

C 3.16524 -3.21475 1.93054

C 3.55155 -3.36549 0.44897

C 2.12472 -3.77376 0.04143

C 2.00733 -2.23226 1.75947

C 1.69839 -1.22907 2.66523

N 1.47717 -0.40132 3.43495

C -1.91725 0.56687 -0.91110

C -0.93676 1.25359 -0.17012

C -0.05565 1.79930 0.44987

C 0.98398 2.41640 1.16312

C 1.10599 2.77901 2.63537

C 2.55918 2.32181 2.64338

C 3.23847 3.17499 1.62069

C 2.43621 4.41999 1.34593

C 1.05879 3.86400 1.59903

C 2.46182 1.95054 1.17008

C 2.99251 0.85369 0.48876

N 3.43816 -0.04528 -0.07094

C -3.01068 1.23693 -1.46282

C -3.95161 0.55774 -2.21327

O -5.04947 1.11922 -2.78502

C -5.26514 2.49647 -2.60879

H -4.72398 -3.37793 -2.53222

H -5.55415 -3.02538 -4.06794

H -3.77422 -3.03836 -4.00737

H -2.58209 -2.55781 -2.04185

H 1.27634 -5.75346 0.83034

H 2.52939 -5.08420 3.09980

H 3.93137 -2.91588 2.64024

H 4.28772 -4.15036 0.27505

H 3.87746 -2.43056 -0.00475

H 1.92515 -3.99607 -1.00262

H 0.39818 2.61958 3.42603

H 3.03313 1.76690 3.43090

H 4.31401 3.17493 1.53190

H 2.55848 4.77603 0.32172

H 2.68952 5.22662 2.03623

H 0.18262 4.48571 1.50336

H -3.09982 2.29746 -1.28776

H -4.44849 3.09059 -3.03273

H -6.18687 2.71956 -3.14363

H -5.38941 2.75364 -1.55142

**NBD-O1 (QC-QC form)**

Atom x y z

C -5.82228 -2.68804 -0.13991

O -5.74250 -1.28709 -0.08411

C -4.50827 -0.71166 -0.04595

C -3.31051 -1.39919 -0.05646

C -2.09216 -0.71660 -0.01461

C -0.87200 -1.41972 -0.01209

C 0.18955 -1.99493 -0.01245

C 1.43009 -2.65251 0.00891

C 1.80592 -4.07522 0.39091

C 3.06140 -3.56891 1.09289

C 3.84166 -2.86771 0.02510

C 3.39997 -3.30407 -1.34512

C 1.96188 -3.61610 -1.03116

C 2.70691 -2.13676 0.71863

C 2.83527 -0.95831 1.45691

N 2.95257 0.00095 2.07804

C -2.08536 0.68375 0.03224

C -0.85832 1.37459 0.05789

C 0.20680 1.94287 0.07548

C 1.45162 2.59305 0.09257

C 2.26425 3.15217 1.25019

C 3.54837 2.62328 0.62419

C 3.62830 3.28741 -0.71300

C 2.76714 4.52276 -0.74840

C 1.69699 4.08148 0.21619

C 2.75672 2.05536 -0.54580

C 2.91885 0.84896 -1.22875

N 3.06861 -0.13985 -1.79418

C -3.29731 1.37899 0.04674

C -4.50158 0.70402 0.01052

O -5.73054 1.29221 0.02197

C -5.79683 2.69383 0.07745

H -6.88575 -2.92064 -0.16385

H -5.34535 -3.08467 -1.04286

H -5.37038 -3.15436 0.74238

H -3.28831 -2.47689 -0.09537

H 1.17579 -4.87799 0.72811

H 3.45620 -3.93372 2.02429

H 4.85572 -2.54777 0.20781

H 3.95058 -4.17846 -1.69635

H 3.48896 -2.49457 -2.06900

H 1.29153 -3.97860 -1.79403

H 2.03228 3.14632 2.29795

H 4.35728 2.15927 1.15633

H 4.52586 3.21708 -1.30825

H 2.37042 4.72454 -1.74472

H 3.30563 5.40371 -0.39417

H 0.87134 4.72979 0.46387

H -3.26468 2.45640 0.08622

H -6.85809 2.93754 0.07903

H -5.33509 3.08476 0.99073

H -5.32142 3.15610 -0.79458

**NBD-O2 (NBD-NBD form)**

Atom x y z

C -8.58872 1.37858 1.16318

C -7.80488 1.09754 -0.11272

C -6.54914 1.96011 -0.20961

C -5.75143 1.66267 -1.47589

C -4.46211 2.47747 -1.54745

C -3.63821 2.11026 -2.78461

O -3.20918 0.76522 -2.78516

C -2.07876 0.41158 -2.12188

C -1.30846 1.24098 -1.33248

C -0.14601 0.76620 -0.71648

C 0.61624 1.58048 0.13140

C 1.31907 2.19740 0.90482

C 2.10476 2.92629 1.77768

C 2.94390 2.47362 2.74658

C 3.20080 1.16822 3.13236

N 3.42135 0.09142 3.47791

C 3.60674 3.70270 3.36420

C 4.46335 4.28282 2.25143

C 3.64070 4.72537 1.31199

C 2.22273 4.44820 1.78381

C 2.41269 4.67048 3.29423

C 0.25801 -0.56531 -0.92731

C 1.46034 -1.00749 -0.35915

C 2.53571 -1.28892 0.12734

C 3.74235 -1.62907 0.70985

C 4.79150 -0.82351 1.01918

C 4.93655 0.53794 0.79689

N 5.07365 1.67000 0.63612

C 5.86523 -1.71638 1.63679

C 6.31606 -2.62810 0.50883

C 5.29306 -3.41530 0.20673

C 4.14613 -3.04088 1.12934

C 4.94175 -2.68355 2.39751

C -0.53146 -1.40916 -1.71413

C -1.69298 -0.94737 -2.29959

O -2.53427 -1.67074 -3.07878

C -2.36753 -3.06581 -3.20619

C -2.69552 -3.87846 -1.94874

C -4.14462 -3.74009 -1.47620

C -4.48623 -2.39193 -0.83705

C -3.72775 -2.12632 0.46241

C -4.12936 -0.79592 1.08560

H -7.97614 1.17643 2.03936

H -8.90012 2.42038 1.19817

H -9.47711 0.75336 1.21377

H -8.44155 1.28689 -0.97987

H -7.51816 0.04373 -0.13849

H -5.92153 1.77770 0.66589

H -6.83286 3.01545 -0.19791

H -6.36507 1.88188 -2.35274

H -5.50087 0.60076 -1.50632

H -4.69413 3.54351 -1.59262

H -3.87234 2.29943 -0.64557

H -2.78347 2.78832 -2.89954

H -4.26705 2.19483 -3.67615

H -1.58680 2.26919 -1.16279

H 4.09057 3.54256 4.32371

H 5.53483 4.25564 2.25239

H 3.87361 5.15118 0.35586

H 1.42415 4.98138 1.27543

H 1.55798 4.35370 3.89380

H 2.67923 5.69702 3.54513

H 6.64257 -1.19724 2.18933

H 7.28288 -2.56891 0.04632

H 5.21667 -4.15819 -0.56439

H 3.33066 -3.75382 1.20952

H 4.34255 -2.18591 3.15824

H 5.47103 -3.53521 2.82494

H -0.19726 -2.42378 -1.86423

H -1.34805 -3.30012 -3.54308

H -3.06258 -3.33797 -4.00591

H -2.02149 -3.60376 -1.13796

H -2.50490 -4.92567 -2.19563

H -4.34116 -4.53118 -0.74740

H -4.80856 -3.90348 -2.32850

H -5.55836 -2.38305 -0.62093

H -4.28948 -1.59077 -1.54914

H -3.92797 -2.93628 1.16877

H -2.65246 -2.10597 0.27514

H -3.89149 0.02029 0.40794

H -5.19732 -0.77452 1.29253

H -3.59340 -0.63249 2.01816

**NBD-O2 (QC-QC form)**

Atom x y z

C 3.41759 1.82825 1.03834

C 2.41868 2.97621 1.10061

C 2.14073 3.40387 2.54079

C 1.17384 4.58551 2.64816

C -0.24462 4.30113 2.14888

C -1.06623 3.40068 3.07868

O -0.53106 2.11595 3.29863

C -0.51299 1.19940 2.29293

C -1.14578 1.32509 1.07203

C -1.05709 0.31499 0.11144

C -1.68914 0.45377 -1.13943

C -2.22030 0.55222 -2.21915

C -2.82951 0.65446 -3.48053

C -4.25602 0.36362 -3.92024

C -3.74572 -0.27713 -5.20459

C -2.97393 0.80173 -5.89440

C -3.37461 2.15895 -5.37821

C -3.73013 1.76936 -3.96673

C -2.30893 0.00401 -4.78670

C -1.16824 -0.78735 -4.93019

N -0.23093 -1.43805 -5.06434

C -0.32205 -0.84415 0.39295

C -0.23576 -1.86576 -0.57234

C -0.17051 -2.71365 -1.42941

C -0.12963 -3.71465 -2.41341

C 0.18172 -5.19986 -2.32224

C -0.93429 -5.50786 -3.31463

C -0.56256 -4.75748 -4.55536

C 0.89988 -4.40494 -4.55881

C 1.10778 -4.28555 -3.07335

C -1.26145 -4.02543 -3.42458

C -2.49822 -3.38381 -3.51918

N -3.52315 -2.87151 -3.60190

C 0.30784 -0.97827 1.63243

C 0.22121 0.02004 2.58268

O 0.77442 -0.01200 3.82690

C 1.42067 -1.18067 4.27989

C 2.87899 -1.27865 3.82256

C 3.71640 -0.13403 4.38791

C 5.15470 -0.16861 3.87972

C 5.97044 1.01218 4.39992

C 7.39980 0.99041 3.87275

H 3.01793 0.95921 1.55479

H 3.61695 1.54962 0.00571

H 4.35777 2.10963 1.50838

H 2.80683 3.83031 0.53897

H 1.49483 2.65178 0.61826

H 1.74743 2.55954 3.10618

H 3.08673 3.69835 3.00446

H 1.12094 4.90286 3.69246

H 1.57758 5.42050 2.06861

H -0.19778 3.86551 1.15133

H -0.78662 5.24694 2.07106

H -2.09756 3.32839 2.70480

H -1.09658 3.84405 4.07857

H -1.72967 2.20080 0.83500

H -5.08143 0.01100 -3.33200

H -4.13566 -1.17078 -5.65417

H -2.63474 0.67036 -6.91056

H -2.55396 2.87721 -5.41650

H -4.22901 2.56482 -5.92301

H -4.08265 2.50413 -3.26016

H 0.43996 -5.78926 -1.46135

H -1.61752 -6.33661 -3.26127

H -1.09767 -4.92359 -5.47739

H 1.51192 -5.19066 -5.00509

H 1.08067 -3.45937 -5.06918

H 2.07476 -4.03926 -2.66461

H 0.86284 -1.88386 1.82147

H 1.38897 -1.10394 5.37129

H 0.85880 -2.07458 3.98096

H 2.92449 -1.25434 2.73167

H 3.28101 -2.23803 4.15518

H 3.25393 0.81363 4.10636

H 3.71408 -0.19089 5.47898

H 5.15377 -0.14919 2.78738

H 5.62888 -1.10230 4.19271

H 5.98512 0.98863 5.49192

H 5.48553 1.94306 4.09719

H 7.90628 0.07807 4.18060

H 7.40571 1.03317 2.78557

H 7.96259 1.83991 4.25284

# 12 Stern-Volmer plots

**Figure S139.** Stern-Volmer plots for the determination of the extinction coefficients of **NBD-P1** in toluene.

**Figure S140.** Stern-Volmer plots for the determination of the extinction coefficients of **NBD-P2** in toluene.

**Figure S141.** Stern-Volmer plots for the determination of the extinction coefficients of **NBD-O1** in toluene.

**Figure S142.** Stern-Volmer plots for the determination of the extinction coefficients of **NBD-O2** in toluene.

# 13 References

1 Y. Gunes, N. Arcelik, E. Sahin, F. F. Fleming and R. Altundas, *Eur. J. Org. Chem.*, 2015, **2015**, 6679–6686.

2 S. Fukushima, M. Ashizawa, S. Kawauchi and T. Michinobu, *Helv. Chim. Acta.*, 2019, **102**, e1900016.

3 Z. Shu, Q. Zhang, P. Zhang, Z. Qin, D. Liu, X. Gao, B. Guan, H. Qi, M. Xiao, Z. Wei, H. Dong and W. Hu, *Polym. Chem.*, 2020, **11**, 1572–1579.

4 J. Bucher, T. Stößer, M. Rudolph, F. Rominger and A. S. K. Hashmi, *Angew. Chem. Int. Ed.*, 2015, **54**, 1666–1670.

5 S. Godlewski, M. Engelund, D. Peña, R. Zuzak, H. Kawai, M. Kolmer, J. Caeiro, E. Guitián, K. P. C. Vollhardt, D. Sánchez-Portal, M. Szymonski and D. Pérez, *Phys. Chem. Chem. Phys.*, 2018, **20**, 11037–11046.

6 C. A. Basinger, K. Sullivan, S. Siemer, S. Oehrle and K. A. Walters, *J. Chem.*, 2015, **2015**, 672654.

7 R. R. Weber, C. N. Stindt, A. M. J. van der Harten and B. L. Feringa, *Chem. Eur. J.*, 2024, **30**, e202400482.

8 Z. Wang, H. Hölzel, L. Fernandez, A. S. Aslam, P. Baronas, J. Orrego-Hernández, S. Ghasemi, M. Campoy-Quiles and K. Moth-Poulsen, *Joule*, 2024, **8**, 2607–2622.

9 C. A. Gueymard, *Sol. Energy*, 2004, **76**, 423–453.

10 Z. Wang, A. Roffey, R. Losantos, A. Lennartson, M. Jevric, A. U. Petersen, M. Quant, A. Dreos, X. Wen, D. Sampedro, K. Börjesson and K. Moth-Poulsen, *Energy Environ. Sci.*, 2019, **12**, 187–193.
